# Structure and activity of the septal peptidoglycan hydrolysis machinery crucial for bacterial cell division

**DOI:** 10.1371/journal.pbio.3002628

**Published:** 2024-05-30

**Authors:** Yatian Chen, Jiayue Gu, Biao Yang, Lili Yang, Jie Pang, Qinghua Luo, Yirong Li, Danyang Li, Zixin Deng, Changjiang Dong, Haohao Dong, Zhengyu Zhang

**Affiliations:** 1 Department of Clinical Laboratory, Zhongnan Hospital of Wuhan University, School of Pharmaceutical Sciences, Wuhan University, Wuhan, China; 2 Key Laboratory of Combinatorial Biosynthesis and Drug Discovery, Ministry of Education, and School of Pharmaceutical Sciences, Wuhan University, Wuhan, China; 3 State Key Laboratory of Biotherapy and Cancer Center, National Clinical Research Center for Geriatrics, West China Hospital, Sichuan University, Chengdu, China; 4 The Cryo-EM Center, Core facility of Wuhan University, Wuhan University, Wuhan, China; Rutgers University-Robert Wood Johnson Medical School, UNITED STATES

## Abstract

The peptidoglycan (PG) layer is a critical component of the bacterial cell wall and serves as an important target for antibiotics in both gram-negative and gram-positive bacteria. The hydrolysis of septal PG (sPG) is a crucial step of bacterial cell division, facilitated by FtsEX through an amidase activation system. In this study, we present the cryo-EM structures of *Escherichia coli* FtsEX and FtsEX-EnvC in the ATP-bound state at resolutions of 3.05 Å and 3.11 Å, respectively. Our PG degradation assays in *E*. *coli* reveal that the ATP-bound conformation of FtsEX activates sPG hydrolysis of EnvC-AmiB, whereas EnvC-AmiB alone exhibits autoinhibition. Structural analyses indicate that ATP binding induces conformational changes in FtsEX-EnvC, leading to significant differences from the apo state. Furthermore, PG degradation assays of AmiB mutants confirm that the regulation of AmiB by FtsEX-EnvC is achieved through the interaction between EnvC-AmiB. These findings not only provide structural insight into the mechanism of sPG hydrolysis and bacterial cell division, but also have implications for the development of novel therapeutics targeting drug-resistant bacteria.

## Abstract 2

The septal peptidoglycan hydrolysis machinery is a crucial structure for bacterial cell division. This study analyses the cryo-EM structure and enzymatic function of E. coli FtsEX, with and without the lytic-factor protein EnvC, characterizing the molecular basis of FtsEX regulation of peptidoglycan hydrolysis, which could be used to develop novel treatments targeting drug-resistant bacteria.

## Introduction

Bacterial antibiotic resistance poses one of the greatest threats to global health [1–4]. Peptidoglycan (PG), an essential component of the cell wall in both gram-negative and gram-positive bacteria, surrounds the cytoplasmic membrane and maintains cell shape during cell growth and cell division [5–12]. Since PG is vital in bacteria but absents in human cells, it is a promising target for drug development [13,14]. Previous research has shown that bacteria assemble the divisome, a multiprotein complex, for cell division [15,16]. In *Escherichia coli*, the divisome consists of a series of essential proteins, including FtsZ, FtsA, ZipA, FtsE, FtsX, FtsW, FtsI, FtsQ, FtsB, FtsL, FtsK, and FtsN [15,17]. In the early stage of bacterial cell division, FtsZ, FtsA, and ZipA form the Z-ring at the future division site, acting as the core of the divisome [18]. In the later stage, other essential proteins are recruited to the Z-ring in a linear sequence to form the complete divisome [19–21]. FtsN, the last essential division protein to arrive at the division site, triggers the synthesis of septal peptidoglycan (sPG), resulting in cell constriction. FtsEX, an important component of the divisome, is mainly recognized for its involvement in activating the degradation of sPG through amidases via the periplasmic factor EnvC. FtsEX localizes to the division septum by interacting with the Z-ring [22–24], facilitating divisome assembly and activates sPG synthesis by the SEDS family PG polymerase FtsWI [15,21].

During the constriction phase of cell division, the separation of daughter cells does not occur until the newly synthesized sPG is hydrolyzed. Importantly, 3 amidases, AmiA, AmiB, and AmiC, have been found to be essential for the *E*. *coli* cell separation process [25–28]. These amidases are autoinhibited to prevent cell lysis resulting from aberrant cleavage of the PG layer [26–28]. The activation of these amidases is stimulated by activator proteins containing LytM domains, through the interface interactions [27,29,30]. The regulation of sPG hydrolysis is mediated by FtsEX pathway [22–24]. To relieve autoinhibition, FtsEX acts on its LytM factor EnvC to activate AmiA and AmiB, which is the most critical pathway for proper cell separation in *E*. *coli* [22,24,27,29]. In addition, the LytM factor NlpD participates in cell separation by activating the third amidase, AmiC. NplD-AmiC becomes critical for cell separation if the EnvC-AmiA/AmiB pathway is absent [29,31]. The activation of AmiA and AmiB by EnvC is controlled by the cell division protein complex FtsEX, which induces conformational changes in EnvC through ATP hydrolysis [23,24,26,27,32].

FtsEX belongs to the Type VII ABC superfamily, which includes LolCDE and MacB [33–37]. One of the most distinctive features of this subfamily is that their transmembrane region consists of 8 transmembrane helices, unlike the typical 12 transmembrane helices found in other members [34]. Recent studies have reported that LptBFGC and LolCDE do not transport substrates across the bacterial inner membrane but extract lipopolysaccharide or lipoproteins from the membrane [37,38]. In contrast to the reported ABC transporters, FtsEX neither transports anything across the membrane nor extracts substances from the membrane but interacts with EnvC to form the sPG hydrolysis machinery to separate the daughter cells during cell division [22,23,39]. Studies have shown that FtsEX is conditionally essential for cell division [40–42]. For instance, deletion of *ftsEX* results in filamentation and death in *E*. *coli* when grown in medium with low osmotic pressure [40,41,43], likely due to defects in divisome assembly and the activation of cell constriction. Recently, Cook and colleagues determined X-ray crystal structures of FtsX (periplasmic domain)-EnvC and an activated form of AmiB enzymatic domain bound to LytM domain [24,27]. Xu and colleagues and Hao and colleagues reported cryo-EM structures of FtsEX-EnvC from different species, which have provided a model for amidase activating driven by FtsEX [32,44]. Here, we present the cryo-EM structures of *ec*FtsEX and *ec*FtsEX-EnvC at resolutions of 3.05 Å and 3.11 Å, respectively. Using an *in vitro* amidase activation assay, we also revealed the mechanism of sPG hydrolysis regulation by the FtsEX-EnvC complex through ATP binding.

## Results

### Overall structure of ATP-bound FtsEX and FtsEX-EnvC

Previous studies have shown that mutation on E163 residue would result in the FtsE mutant protein no longer hydrolyzing ATP but still binding to it [40,45]. In this study, we constructed the FtsE^E163Q^ mutant, which was expected to abolish ATPase activity but retain ATP-binding ability. FtsE^E163Q^X was successfully expressed and purified in the presence of ATP, while the sPG hydrolysis machinery complex FtsE^E163Q^X-EnvC with ATP was further obtained by in vitro reconstitution (S1A Fig).

**Fig 1:**
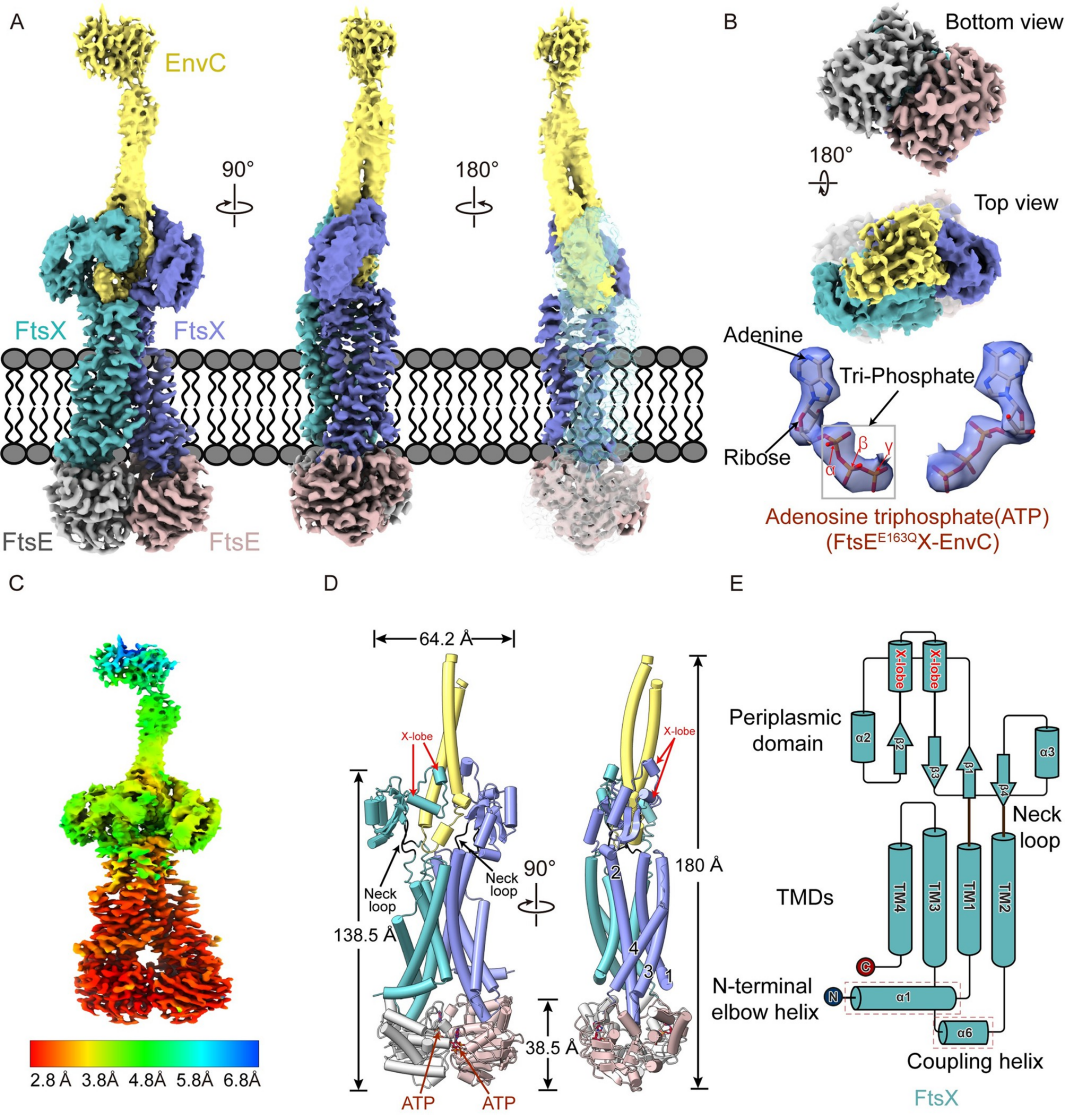
Cryo-EM structure of ATP-bound FtsE^E163Q^X-EnvC. Two copies of FtsE, 2 copies of FtsX, and a single copy of EnvC are colored grayish red, gray, purple, lime green, and soft yellow, respectively. ATP molecules are displayed as spheres and colored in red by element. The map is colored by molecule carving at 5 Å, and the micelle of the PMAL-C8 is hided. The map level ranges from 0.05 to 0.2 by ChimeraX, with 0.05 for EnvC, 0.1 for FtsX periplasmic domain, and 0.2 for the rest part. (**A**) Cryo-EM map of the FtsE^E163Q^X-EnvC complex, viewed from the front, side, and back. (**B**) Top and bottom views of the FtsE^E163Q^X-EnvC cryo-EM map, and the ATP bound to FtsE^E163Q^X-EnvC. The map for ATP is carved at 2 Å. (**C**) The cryo-EM local resolution maps of FtsE^E163Q^X-EnvC are colored in rainbow by resolution. (**D**) Cartoon representation of the FtsE^E163Q^X-EnvC model complex in front and side views. (**E)** Topological diagram of the secondary structure of a single FtsX.

We then determined the cryo-EM structure of ATP-bound *E*. *coli* FtsE^E163Q^X and ATP-bound FtsE^E163Q^X-EnvC at overall resolutions of 3.05 Å and 3.11 Å, respectively, using the gold standard Fourier shell correlation based on the 0.143 criterion (Figs 1A, 1B, and S1–S3). For the FtsE^E163Q^X-EnvC map, the local resolution varies throughout the complex (Fig 1C). The core domains, including the transmembrane and nucleotide binding domains, are determined at 2.8 Å, while the periplasmic domains of FtsX are determined at 3.8 to 4.5 Å. Notably, the cryo-EM map of the restraining arm and LytM domain of EnvC are almost invisible (Fig 1A–1C). Notably, the cryo-EM map of the FtsX periplasmic domain in FtsE^E163Q^X-EnvC is significantly clearer than that of FtsE^E163Q^X. This suggests that the periplasmic domains of FtsX can be stabled when bound with EnvC, which helped in resolving the cryo-EM map of these regions. Based on the cryo-EM map of FtsEX-EnvC, we built a model incorporating FtsE, FtsX, and EnvC coiled-coil domain. For FtsEX cryo-EM map, we built a model including FtsE, FtsX (without the periplasmic domain). Consequently, the structure of the complete FtsE^E163Q^X-EnvC complex consists of 2 copies of FtsE, 2 copies of FtsX, and a single EnvC (Fig 1A and 1B). The dimensions of the FtsE^E163Q^X complex were determined to 138 Å in length and 64 Å in width (Fig 1D). The structures showed that FtsEX adopts a similar fold to its family members LolCDE and MacB (S4 Fig). Our results revealed that a single FtsX molecule exhibits a topological structure comprising 4 transmembrane helices (TMs) named TM1-TM4 (Fig 1E). TM1 extends into the periplasm, while TM2 heads toward another FtsX and forms a large periplasmic domain with TM1. The periplasmic domain of FtsX is connected to the TMs through 2 flexible neck loops (Fig 1E). Between TM2 and TM3, there is a helix α3 corresponding to the coupling helix. The X-lobe structure is situated between TM1 and TM2, which is the basis of the interaction between FtsX and EnvC. The FtsEX complex structure shows a severely disordered periplasmic structure (S5 and S6A Figs), indicating that this domain is highly flexible. The flexibility of the periplasmic region of FtsEX-EnvC could be related to the motility of the complex, which enables it to better hydrolyze sPG during daughter cell separation.

### EnvC binding causes significant conformational changes

**Fig 2:**
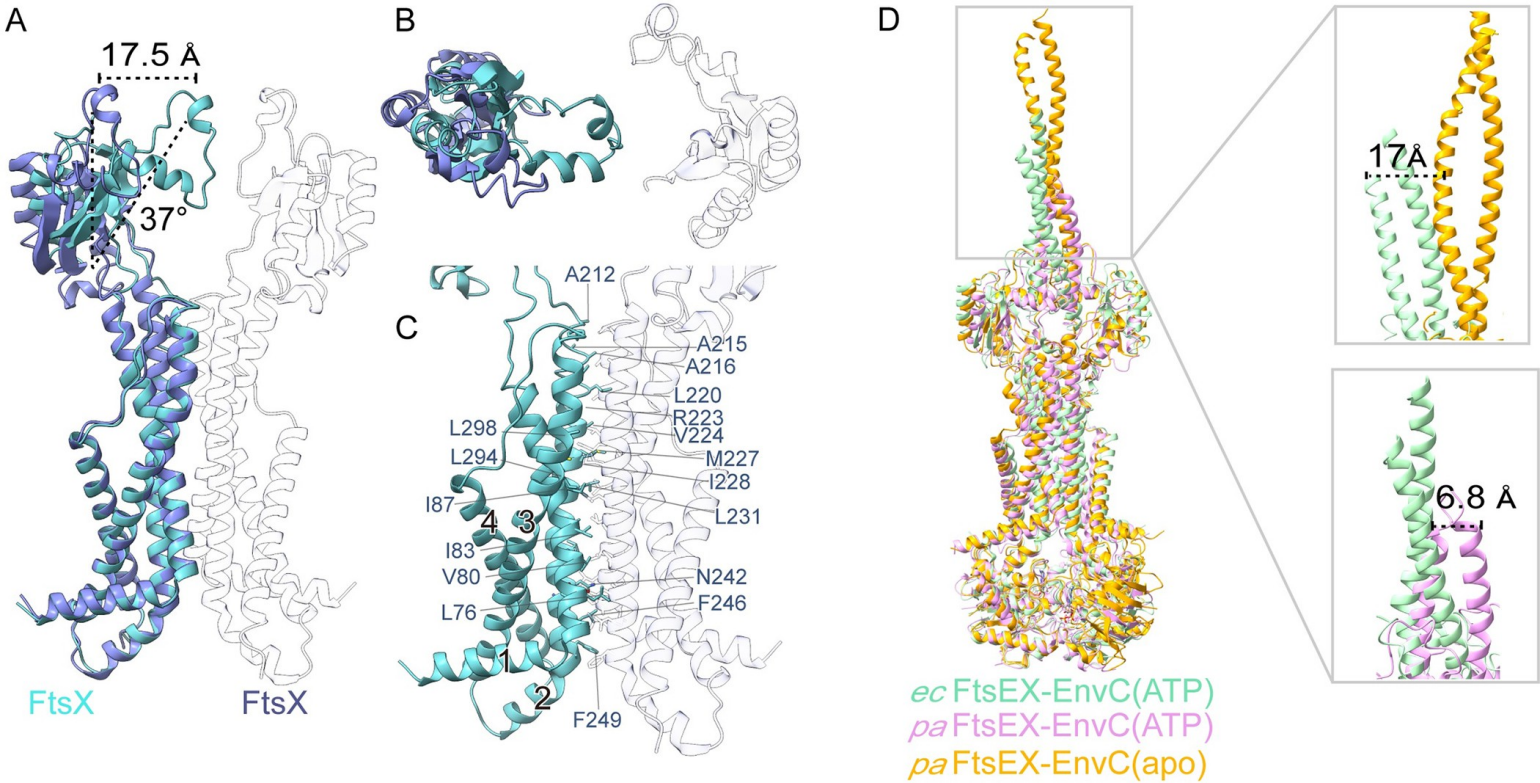
Asymmetric FtsX of FtsE^E163Q^X-EnvC adopts conformational changes and ATP binding lead to FtsEX-EnvC conformational changes. (**A**) An overall superimposition of 2 FtsX from the *ec*FtsE^E163Q^X-EnvC complex. (**B**) A top view of *ec*FtsX superimposition. (C) Dimeric *ec*FtsX is formed mainly through the interactions between TM1&3 and TM2. (D) Superimposition of the *ec*FtsE^E163Q^X-EnvC (ATP) (cyan) with *pa*FtsEX-EnvC(apo) (PDB ID: 8I6O) (yellow) and *pa*FtsEX-EnvC(ATP) (PDB ID: 8I6S)(plum).

We observed that in the ATP-bound FtsE^E163Q^X-EnvC complex structure, the 2 copies of FtsX appeared to be asymmetric (Fig 2A). According to the superimposition of the 2 FtsX from the FtsEX-EnvC complex, the transmembrane helices of the 2 FtsX molecules are almost identical in conformation, but the periplasmic domain of one FtsX, compared to the other, shifts approximately 17.5 Å and rotates 37° (Fig 2A–2C). We speculate that the binding of EnvC results in a relatively rigid conformation of the FtsX periplasm domain, while the transmembrane domain of FtsX is almost identical.

Recently, Xu and colleagues published structures of FtsEX-EnvC from *Pseudomonas aeruginosa* in different states [32]. When our structure *ec*FtsE^E163Q^X-EnvC(ATP) and the *pa*FtsEX-EnvC(apo), *pa*FtsEX-EnvC(ATP) structures are superimposed using one FtsE as a reference, we observe overall similarity between our *ec*FtsE^E163Q^X-EnvC(ATP) and the *pa*FtsEX-EnvC(apo) structures, with conformational differences are observed on the EnvC coiled-coil domain (Fig 2D). The result of superimposition suggests that the conformational state of EnvC in *ec*FtsE^E163Q^X-EnvC(ATP) is relatively similar to that in *pa*FtsEX-EnvC(ATP) (Fig 2D). Compared to the previously reported crystal structure of the FtsX (periplasmic domain)-EnvC complex (PDB ID:6TPI), *ec*FtsE^E163Q^X-EnvC displays conformational changes in one of the FtsX periplasmic domains, when the other FtsX periplasmic domains are superimposed (S6A Fig). The superimposition results suggest that the 2 periplasmic domains of FtsX in *ec*FtsE^E163Q^X-EnvC are closer to each other, while those of the FtsX (periplasmic domain)-EnvC structure are more separated (S6B Fig). In addition, conformational differences are observed in the coiled-coil domain of EnvC (EnvC_35-222_). However, due to the flexibility and small molecular weight of the EnvC LytM domain, the cryo-EM model determined in this study only covers part of the coiled-coil domain (Fig 1A and 1B). When our structure is superimposed with the FtsX (periplasmic domain)-EnvC structure using one FtsX (periplasmic domain) as a reference, the helix of EnvC on the reference FtsX side remains almost identical, while the other helix shifts down. The 2 X-lobe structures of FtsX tightly bound to the CC domain, which couples the conformational changes between FtsX and EnvC (S6B Fig). Significantly, EnvC is not static in the complex according to our 3D variability analysis in CryoSPARC (S1 Movie) [46]. EnvC and the periplasmic domain of FtsX can swing back and forth due to the extreme flexibility of the FtsX neck loop and EnvC CC domain. Although this flexibility makes it difficult to resolve a clear cryo-EM map, it could help the LytM domain of EnvC cover as much space as possible to increase amidase activation and PG hydrolysis efficiency when cells divide. When our structure is superimposed with different state of *Streptococcus pneumoniae* FtsE using one FtsE as reference, minimal changes are observed in the other FtsE (S6C and S6D Fig). Based on the results of FtsE alignments, we believe that FtsE, as nucleotide-binding domain, are relatively conservative in different species (S7 Fig).

### ATP-bound FtsEX-EnvC but not FtsX-EnvC activates AmiB to degrade PG

**Fig 3:**
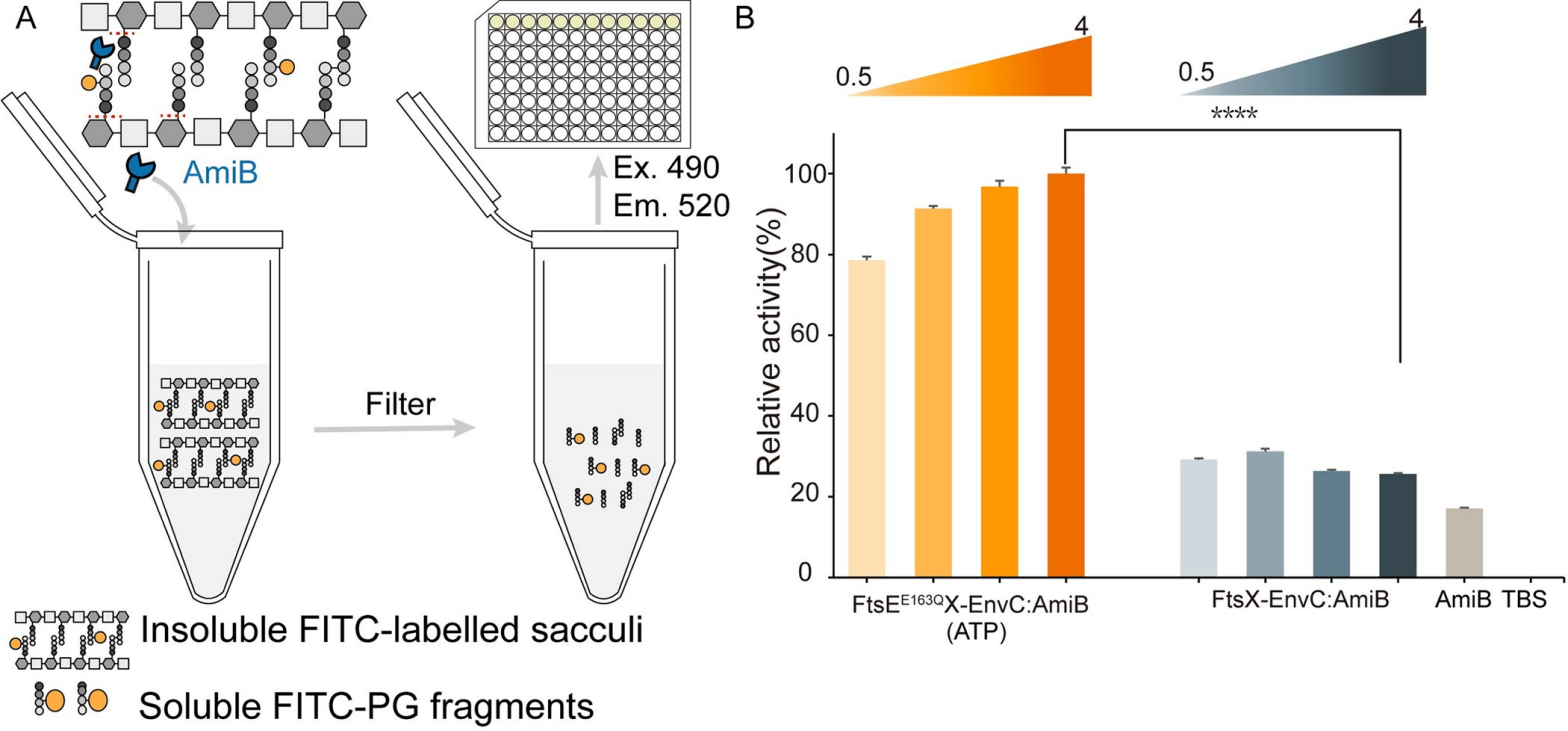
PG degradation assay. (**A**) Schematic diagram showing the degradation of FITC-labeled sacculi by AmiB. (**B**) PG degradation assay representing amidase activity. FtsE^E163Q^X-EnvC and FtsX-EnvC regulate AmiB hydrolysis of PG. The final concentration ratio of FtsE^E163Q^X-EnvC:AmiB were 0.5/1/2/4:1 in μM. The final concentration ratios of FtsX-EnvC:AmiB were the same, and the FtsX-EnvC:AmiB ratios were 0.5/1/2/4:1 in μM. The graph reveals that FtsE^E163Q^X-EnvC increased the activity of AmiB, while FtsX-EnvC could not. Four asterisks signify a *P*-value of less than 0.0001. The data underlying the graphs shown in the figure can be found in S2 Data. FITC, fluorescein isothiocyanate; PG, peptidoglycan.

Precious studies have elucidated the mechanisms of peptidoglycan regulation by FtsEX, revealing different modes of activation. In *P*. *aeruginosa*, it is activated by ATP binding [32], while in *Mycobacterium tuberculosis*, it is via ATP hydrolysis [47]. Interestingly, in *Vibrio cholerae*, the activation of AmiB does not depend on nucleotides [44]. Furthermore, dye release assays have demonstrated that EnvC, NplD, and ActS can regulate the amidases degradation of PG [29–31,48–50]. Amidases can hydrolyze amide bonds between the lactyl group of MurNAc and the L-alanine residue of purified fluorescein isothiocyanate (FITC)-labeled PG sacculi [48,49]. After cleavage, FITC-peptide fragments released from PG sacculi become soluble in the supernatant, and filtration can remove insoluble PG sacculi (Fig 3A). To investigate the mechanism by which FtsEX regulates AmiB hydrolysis through EnvC in *E*. *coli*, we conducted experiments using purified AmiB, EnvC, FtsE^E163Q^X-EnvC, and FtsX-EnvC, incubating them with FITC-labeled PG. Our findings revealed that AmiB alone is inactive and has minimal ability to cleave PG sacculi, which aligns with previous research [29]. Furthermore, it was observed that FtsX-EnvC or FtsE^E163Q^X-EnvC alone displayed no amidase activity, thus conforming previous reports that EnvC requires the presence of AmiB to degrade PG [29]. However, when EnvC, FtsX-EnvC, or FtsE^E163Q^X-EnvC were added together with AmiB, the amidase activity was detected (S9A Fig). Notably, the addition of FtsX-EnvC or EnvC to the reaction did not significantly alter the AmiB activity, whereas the addition of FtsE^E163Q^X-EnvC resulted in a significant increase (S8A and S8B Fig). Furthermore, it was found that FtsE^E163Q^X-EnvC (ATP) had a similar effect to FtsEX-EnvC (AMP-PNP) (S8C Fig). These findings suggest that FtsX-EnvC, like EnvC, only moderately activates AmiB, while ATP-bound FtsE^E163Q^X-EnvC strongly enhances AmiB activity. Additionally, these results indicate that EnvC, whether alone or in complex with FtsX, adopts the autoinhibitory conformation [24,32].

In order to further clarify the activation of AmiB by FtsE^E163Q^X-EnvC, a serial gradient experiment was conducted. The final molar concentration of AmiB was fixed at 1 μM, while the concentration of the regulators FtsE^E163Q^X-EnvC or FtsX-EnvC ranged from 0.5 to 4 μM. The results showed that increasing the ratio of FtsE^E163Q^X-EnvC to AmiB enhanced the degradation of PG (Fig 3B). However, the amidase activity remained unchanged regardless of the ratio of FtsX-EnvC to AmiB. These results provide additional evidence that ATP-bound FtsE^E163Q^X-EnvC can activate AmiB, which aligns more closely with the mechanism observed in *P*. *aeruginosa*. However, neither EnvC nor FtsX-EnvC showed significant activation of AmiB, indicating that EnvC in *E*. *coli* may adopts a more restrictive self-inhibitory conformation to prevent abnormal splitting.

### Interactions between EnvC and AmiB is essential for amidase activation

To investigate the regulatory role of EnvC on AmiB more extensively, we constructed 6 AmiB mutants, namely on AmiB^Y324A^, AmiB^L325N^, AmiB^A328D^, AmiB^V329D^, AmiB^L332N^, and AmiB^ΔY324-332^, based on Cook and colleagues’ previous work [27]. To test the hydrolyzing activity of the AmiB mutants, we purified all mutant proteins and incubated them with FITC-labeled PG. When FtsE^E163Q^X-EnvC (ATP) was introduced to the reaction, the hydrolyzing activity of all AmiB mutant proteins decreased, while the wild-type AmiB protein remained almost as high as the positive control lysozyme (S8D and S8E Fig). These in vitro PG hydrolysis assays suggest that FtsEX-EnvC regulates AmiB activity by the interaction between EnvC and AmiB. Our *in vitro* findings are consistent with the *in vivo* results of Cook and colleagues [27].

### The interaction between FtsE and FtsX are essential for FtsEX to function

**Fig 4:**
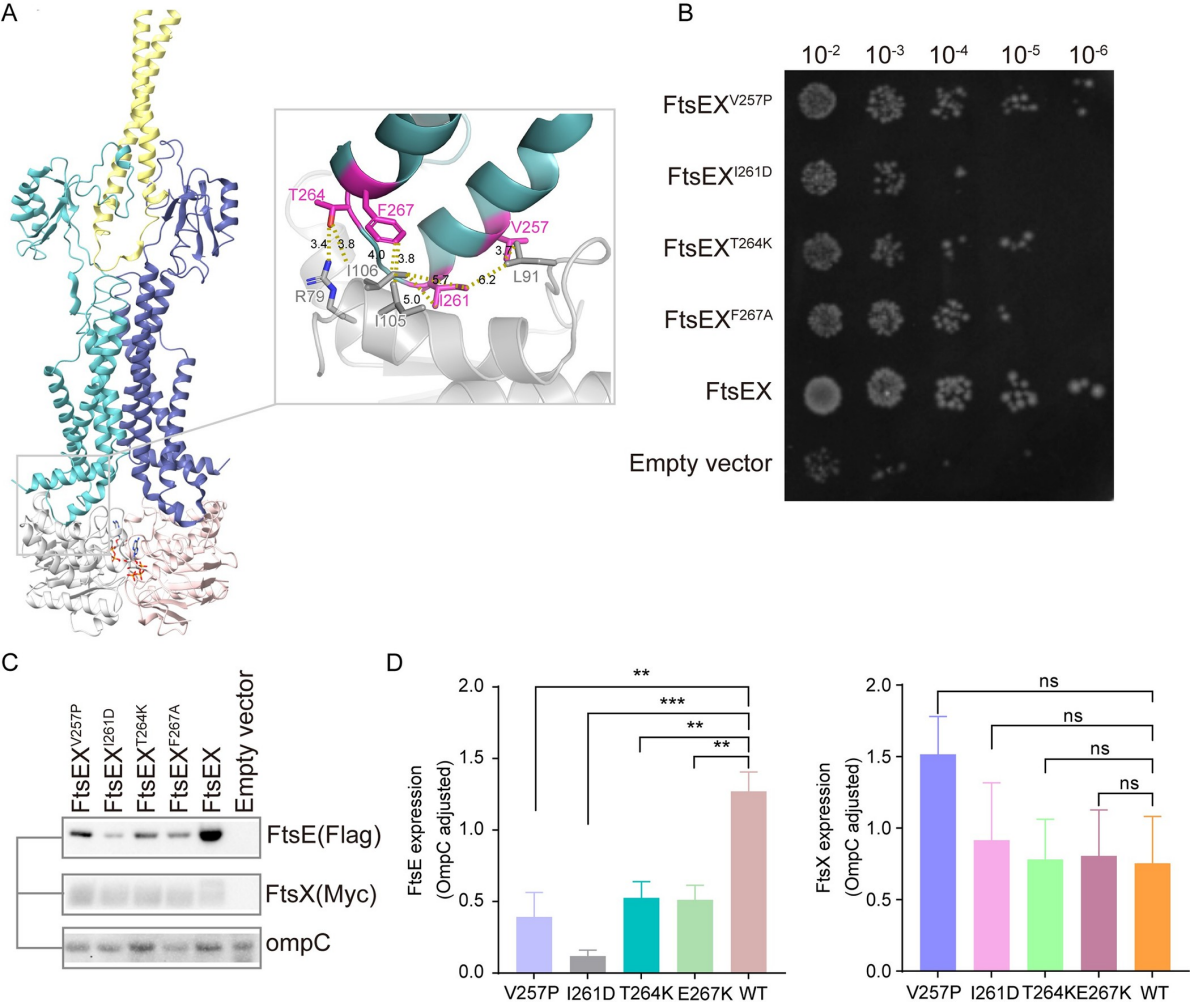
FtsE and FtsX interaction is critical for bacterial viability. (**A**) The overall structure of FtsEX-EnvC and the selected mutagenesis residues. The presentation colors for FtsEX-EnvC are the same as those in Fig 1. The interaction distances are labeled in Å. (**B**) Functional assay results of these mutants. (**C**) Western blot analysis of FtsE and FtsX on membrane. (D) The quantification of expression levels of FtsE and FtsX in the FtsEX mutants, and the OmpC expression level were used as the control. Three asterisks signify a *P*-value of less than 0.001. The data underlying the graphs shown in the figure can be found in S2 Data and S1 Raw Images.

The interaction between FtsE and FtsX primarily occur through the coupling helix, a fundamental component of ABC transporters that links the NBD and TMD to drive conformational changes in the TMD and other subunits upon ATP consumption. Previous research has identified 4 FtsX mutants (FtsX^E213K^, FtsX^I216K^, FtsX^L219K^, and FtsX^V220K^) in *Streptococcus pneumoniae* that affect the FtsE-FtsX interaction based on predicted and FtsX based on predicted model. To determine the interaction residues between FtsE and FtsX in our structures, we used ChimeraX in this study [51]. We generated FtsX mutants, including FtsX^V257P^, FtsX^I261D^, FtsX^T264K^, and FtsX^F267A^, and conducted functional assays (Fig 4A). FtsX^V257P^ and FtsX^I261D^ in *E*. *coli* corresponding to the mutant FtsX^I216K^ and FtsX^V220K^ in *S*. *pneumoniae* (Fig 4B), they show the similar growth defects as reported by Alcorio and colleagues in their paper [52]. Furthermore, severe growth defects were observed in FtsX^T264K^ and FtsX^F267A^ mutants. Interestingly, sequence alignments revealed that these interaction residues are relatively conserved among different species, implying a conserved function of FtsX across species (S9 Fig). Our western blot analysis of the membrane fraction showed that the level of FtsE (without mutations) on the membrane in the presence of these FtsX mutants decreased dramatically, while that of FtsX (with mutations) did not, indicating that our mutations reduced the binding between FtsE and FtsX (Fig 4C). Our results strongly support the conclusion of Du and colleagues that the location of FtsE and FtsX in membrane is codependent [17]. According to the study of Arends and colleagues and Corbin and colleagues, FtsX was able to localize without the presence of FtsE, but FtsE cannot localize without FtsX [40,53]. Mutant FtsX^I261D^ displayed the most severe phenotype in vivo, and very little FtsE was retained on the membrane when it was co-expressed with FtsX^I261D^ as determined by western blotting (Fig 4C). Overall, these results suggest that the complex interaction between FtsE and FtsX is important for their functions.

## Discussion

Bacterial cell division has been considered one of the most promising targets for drug development for decades [13,14]. In *E*. *coli*, PG hydrolases, including AmiA, AmiB, and AmiC, were reported to be autoinhibited and to need activators, such as EnvC or NplD, to function [22,26–28]. Several studies have shown that PG hydrolases in *S*. *pneumoniae*, *E*. *coli*, *Bacillus subtilis*, and *M*. *tuberculosis* are somewhat similar and that sPG cleavage in these organisms is similarly regulated and activated through the sPG hydrolysis machinery FtsEX-lytic factor [22,23,54,55]. Deletions of *ftsE* and/or *ftsX* in bacteria such as *E*. *coli*, *Neisseria gonorrhoeae*, and *Aeromonas hydrophila* caused division defects [40,56,57], confirming the broad conservation of FtsEX across species (S7 and S9 Figs). In *S*. *pneumoniae*, FtsEX regulates the activity of PcsB to directly cleave sPG, while in *E*. *coli*, the corresponding roles of the single protein PcsB are split into 2 proteins, EnvC and AmiB. Another study revealed that in *B*. *subtilis*, CwlO was involved in cell wall cleavage during cell elongation under the regulation of FtsEX. Although hydrolases vary among different bacterial species, the role of FtsEX in sPG hydrolysis seems to be conserved.

In this study, we expressed and purified the complete FtsEX and FtsEX-EnvC complex, both of which harbored an E163Q mutation in FtsE. Subsequently, we determined the cryo-EM structure of the ATP-bound FtsEX-EnvC complex at a resolution of 3.11 Å. Prior research has elucidated several various mechanisms through which FtsEX regulates amidase activity. In *P*. *aeruginosa*, it is activated by ATP binding [32], in *M*. *tuberculosis* it is via ATP hydrolysis [47], whereas the activation of AmiB does not depend on nucleotides in *V*. *cholerae* [44]. Our study unveils the crucial role of ATP binding in the regulation of EnvC by FtsEX, leading to the activation of AmiB. Interestingly, this activation mechanism bears resemblance to the process observed in *P*. *aeruginosa*. Additionally, previous research has demonstrated that AmiB avoids aberrant hydrolysis of sPG by spatial segregation [32,44,47]. According to the structure of FtsX(periplasm)-EnvC and ATP-bound FtsE^E163Q^X-EnvC, there might also exist spatial blocking mechanism in *E*. *coli* as well. Interestingly, in our *in vitro* PG hydrolysis experiments, the activation of AmiB by EnvC alone is quite weak in *E*. *coli*, which is different from that in *P*. *aeruginosa*. These results suggest that in addition to the spatial blocking mechanism, aberrant hydrolysis is also avoided through stringent autoinhibition of EnvC in *E*. *coli*. According to the crystal structure of the AmiB enzymatic domain bound to the EnvC LytM domain (PDB:8COJ), we identified the key residues affecting the interaction between AmiB and EnvC [27]. In this study, we performed in vitro PG hydrolysis using the AmiB mutants AmiB^Y324A^, AmiB^L325N^, AmiB^A328D^, AmiB^V329D^, AmiB^L332N^, and AmiB^ΔY324-332^. These experiments confirmed that the hydrolytic activity of AmiB is regulated by FtsEX-EnvC through the interaction between AmiB and EnvC.

**Fig 5:**
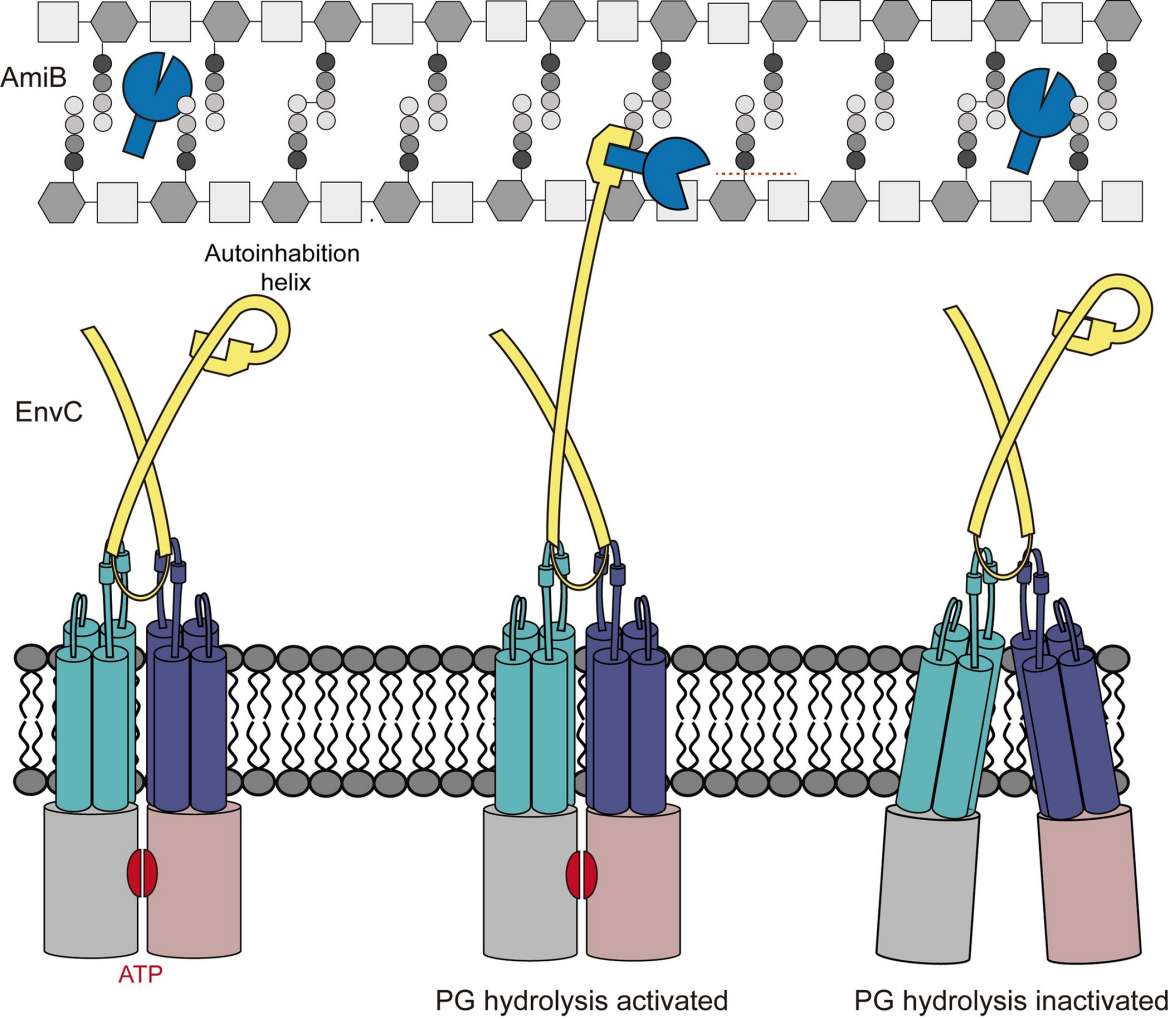
A proposed mechanism of divisome FtsEX-EnvC in the regulation of PG hydrogenation. In *E*. *coli*, FtsEX regulates EnvC to activate AmiB. FtsE, grayish red and gray; FtsX, purple and lime green; EnvC, soft yellow; AmiB, dark blue. In the ATP-bound state, the NBDs will dimerize, and the conformational changes in TMDs will drive EnvC to change its conformation, exposing the LytM domain. The exposed LytM domain of EnvC can bind to AmiB and stimulate it to hydrolyze PG. The apo conformation of the PG hydrolysis machinery would cause the LytM domain of EnvC to return to an autoinhibitory state. Since the autoinhibitory LytM domain cannot bind to AmiB, PG hydrolysis then ceases. PG, peptidoglycan.

Although we were unable to construct apo FtsEX *in vitro* from *E*. *coli*, valuable information can still be gleaned from the cryo-EM structure of FtsEX in different states in *P*. *aeruginosa* and the crystal structure of LytM-amidase. These structures offer insight into how ATP binding and hydrolysis of FtsEX-EnvC activate amidase [27,32]. These results strongly support the mechano-transmission mechanism. The conformational changes occur during ATP binding or hydrolysis, which leads to the release of the restraining arm on EnvC. The interaction between the LytM domain of EnvC and the interaction helix on AmiB directs the hydrolysis of septal PG. Through a comparison of *ec*FtsE^E163Q^X-EnvC and FtsX (periplasmic domain)-EnvC, we observed conformational changes in the coiled-coil domain of EnvC (S6B Fig). The observed conformational changes in EnvC might serve as the basis for understanding how it displace its restraining arm and recognizes AmiB, ultimately leading to the activation of sPG hydrolyzation (Fig 5).

*E*. *coli*, an important model species, has been extensively studied for its division process. Our structure, combined with PG hydrolysis assays, provides insight into the molecular mechanism by how FtsEX-EnvC activates AmiB to hydrolyze PG in *E*. *coli*, which is similar to that observed in *P*. *aeruginosa*. However, *ec*EnvC exhibits strong autoinhibition and is unable to efficiently activate AmiB-mediated PG hydrolysis *in vitro*. This observation suggests that the hydrolysis of septal PG in *E*. *coli* is tightly regulated. We hypothesize that this strict control of amidase activity in *E*. *coli*, a fast-growing organism, effectively prevents aberrant hydrolysis of septal PG.

## Materials and methods

### Plasmids construction

Gene fragments encoding FtsEX were amplified by polymerase chain reaction (PCR) from *E*. *coli* K-12 genomic DNA. The 2 PCR products were then cloned into a modified pET28a plasmid, which contained an N-terminal eight-histidine tag and a SUMO fusion followed by a tobacco etch virus (TEV) protease cleavage site, yielding a plasmid named pSUMO-*ftsEX*. Two fragments FtsE^E163Q^ and FtsX^Δ4–18^ were amplified from pSUMO-*ftsEX* and cloned into pSUMO plasmid in the same way to generate the construct pSUMO-*ftsE*^*E163Q*^*X*^*Δ4–18*^. pSUMO-*ftsE*^*E163Q*^*X* is generated in the same way. Similarly, all other constructs in this study were generated. pSUMO-*envC*_*35-420*_ was constructed with a Strep tag II on the C terminus. The full-length *ftsX* gene was amplified from *E*. *coli* K-12 genomic DNA and inserted into pET28a plasmid with an octa-histidine (8 × His) tag at the N terminus yielding the pET28a-*ftsX*. The fragment of *amiB*_*23-445*_ gene was amplified from *E*. *coli* K-12 genomic DNA and inserted into pSUMO yielding the pSUMO-*amiB*_*23-445*_. The full-length of *ftsE* and *ftsX* gene were amplified from pSUMO-*ftsEX* and cloned into a pTrc99a plasmid with a Flag tag on the N terminal of FtsE and an 8 × His tag followed by Myc tag on the C terminal of FtsX, yielding a plasmid named pTrc99a-*ftsEX*.

### Expression and purification of FtsE^E163Q^X^Δ4–18^ and FtsE^E163Q^X

*E*. *coli* C43(DE3) (Weidi) harboring pSUMO-*ftsE*^*E163Q*^*X*^*Δ4–18*^ and pSUMO-*ftsE*^*E163Q*^*X* were used for protein expression. The transformed soluble C43(DE3) cells were grown in Luria broth (LB) supplemented with antibiotic (kanamycin 50 μg ml^−1^) at 37°C until the optical density of the culture reached 0.8 at a wavelength of 600 nm (OD_600_). The proteins were induced by addition of 0.1 mM isopropyl β-d-thiogalactopyranoside (IPTG) and incubated for 12 h at 20°C. Cells pellets were collected by centrifugation and resuspended in buffer A (20 mM Tris-HCl (pH 8.0), 300 mM NaCl) containing 0.1 mM PMSF. The cells were broken using a cell homogenizer at 800 bar (ATS SCIENCE INC). The cell debris was removed by centrifugation at 18,000×g for 20 min at 4°C. The supernatant was subjected to ultracentrifugation at 140,000×g for 1 h to collect the membrane fractions. The membrane was solubilized with buffer B (20 mM Tris-Cl (pH 8.0) and 300 mM NaCl, 10 mM imidazole) supplemented with 1% (w/v) n-dodecyl-β-D-maltopyranoside (DDM) (Anatrace) and 1 mM ATP (sigma) at 4°C for 1 h. After a further step of centrifugation at 30,000×g for 30 min, the supernatant was collected and loaded onto a 5 ml HisTrap HP column (Cytiva). The column was washed with buffer C (20 mM Tris-Cl (pH 8.0), 300 mM NaCl, 40 mM imidazole) supplemented with 0.05% DDM and 1 mM ATP. The FtsE^E163Q^X^△4–18^ and FtsE^E163Q^X complex were eluted with buffer D (20 mM Tris-Cl (pH 8.0), 300 mM NaCl, 300 mM imidazole) supplemented with 0.05% DDM and 1 mM ATP. The eluted protein was concentrated in Amicon Stirred Cell (Merck Millipore) with 30 kDa molecular weight cutoff Omega Ultrafiltration Membrane Disc Filter (Pall Corporation, United States of America). The further purification was performed by size-exclusion chromatography (Superdex-200 Increase 10/300 GL; Cytiva) in the buffer A supplemented with 0.05% DDM and 1 mM ATP at the flow rate of 0.75 ml min^−1^ using AKTA go (Cytiva). The peak fractions were collected and then removed the SUMO-tag by TEV protease. Tag-free FtsE^E163Q^X^△4–18^ and FtsE^E163Q^X complex were purified again using size-exclusion chromatography in buffer A. Protein fractions with the highest purity were pooled and concentrated to 1 mg ml^−1^.

### Expression and purification of FtsX

*E*. *coli* C43(DE3) (Weidi) harboring pSUMO-*ftsX* was used for protein expression. The expression and purification process of FtsX protein is the same as FtsE^E163Q^X^Δ4–18^ without addition of ATP or fusion-protein tag removing.

### Expression and purification of soluble protein EnvC, AmiB, and AmiB mutants

EnvC_35-420_, AmiB_23-445_, and AmiB_23-445_ mutants were soluble cytoplasmic protein and expressed and purified as the following method. *E*. *coli* C43(DE3) (Weidi) harboring pSUMO-*envC* or pSUMO-*amiB* was used for protein expression. The transformed soluble C43(DE3) cells were grown in LB supplemented with antibiotic (kanamycin 50 μg ml^−1^) at 37°C until the optical density of the culture reached 0.8 at a wavelength of 600 nm (OD_600_). The proteins were induced by addition of 0.1 mM IPTG and incubated for 12 h at 20°C. Cells pellets were collected by centrifugation and resuspended in buffer A (20 mM Tris-HCl (pH 8.0), 300 mM NaCl) containing 0.1 mM PMSF. The cells were broken using a cell homogenizer at 800 bar (ATS SCIENCE INC). The cell debris was removed by centrifugation at 18,000×g for 20 min at 4°C. The targeted proteins in supernatant were purified by a Ni-NTA column (Cytiva). The protein buffer was changed to buffer A using size-exclusion chromatography (Superdex-200 Increase 10/300 GL, Cytiva). The 8× His-SUMO tag was removed by TEV protease, and the His-tagged fragment was removed by a Ni-NTA column. Final protein samples were further purified using size-exclusion chromatography in buffer A.

### Reconstruction and purification FtsE^E163Q^X^Δ4-18^-EnvC, FtsE^E163Q^X-EnvC, and FtsX-EnvC complex in PAML-C8

Purified FtsE^E163Q^X^△4–18^ complex, FtsE^E163Q^X, or FtsX protein was incubated with the purified EnvC_35-420_ protein at a molar ratio of 1:1.5. After 2 h, the proteins were mixed with PMAL-C8 at 1:3 (w/w) with gentle agitation for another 2 h. Detergent was removed with Bio-Beads SM-2 (4°C overnight, 15 mg per 1 ml channel/detergent/amphipols mixture). SM-2 bio-beads were then removed by centrifugation and the supernatant was loaded onto 1 ml strep column (Smart-Lifescience) to remove unbound FtsE^E163Q^X^△4–18^ and FtsE^E163Q^X, the eluted protein was concentrated and loaded onto a Superdex 200 column in buffer A supplemented with 1 mM ATP, FtsE^E163Q^X^△4-18^-EnvC, FtsE^E163Q^X-EnvC, and EnvC would fraction into 2 separate peaks, and the peak corresponding to FtsE^E163Q^X^△4-18^-EnvC or FtsE^E163Q^X-EnvC was collected and validated by SDS-PAGE for further experiment. The FtsX protein were loaded onto 1 ml strep column (Smart-Lifescience) to remove unbound FtsX and the eluted protein were loaded onto a Superdex 200 column in buffer A. The FtsX-EnvC and EnvC would fraction into 2 separate peaks, and the peak corresponding to FtsX-EnvC was collected and validated by SDS-PAGE for further experiment.

### Site mutagenesis and functional assays

All single or double mutations were generated following the site-directed mutagenesis protocol of Liu and Naismith [58]. All mutations were amplified from pTrc99a-*ftsEX* with a Flag tag on the N terminal of FtsE and an 8 × His tag followed by Myc tag on the C terminal of FtsX. These mutants were transformed into *E*. *coli ftsEX* deletion strain WZC1 derived from SH26 (a gift from Shishen Du) by P1 transduction. The transformed WZC1 cells were grown on LB agar plates supplemented with 0.2 M sucrose 100 μg ml^−1^ ampicillin and cultured at 37°C for 12 h. Subcultured cells were used for the functional assays. The *E*. *coli* WZC1 transformed with empty plasmid pTrc99a was used as the negative control while cells with plasmid pTrc99a-FtsE(Flag)X(His&Myc) was used as positive control. Cells pellets were collected, washed twice with LB without NaCl, and diluted to the OD_600_ nm of 0.5 by LB without NaCl. Tenfold serial dilution functional assays were performed. The dilution range was from 10^−1^ to 10^−6^ and the diluted cells were dripped onto the LB agar plates without NaCl containing ampicillin (100 μg ml^−1^). Cell growth was recorded after overnight culture at 37°C. All assays were performed in triplicate. The expression of FtsEX mutants were detected by western blotting.

### Western blotting

The protein expression levels of FtsE and FtsX were determined using western blotting. For the experiment, WZC1 cells containing FtsEX, an FtsEX mutant, or an empty plasmid were cultured overnight. These cells were then inoculated into 10 ml LB supplemented with 0.2 M sucrose, 100 μg ml^−1^ ampicillin, and 0.1 μm IPTG. After being cultured at 37°C for 6 h, the cells were harvested by centrifugation at 6,000g, 4°C for 15 min. To prepare the cell lysate, the cell pellets were resuspended in 0.5 ml buffer A and sonication for 5 min on ice. The cell debris was then removed by centrifugation at 13,000×g for 20 min at 4°C. The resulting supernatant was subjected to ultracentrifugation at 140,000×g for 1 h to collect the membrane fractions. These membrane fractions were mixed with 5 × SDS-PAGE loading buffer. Finally, a 10 μl sample was loaded onto a 4% to 12% SDS-PAGE gel and run for 50 min.

The proteins were transferred to a PVDF membrane and washed with TBS buffer. Then, the membranes were blocked in TBS buffered supplemented with 5% skim milk at 4°C overnight. The next step was incubating the membranes with either anti-Flag (1:1,000 dilution) (Sigma, Catalogue No: F1804) or anti-Myc monoclonal antibody (1:5,000 dilution) (Sigma, Catalogue No: 4439) at room temperature for 2 h. After incubation, the membranes were washed with TBST (20 mM Tris-HCl (pH 7.6), 150 mM NaCl, 0.05% Tween-20) 3 times and then incubated with goat anti-mouse IgG antibody (1:4,000 dilution) for 1 h. Finally, the membranes were washed with TBST for 3 times and TBS once before being incubated with ECL substrate for imagining. The images were acquired by Monad QickChemi, and all experiments were repeated at least 3 times.

### Cryo-EM sample preparation and data collection

Cryo-EM grids of FtsE^E163Q^X^△4-18^-EnvC and FtsE^E163Q^X^△4–18^ in PMAL-C8 were prepared using a Vitrobot Mark IV (Thermo Fisher Scientific). Three microliters of the purified sample at a concentration of approximately 1 mg ml^−1^ was applied to each Quantifoil holy carbon grid (R1.2/1.3, 300 mesh Cu). Prior to sample application, all grids were glow discharged for 50 s. Subsequently, the grids were frozen in liquid ethane using a Vitrobot Mark IV. The freezing process involved setting the Vitrobot Mark IV at 8°C and 100% humidity, with no wait time, 3 s blot time, and +3 blot force.

All grids were initially screened using a FEI Glacios 200 keV first. Cryo-EM images were then collected at liquid nitrogen temperature using a Titan Krios (Thermo Fisher Scientific) equipped with a K3 detector (Gatan) and a BioQuantum energy filter. Movies were collected in counting mode, with 40 total frames per movie in 3 s, 60 electrons per Å^2^ accumulated dose, and 0.84 Å physical pixel size. The magnification used was 81,000, and the defocus was controlled within the range of −1 and −3 μm. For more detailed information about the EM data collection parameters, please refer to Table 1.

**Table 1:** Cryo-EM structure determination parameters and model statistics.

	ATP-binding FtsEX	ATP-binding FtsEX-EnvC	
	PDB ID:8X61	PDB ID:8Y3X	
	EMDB ID:38077	EMDB ID:38906	
**Data collection and processing**			
Microscope	Titan krios	Titan krios	
Detector	K3	K3	
Magnification	105,000	105,000	
Voltage (KV)	300	300	
Electron exposure (e^−^/Å^2^)	60	60	
Defocus range (μm)	−1 to −3	−1 to −3	
Pixel size (Å)	0.84	0.84	
Symmetry imposed	C1	C1	
Initial particle images (no.)	921,332	4,039,317	
Final particle images(no.)	370,231	1,455,436	
Map resolution (Å)	3.05	3.11	
FSC threshold	0.143	0.143	
**Refinement**			
Map sharpening B factor (Å)	−110.4		−105.1
Model composition			
Non-hydrogen atoms	5,737		9,096
Protein residues	756		1,414
Ligands	2		2
B-factors (Å)			
Protein	25.29		52.65
Ligand	7.52		15.11
R.M.S. deviations			
Bond length (Å)	0.003		0.006
Bond angles (°)	0.616		0.709
Validation			
Molprobity score	1.59		1.93
Clash score	6.69		7.61
Ramachandran plot			
Favored (%)	96.62		91.07
Allowed (%)	3.38		8.64
Outliers (%)	0.00		0.29

### Cryo-EM structural determination

The cryo-EM data were processed using CryoSPARC [46]. For both FtsE^E163Q^X^△4–18^ and FtsE^E163Q^X^△4-18^-EnvC complex, the movies underwent initial preprocessed using CryoSPARC live inline preprocessing. In detail, Patch Motion Correction and Patch CTF estimation program was used to perform motion correction and contrast transfer function estimation respectively. Preprocessed movies were exported from CyroSPARC live work session into CyroSPARC workspace. To ensure data quality, the Manually Curate Exposures tool was used to exclude the exposures of a relative ice thickness over 1.5 or CTF Fit over 6 Å. Initial particle picking was carried out by Blob Picker, followed by a 2D classification to generate templates for template-based picking. Template-based particle picking was performed using Template Picker. Particles were extracted with a 4 times bin before 2D classification. Particles from good 2D classes were extracted and subjected to another round of 2D classification. After several rounds of particle extraction and 2D classification, good particles were selected and unbinned particles were extracted and 2D classified again.

For the FtsE^E163Q^X^△4–18^ complex, 921,332 particles with a box size of 360 pixels, selected from a dataset of 7,897 exposures, were used for ab initio 3D reconstruction. Following heterogeneous refinement helped to yield a good class with 370,231 particles. Homogeneous refinement and non-uniform refinement were then performed yielding a 3.05 Å map at FSC = 0.143 with C1 symmetry. The map was generated after post-processing with *B* factors of −110.4 Å. The data-processing details are summarized in S2 Fig.

For the FtsE^E163Q^X^△4-18^-EnvC complex, 3 datasets of 16,695 movies in total were processed together, and 4,039,317 particles of a box size of 576 pixels were used for ab initio 3D reconstruction. Following heterogeneous refinement helped to yield a good class with 1,455,436 particles. Homogeneous refinement and non-uniform refinement were then performed yielding a 3.11 Å map at FSC = 0.143 with C1 symmetry. The map was generated after post-processing with *B* factors of −105.1 Å. The data-processing details are summarized in S1 Fig.

Focused refinement of FtsE^E163Q^X^△4-18^-EnvC complex were performed using the local refinement in CyroSPARC. Particle subtraction was first used to remove the non-focused region with a corresponding mask generated by ChimeraX [51]. Then, the local refinement and 3D variability analysis was carried out to study the region of interest. All maps were finally sharpened within CryoSPARC.

### Cryo-EM model building and refinement

The Alphafold2 server (Phenix Colab) was utilized to generate individual models for FtsE, FtsX, and EnvC. The domains of these modeled coordinates were separately fitted into the cryo-EM maps using ChimeraX. In the case of the FtsE^E163Q^X^△4-18^-EnvC complex, after the initial model docking, Coot and ChimeraX were employed for further adjustments of certain model fragments within the map. The local refinement maps were combined into a single map for real-space refinement in Phenix [59]. All visual representations of densities and models were created using ChimeraX or Pymol [60].

### In vitro PG degradation assay

The PG sacculi were prepared from strain MG1655 as described previously [61]. In detail, 1 liter MG1655 cells grown at 37°C for 4 h and then harvest by centrifugation (6,000*×g*) at 4°C for 15 min. Cells pellets were resuspended in 15 ml TBS and then added into 15 ml of boiling 8% SDS. The samples were boiling for 30 min under vigorous stirring. After incubated at room temperature overnight, the sacculi were collected by centrifugation (140,000*×g*) at 25°C for 1 h. The pellets were washed with 35 ml of water for 4 times until the SDS concentration was <0.01% as determined using the method of Hayashi [62]. The pellets are suspended into 1 ml of TBS buffer supplemented with α-amylase (100 μg ml^−1^) at 37°C overnight. Then, pronase (200 μg ml^−1^) was added and incubated at 60°C for 2 h. The samples were treated with 1 ml 8% SDS again and incubated at 100°C for 15 min. Then, the samples were added with 33 ml water and washed 4 times as described above and finally resuspended into 0.5 ml TBS. Isolated PG sacculi were subsequently labeled with FITC [31,48]. In brief, 25 mg purified PG sacculi was mixed with 12.5 mg FITC in 0.5 M sodium bicarbonate buffer (pH 9.3). After 3 h of incubation in dark, the samples were washed with 30 ml sodium bicarbonate buffer and washed with water 3 times until residual FITC could no longer be detected. After washed with acetone, the samples were dried and resuspended into 1 ml of TBS buffer. A measure of 5 μl FITC-labeled sacculi were incubated at 37°C for 1 h with 1 μm purified amidase and, or regulators in 100 ml of TBS buffer (20 mM Tris-HCl (pH 8.0), 300 mM NaCl). Reactions were terminated by filtration through a 0.45 μm filter membrane to remove insoluble sacculi and the fluorescence of the soluble fraction were measured at Microplate reader (Ex.490, Em,520). In addition to soluble proteins EnvC and AmiB, FtsE^E163Q^X, FtsE^E163Q^X–EnvC, and FtsX-EnvC were purified in PMAL-C8. All experiments were repeated at least 3 times.

## Supporting information

S1 Fig Data collection of FtsE^E163Q^X-EnvC and simplified workflow of data processing. (**A**) SEC profile and SDS-PAGE gel of purified *E*. *coli* FtsEX-EnvC complex in PMAL-C8. (**B**) A representative cryo-EM microscope image. (**C**) Selected 2D class image. (**D**) A simplified flowchart of cryo-EM data processing. (**E**) Gold-standard FSC curves of the final cryo-EM maps of FtsEX-EnvC from CryoSPARC. (**F**) Direction distribution iteration. The data underlying the graphs shown in the figure can be found in S1 Raw Images.
(TIF)

S2 Fig Data collection of FtsE^E163Q^X and simplified workflow of data processing. (**A**) A representative cryo-EM microscope image. (**B**) Selected 2D class image. (**C**) A simplified flowchart of cryo-EM data processing. (**D**) Gold-standard FSC curves of the final cryo-EM maps of FtsE^E163Q^X. (**E**) Direction distribution iteration. (**F**) The overall cryo-EM maps of FtsEX are colored according to the local resolution.
(TIF)

S3 Fig Partial cryo-EM map for FtsE^E163Q^X-EnvC in this study. (**A**) The map of transmembrane helices. (**B**) The map of ATP.
(TIF)

S4 Fig ATP-bound FtsE^E163Q^X-EnvC is superimposed to different states of MacB and LolCDE. (**A**) ATP-bound FtsE^E163Q^X-EnvC (cyan) is superimposed to apo MacB (bright purple) (PDB ID:5GKO). (**B**) ATP bound FtsE^E163Q^X-EnvC (cyan) is superimposed to ATP-bound MacB (fuchsia) (PDB ID:5LJ7). (**C**) ATP-bound FtsE^E163Q^X-EnvC (cyan) is superimposed to ADP-bound LolCDE (dark blue) (PDB ID:7ARL). (**D**) ATP-bound FtsE^E163Q^X-EnvC (cyan) is superimposed to apo LolCDE (dark purple) (PDB ID:7ARI). (**E**) ATP-bound FtsE^E163Q^X-EnvC (cyan) is superimposed to AMP-PNP-bound LolCDE (light blue) (PDB ID:7ARK).
(TIF)

S5 Fig Cryo-EM structure of FtsE^E163Q^X complex. (**A**) Cryo-EM map of FtsE^E163Q^X complex, viewed from front and side. (**B**) A cartoon representation of FtsE^E163Q^X complex in front and top. Transmembrane helices 1, 2, 3, and 4 are labeled out.
(TIF)

S6 Fig ATP-bound FtsE^E163Q^X-EnvC is superimposed to ATP-bound FtsEX, FtsX (periplamic domain)-EnvC, and different states of FtsE. **(A**) *ec*ATP-bound FtsE^E163Q^X-EnvC is superimposed to *ec*ATP-bound FtsEX. (B) *ec*ATP-bound FtsE^E163Q^X-EnvC is superimposed to *ec*FtsX (periplamic domain)-EnvC. (C) *ec*ATP-bound FtsE^E163Q^X-EnvC is superimposed to *sp*ADP-bound FtsE(6Z4W). (D) *ec*ATP-bound FtsE^E163Q^X-EnvC is superimposed to *sp*AMP-PNP-bound FtsE(6Z67).
(TIF)

S7 Fig The alignment of FtsE in *E*. *coli* with other species. The resides of K41, D162, and E163 which are related to ATPase is highly conserved over different bacterial species.
(TIF)

S8 Fig PG degradation assay. **(A**) PG degradation assay representing amidase activity. The graph shows the degradation of FITC-labeled sacculi by AmiB and/or regulators. Lysozyme was used as positive control and TBS as negative control. Individual AmiB showed a slight activity. EnvC together with AmiB resulted in an increase in PG degradation. FtsE^E163Q^X-EnvC boosted the activity of AmiB to degrade PG compared to FtsX-EnvC. Four asterisks signify a *P*-value of less than 0.0001. (**B**) SDS-PAGE analysis of samples involved in the reaction of Fig 3B. (**C**) PG degradation assay of FtsEX and FtsE^E163Q^X. (**D**) PG degradation assays of AmiB and AmiB mutants. Four asterisks signify a *P*-value of less than 0.0001. (**E**) SDS-PAGE analysis of samples involved in the reaction of S8D Fig. The data underlying the graphs shown in the figure can be found in S2 Data and S1 Raw Images.
(TIF)

S9 Fig The alignment of FtsX in *E*. *coli* with other species. (TIF)

S1 Movie The 3D variability analysis of FtsE^E163Q^X-EnvC in CryoSPARC. (MP4)

S1 Data The PDB validation report of FtsE^E163Q^X and FtsE^E163Q^X-EnvC. (PDF)

S2 Data Numerical values used to generate graphs. (XLSX)

S1 Raw Images The raw images of Figs 4, S1, and S8. (PDF)

## Abbreviations

FITC: fluorescein isothiocyanate
IPTG: isopropyl β-d-thiogalactopyranoside
LB: Luria broth
PCR: polymerase chain reaction
PG: peptidoglycan
sPG: septal peptidoglycan
TEV: tobacco etch virus
