# Supplementary figures and images for: Structure and activity of the septal peptidoglycan hydrolysis machinery crucial for bacterial cell division
Source: PLoS Biol. 2024 May 30;22(5):e3002628. doi: 10.1371/journal.pbio.3002628 (PMC11139282; doi:10.1371/journal.pbio.3002628)

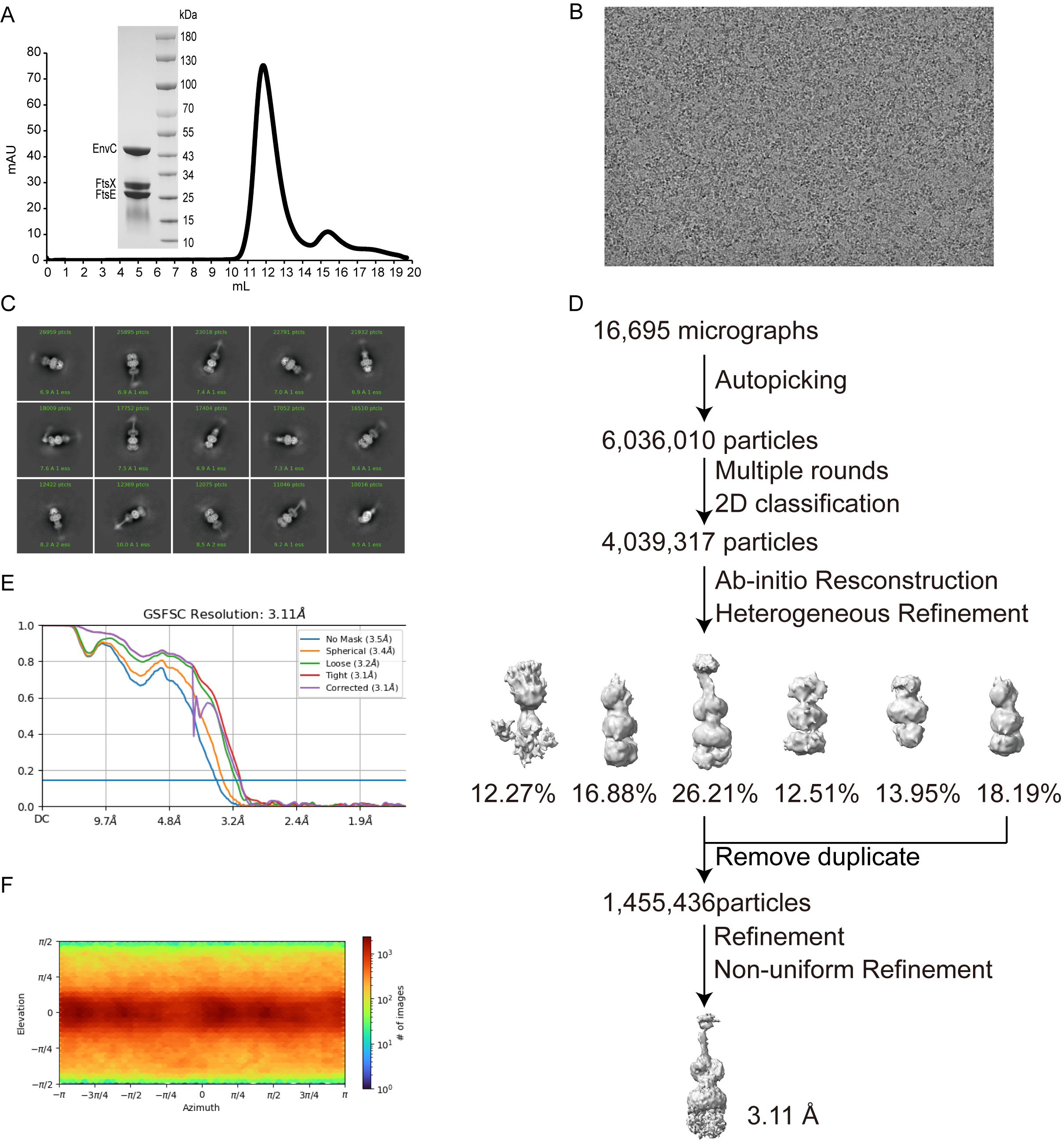

Supplement: S1 Fig — (A) SEC profile and SDS-PAGE gel of purified E. coli FtsEX-EnvC complex in PMAL-C8. (B) A representative cryo-EM microscope image. (C) Selected 2D class image. (D) A simplified flowchart of cryo-EM data processing. (E) Gold-standard FSC curves of the final cryo-EM maps of FtsEX-EnvC from CryoSPARC. (F) Direction distribution iteration. The data underlying the graphs shown in the figure can be found in S1 Raw Images. (TIF) [file pbio.3002628.s001.tif]

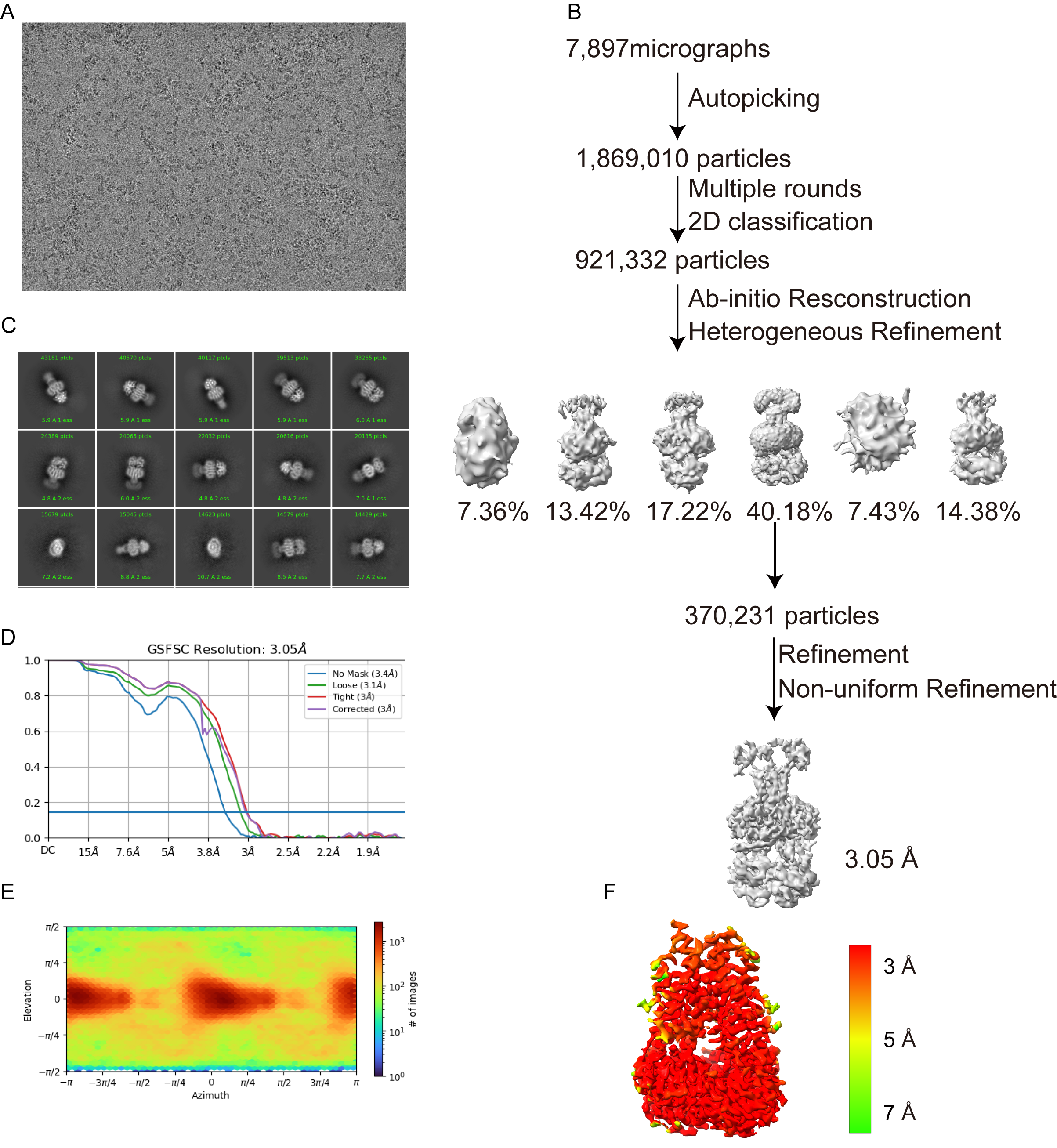

Supplement: S2 Fig — (A) A representative cryo-EM microscope image. (B) Selected 2D class image. (C) A simplified flowchart of cryo-EM data processing. (D) Gold-standard FSC curves of the final cryo-EM maps of FtsEE163QX. (E) Direction distribution iteration. (F) The overall cryo-EM maps of FtsEX are colored according to the local resolution. (TIF) [file pbio.3002628.s002.tif]

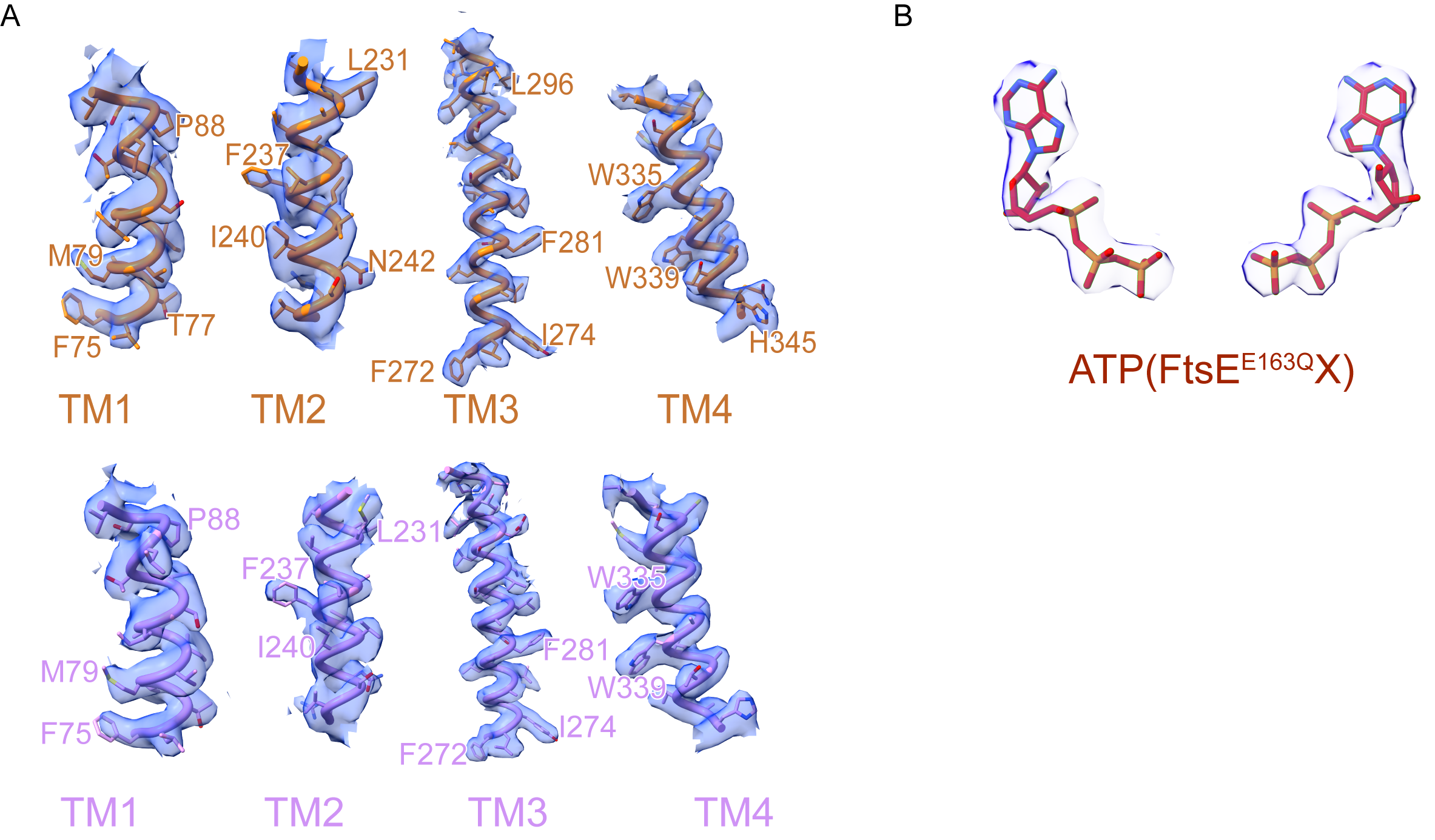

Supplement: S3 Fig — (A) The map of transmembrane helices. (B) The map of ATP. (TIF) [file pbio.3002628.s003.tif]

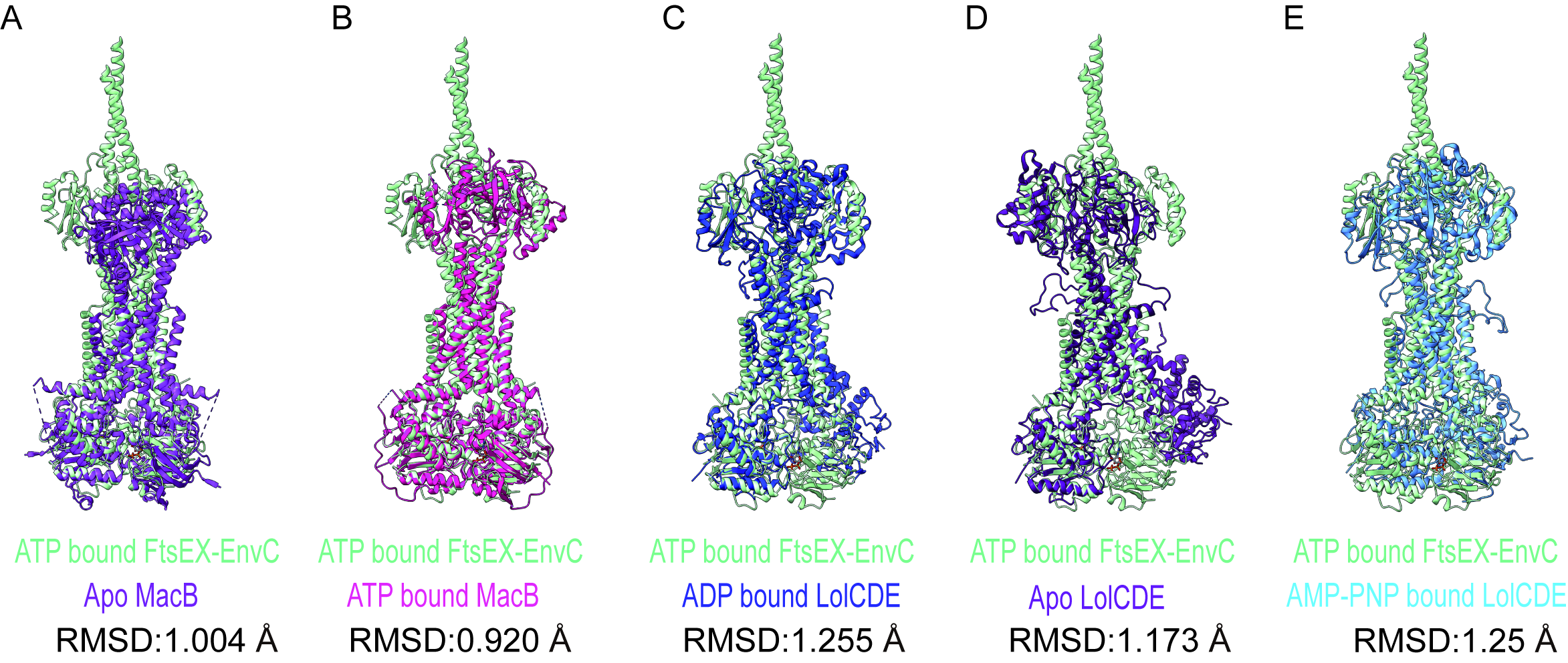

Supplement: S4 Fig — (A) ATP-bound FtsEE163QX-EnvC (cyan) is superimposed to apo MacB (bright purple) (PDB ID:5GKO). (B) ATP bound FtsEE163QX-EnvC (cyan) is superimposed to ATP-bound MacB (fuchsia) (PDB ID:5LJ7). (C) ATP-bound FtsEE163QX-EnvC (cyan) is superimposed to ADP-bound LolCDE (dark blue) (PDB ID:7ARL). (D) ATP-bound FtsEE163QX-EnvC (cyan) is superimposed to apo LolCDE (dark purple) (PDB ID:7ARI). (E) ATP-bound FtsEE163QX-EnvC (cyan) is superimposed to AMP-PNP-bound LolCDE (light blue) (PDB ID:7ARK). (TIF) [file pbio.3002628.s004.tif]

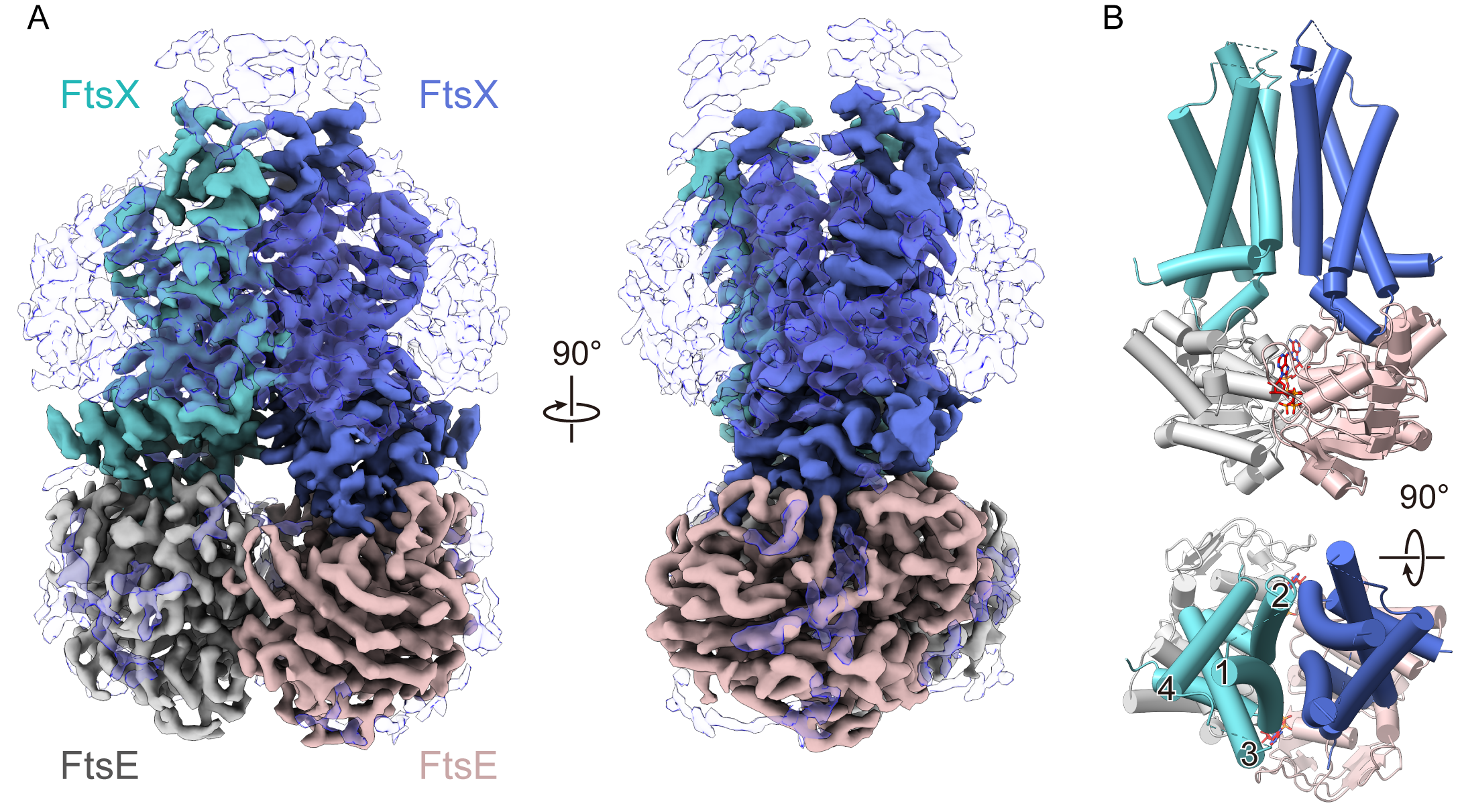

Supplement: S5 Fig — (A) Cryo-EM map of FtsEE163QX complex, viewed from front and side. (B) A cartoon representation of FtsEE163QX complex in front and top. Transmembrane helices 1, 2, 3, and 4 are labeled out. (TIF) [file pbio.3002628.s005.tif]

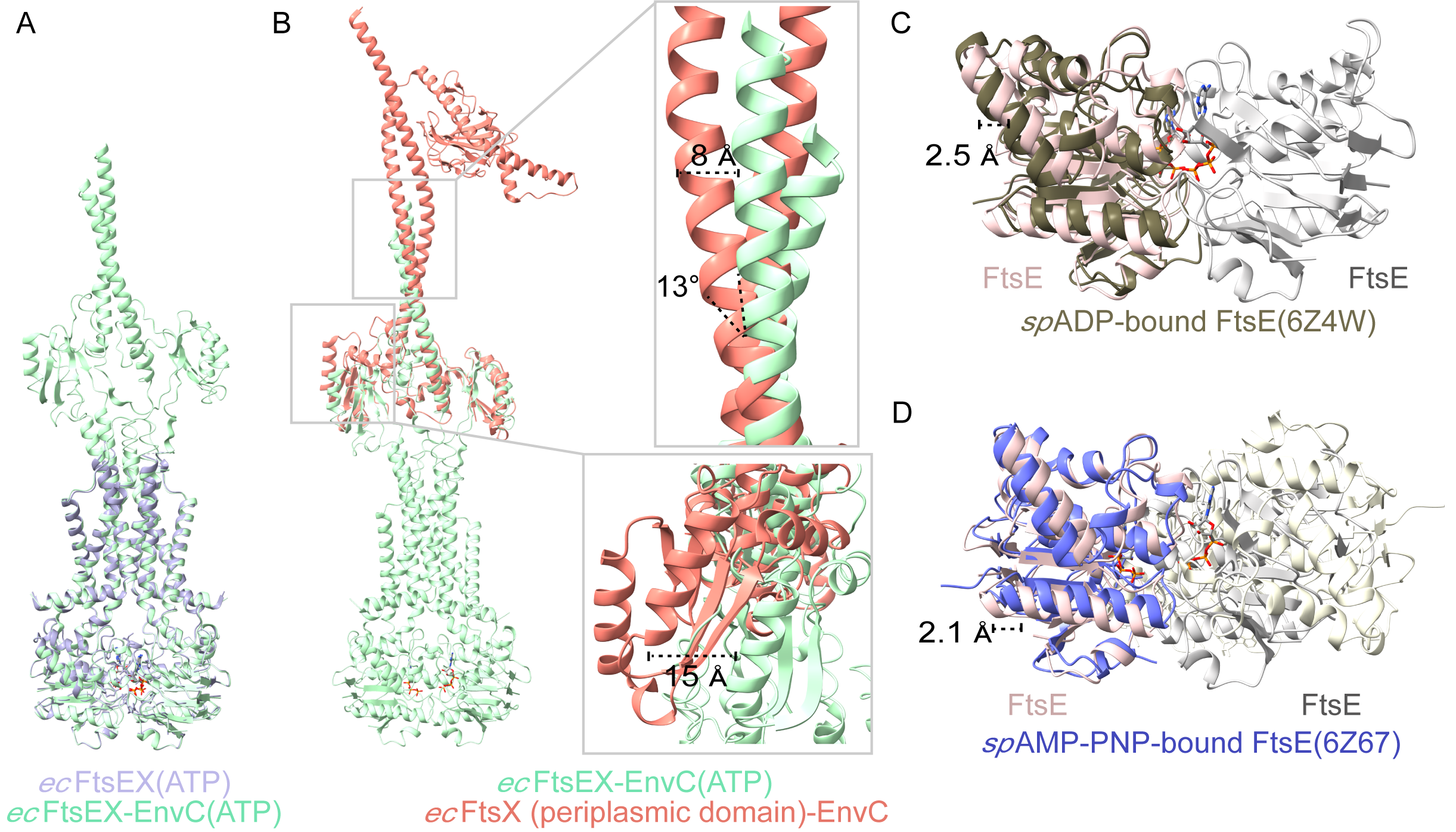

Supplement: S6 Fig — (A) ecATP-bound FtsEE163QX-EnvC is superimposed to ecATP-bound FtsEX. (B) ecATP-bound FtsEE163QX-EnvC is superimposed to ecFtsX (periplamic domain)-EnvC. (C) ecATP-bound FtsEE163QX-EnvC is superimposed to spADP-bound FtsE(6Z4W). (D) ecATP-bound FtsEE163QX-EnvC is superimposed to spAMP-PNP-bound FtsE(6Z67). (TIF) [file pbio.3002628.s006.tif]

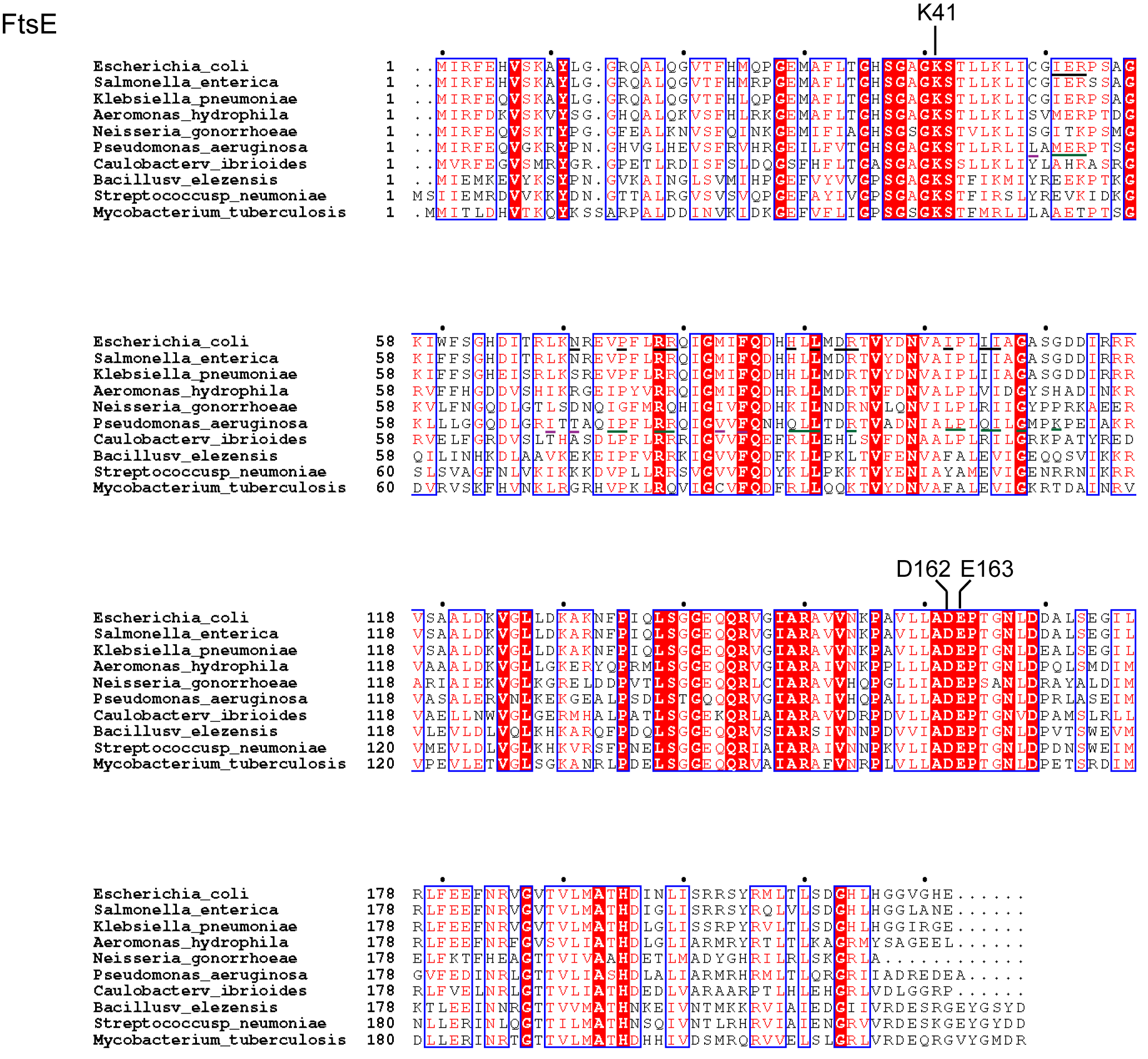

Supplement: S7 Fig — The resides of K41, D162, and E163 which are related to ATPase is highly conserved over different bacterial species. (TIF) [file pbio.3002628.s007.tif]

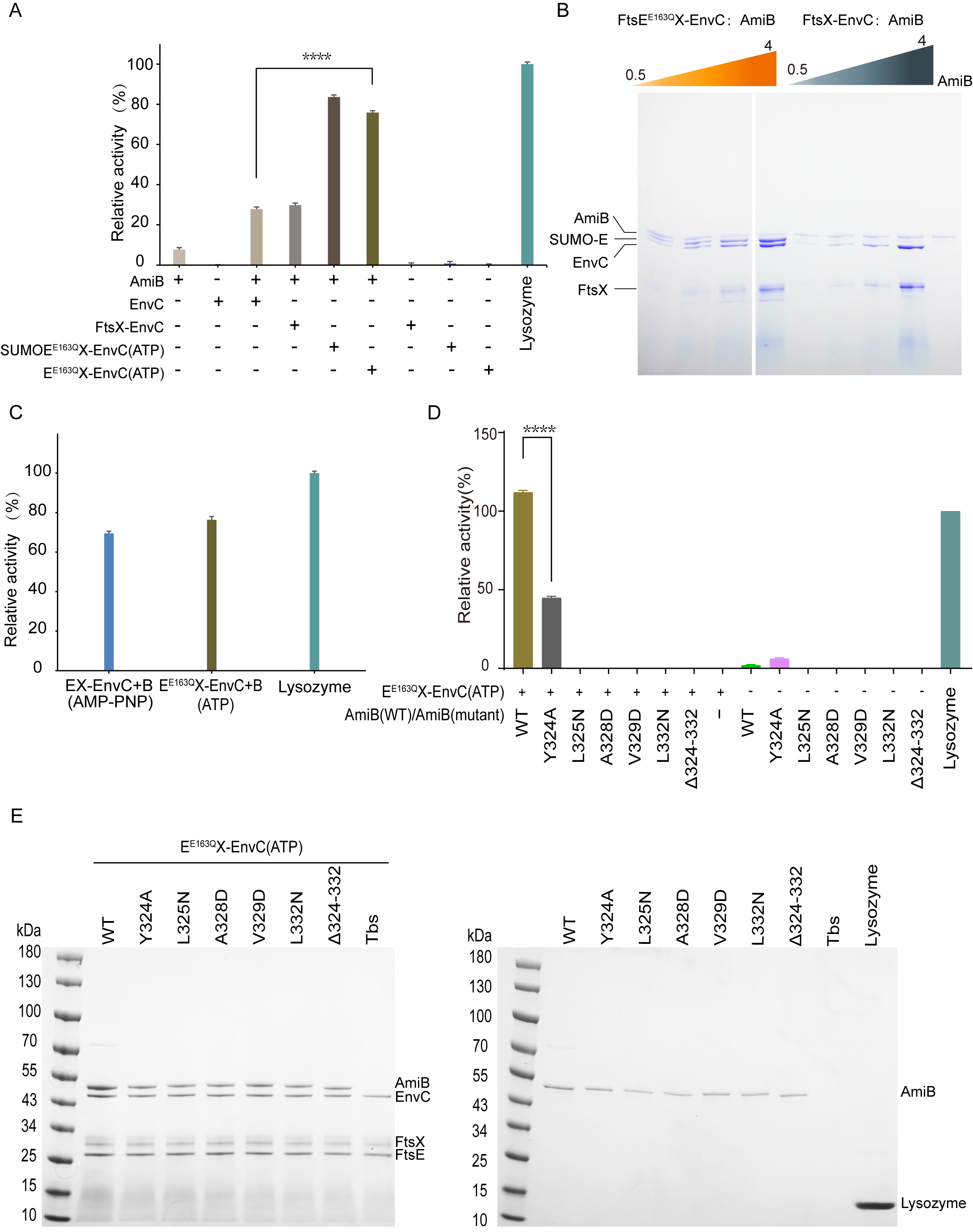

Supplement: S8 Fig — (A) PG degradation assay representing amidase activity. The graph shows the degradation of FITC-labeled sacculi by AmiB and/or regulators. Lysozyme was used as positive control and TBS as negative control. Individual AmiB showed a slight activity. EnvC together with AmiB resulted in an increase in PG degradation. FtsEE163QX-EnvC boosted the activity of AmiB to degrade PG compared to FtsX-EnvC. Four asterisks signify a P-value of less than 0.0001. (B) SDS-PAGE analysis of samples involved in the reaction of Fig 3B. (C) PG degradation assay of FtsEX and FtsEE163QX. (D) PG degradation assays of AmiB and AmiB mutants. Four asterisks signify a P-value of less than 0.0001. (E) SDS-PAGE analysis of samples involved in the reaction of S8D Fig. The data underlying the graphs shown in the figure can be found in S2 Data and S1 Raw Images. (TIF) [file pbio.3002628.s008.tif]

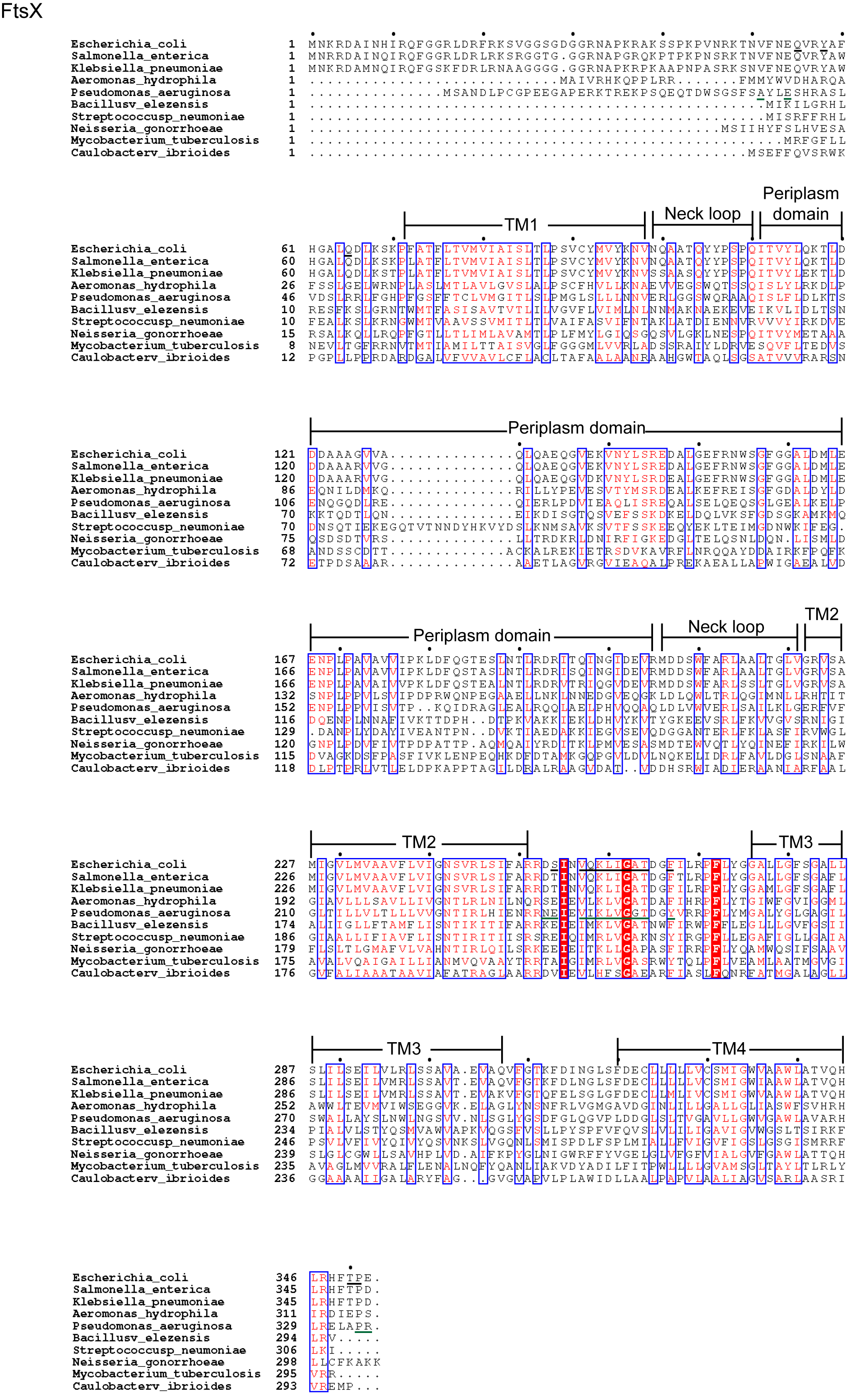

Supplement: S9 Fig — (TIF) [file pbio.3002628.s009.tif]

Figure 4B

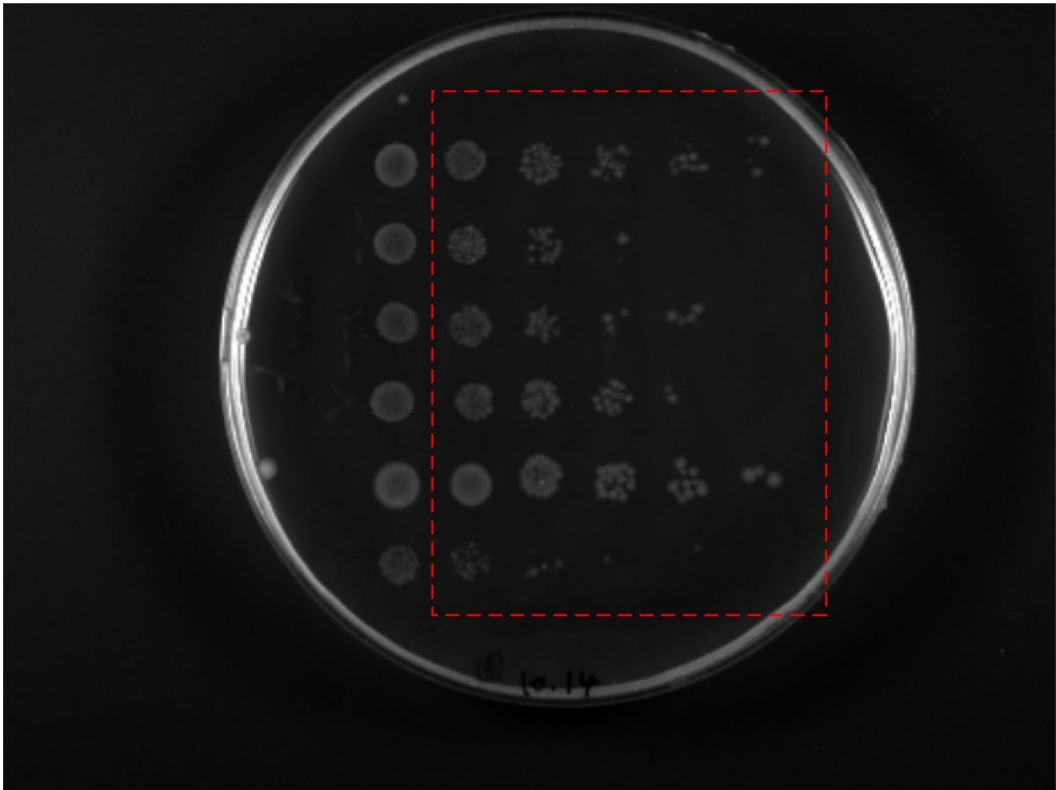

Figure 4C

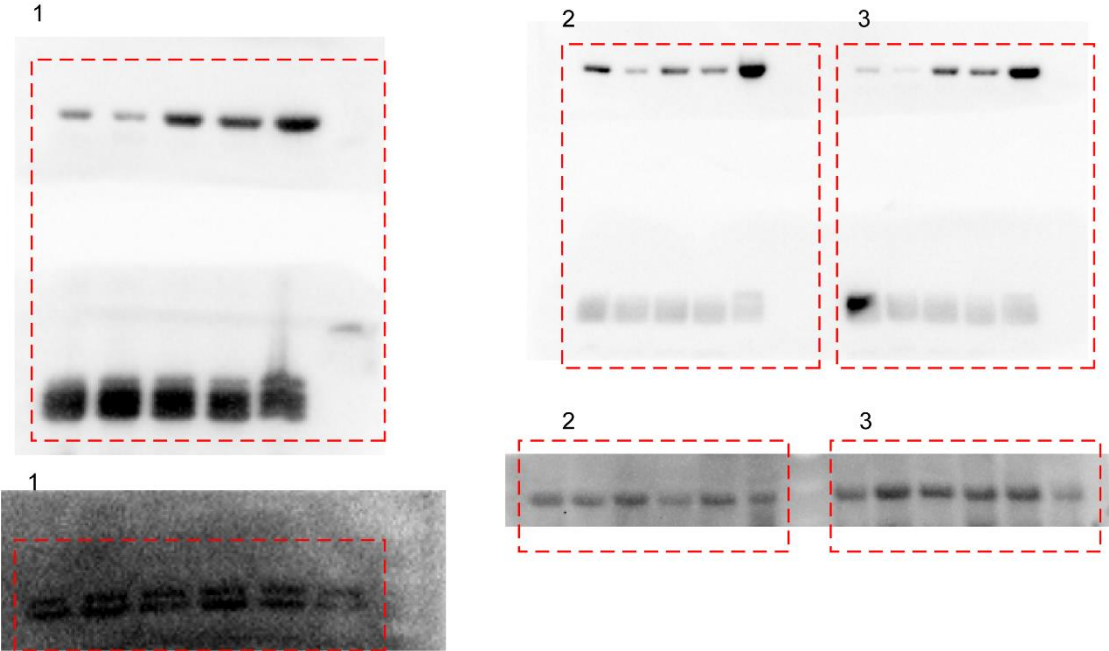

Figure S1A

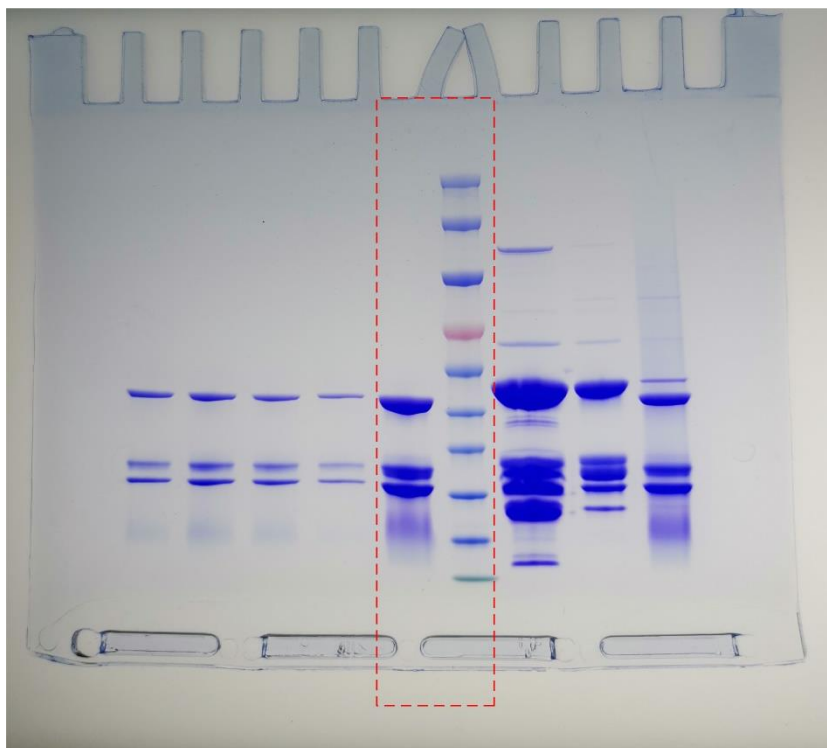

Figure S8B

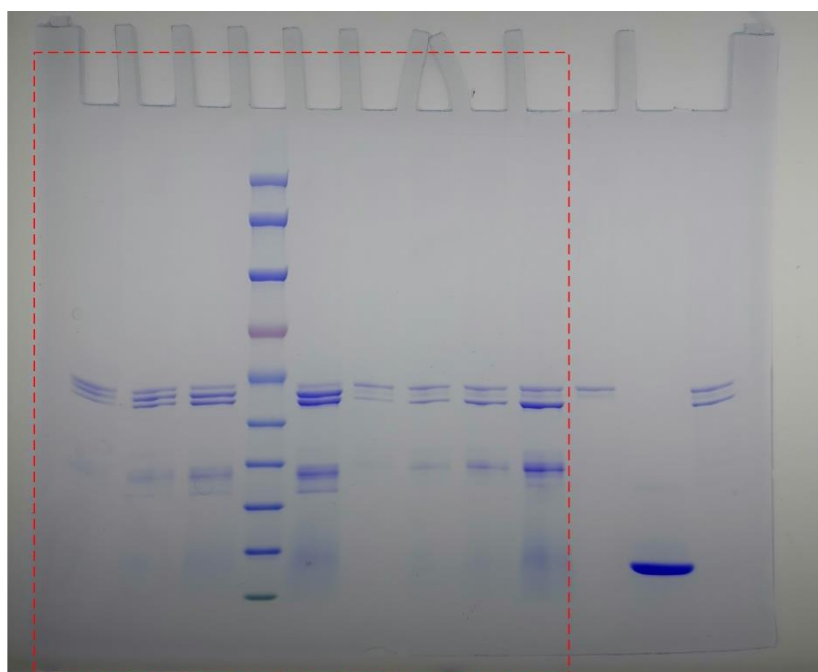

Figure S8E

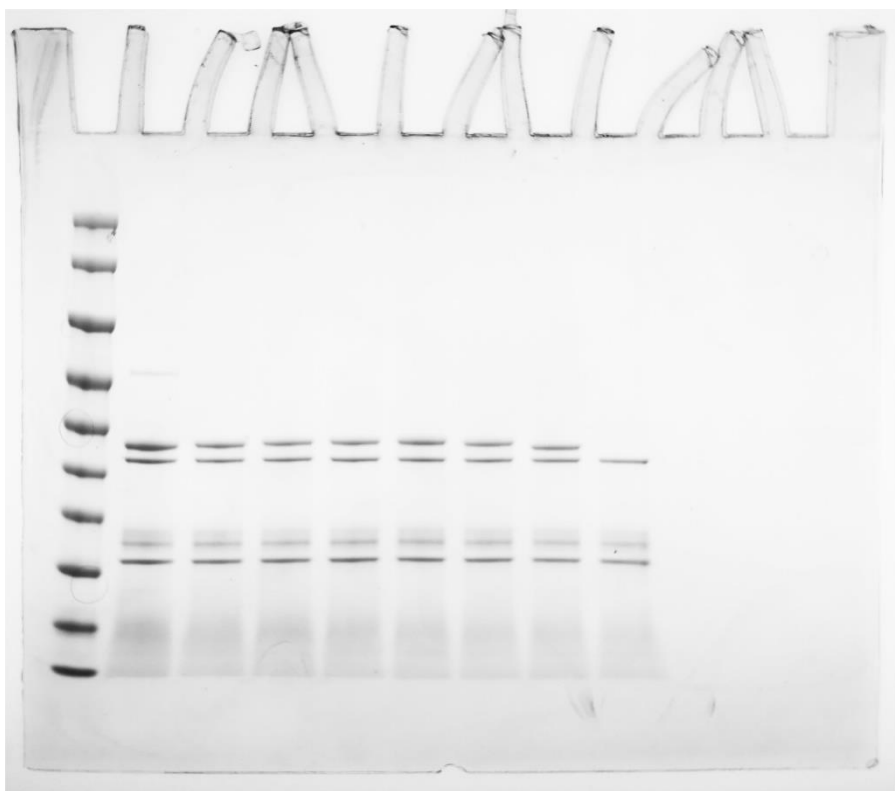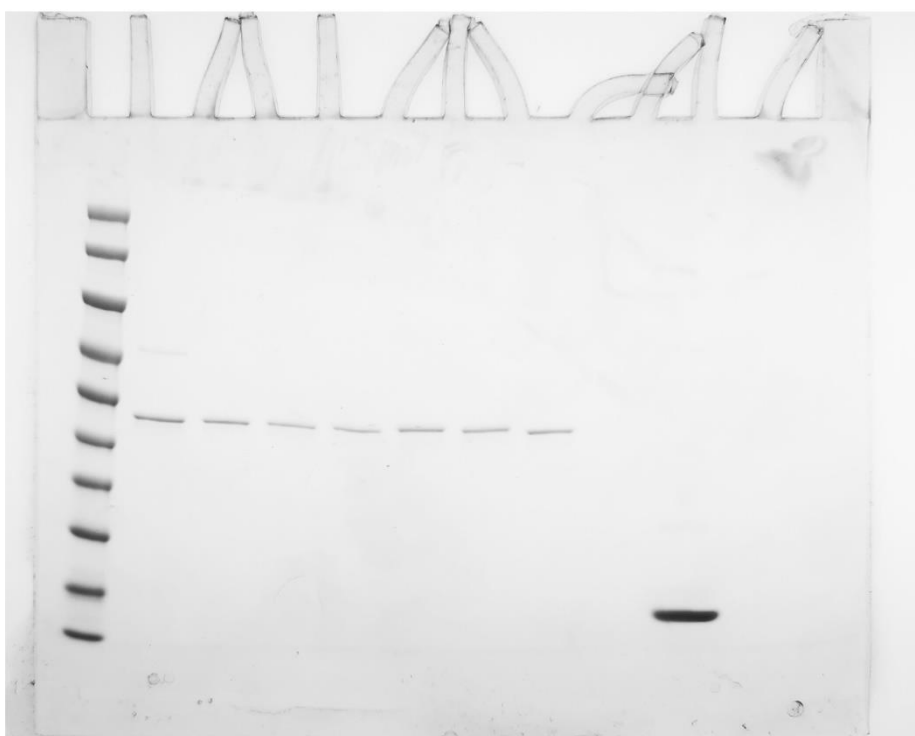

Supplement: S1 Raw Images — (PDF) [file pbio.3002628.s013.pdf]
