# Supplementary material for: Structure and activity of the septal peptidoglycan hydrolysis machinery crucial for bacterial cell division
Source: PLoS Biol. 2024 May 30;22(5):e3002628. doi: 10.1371/journal.pbio.3002628 (PMC11139282; doi:10.1371/journal.pbio.3002628)
Supplement: S1 Data — (PDF) [file pbio.3002628.s011.pdf]

Full wwPDB EM Validation Report (i)

Feb 21, 2024 – 02:01 AM JST

PDB ID	:	8X61
EMDB ID	:	EMD-38077
Title	:	Cryo-EM structure of ATP-bound FtsE(E163Q)X
Deposited on	:	2023-11-20
Resolution	:	3.05 Å(reported)

This wwPDB validation report is for manuscript review

This is a Full wwPDB EM Validation Report.

This report is produced by the wwPDB biocuration pipeline after annotation of the structure.

We welcome your comments at *validation@mail.wwpdb.org*
A user guide is available at
https://www.wwpdb.org/validation/2017/EMValidationReportHelp
with specific help available everywhere you see the (i) symbol.

The types of validation reports are described at
http://www.wwpdb.org/validation/2017/FAQs#types.

The following versions of software and data (see references (i)) were used in the production of this report:

EMDB validation analysis	:	0.0.1.dev70
Mogul	:	1.8.5 (274361), CSD as541be (2020)
MolProbity	:	4.02b-467
buster-report	:	1.1.7 (2018)
Percentile statistics	:	20191225.v01 (using entries in the PDB archive December 25th 2019)
MapQ	:	1.9.13
Ideal geometry (proteins)	:	Engh & Huber (2001)
Ideal geometry (DNA, RNA)	:	Parkinson et al. (1996)
Validation Pipeline (wwPDB-VP)	:	2.36

For Manuscript Review

1 Overall quality at a glance (i)

The following experimental techniques were used to determine the structure:
ELECTRON MICROSCOPY

The reported resolution of this entry is 3.05 Å.

Percentile scores (ranging between 0-100) for global validation metrics of the entry are shown in the following graphic. The table shows the number of entries on which the scores are based.

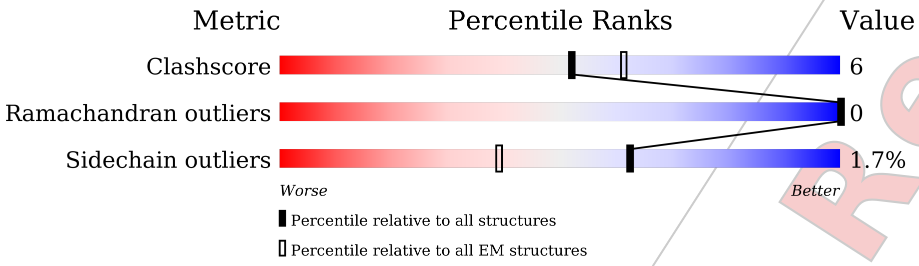

Metric	Whole archive (#Entries)	EM structures (#Entries)
Clashscore	158937	4297
Ramachandran outliers	154571	4023
Sidechain outliers	154315	3826

The table below summarises the geometric issues observed across the polymeric chains and their fit to the map. The red, orange, yellow and green segments of the bar indicate the fraction of residues that contain outliers for >=3, 2, 1 and 0 types of geometric quality criteria respectively. A grey segment represents the fraction of residues that are not modelled. The numeric value for each fraction is indicated below the corresponding segment, with a dot representing fractions <=5% The upper red bar (where present) indicates the fraction of residues that have poor fit to the EM map (all-atom inclusion < 40%). The numeric value is given above the bar.

Mol	Chain	Length	Quality of chain
1	A	222	85% 12% •
1	B	222	85% 12% •
2	C	352	• 40% 6% 54%
2	D	352	37% 9% • 54%

For Manuscript Review

2 Entry composition (i)

There are 3 unique types of molecules in this entry. The entry contains 5737 atoms, of which 0 are hydrogens and 0 are deuteriums.

In the tables below, the AltConf column contains the number of residues with at least one atom in alternate conformation and the Trace column contains the number of residues modelled with at most 2 atoms.

- Molecule 1 is a protein called Cell division ATP-binding protein FtsE.

Mol	Chain	Residues	Atoms					AltConf	Trace
			Total	C	N	O	S		
1	A	216	1680	1060	314	298	8	0	0
1	B	216	1662	1050	307	297	8	0	0

There are 2 discrepancies between the modelled and reference sequences:

Chain	Residue	Modelled	Actual	Comment	Reference
A	163	GLN	GLU	engineered mutation	UNP P0A9R7
B	163	GLN	GLU	engineered mutation	UNP P0A9R7

- Molecule 2 is a protein called Cell division protein FtsX.

Mol	Chain	Residues	Atoms					AltConf	Trace
			Total	C	N	O	S		
2	C	161	1141	757	186	190	8	0	0
2	D	163	1192	792	192	200	8	0	0

- Molecule 3 is ADENOSINE-5'-TRIPHOSPHATE (three-letter code: ATP) (formula: $C_{10}H_{16}N_5O_{13}P_3$) (labeled as "Ligand of Interest" by depositor).

For Manuscript Review

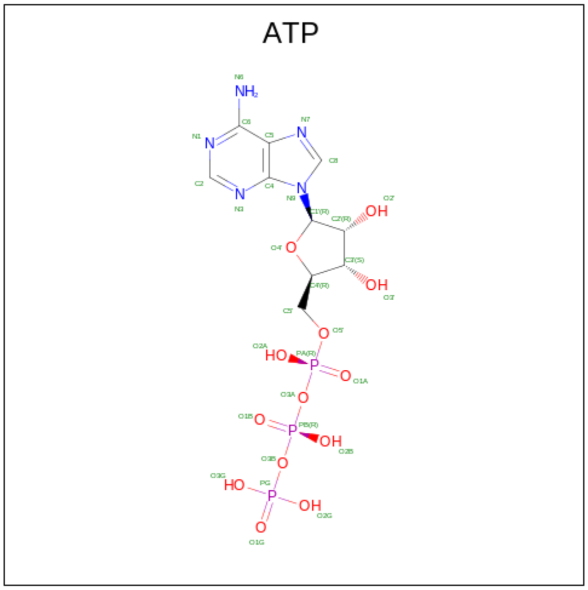

Mol	Chain	Residues	Atoms					AltConf
			Total	C	N	O	P	
3	A	1	31	10	5	13	3	0
3	B	1	31	10	5	13	3	0

For Manuscript Review

3 Residue-property plots (i)

These plots are drawn for all protein, RNA, DNA and oligosaccharide chains in the entry. The first graphic for a chain summarises the proportions of the various outlier classes displayed in the second graphic. The second graphic shows the sequence view annotated by issues in geometry and atom inclusion in map density. Residues are color-coded according to the number of geometric quality criteria for which they contain at least one outlier: green = 0, yellow = 1, orange = 2 and red = 3 or more. A red diamond above a residue indicates a poor fit to the EM map for this residue (all-atom inclusion < 40%). Stretches of 2 or more consecutive residues without any outlier are shown as a green connector. Residues present in the sample, but not in the model, are shown in grey.

- Molecule 1: Cell division ATP-binding protein FtsE

Chain A:

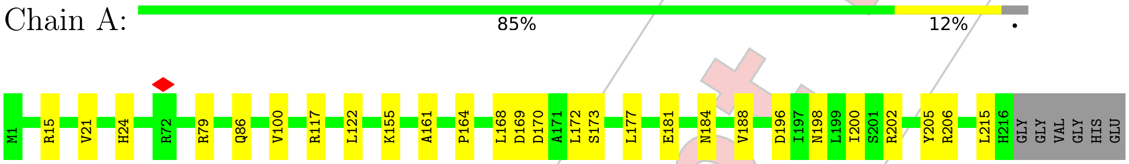

- Molecule 1: Cell division ATP-binding protein FtsE

Chain B:

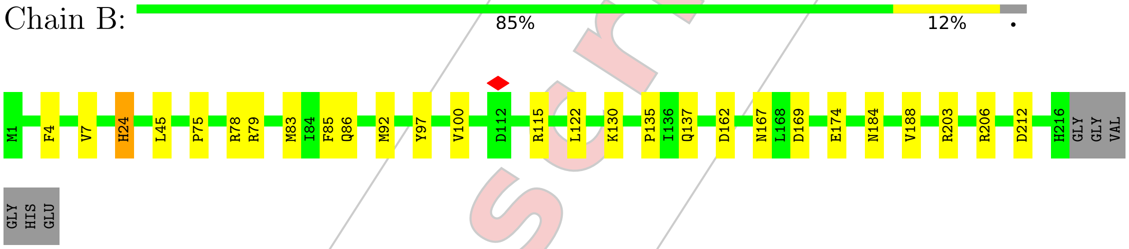

- Molecule 2: Cell division protein FtsX

Chain C:

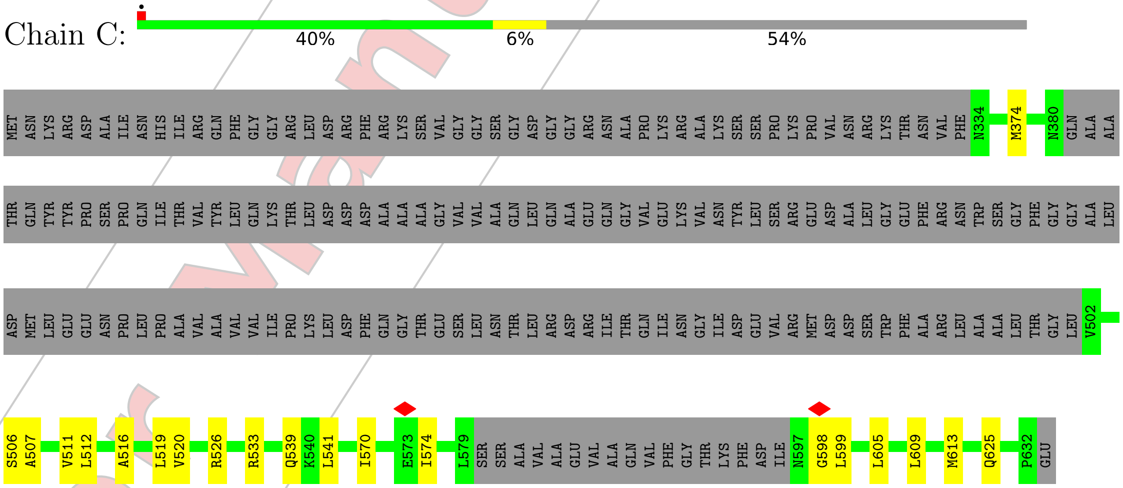

- Molecule 2: Cell division protein FtsX

Chain D:

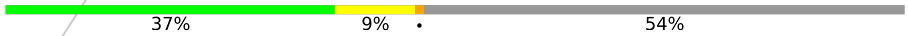

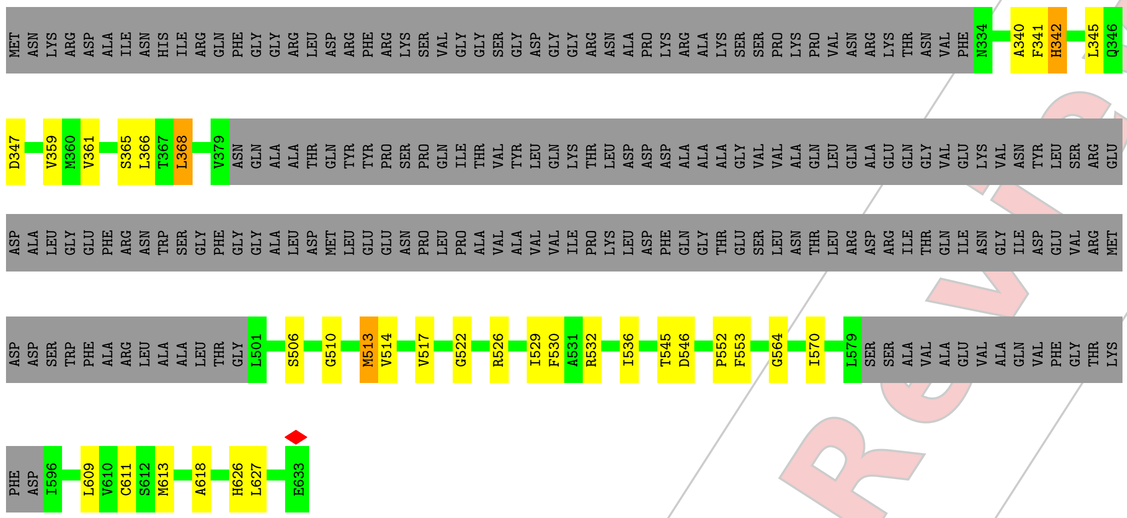

For Manuscript Review

4 Experimental information (i)

Property	Value	Source
EM reconstruction method	SINGLE PARTICLE	Depositor
Imposed symmetry	POINT, Not provided	
Number of particles used	370231	Depositor
Resolution determination method	FSC 0.143 CUT-OFF	Depositor
CTF correction method	PHASE FLIPPING AND AMPLITUDE CORRECTION	Depositor
Microscope	FEI TITAN	Depositor
Voltage (kV)	300	Depositor
Electron dose ($e^-/\mathring{A}^2$)	60.0	Depositor
Minimum defocus (nm)	1200	Depositor
Maximum defocus (nm)	2500	Depositor
Magnification	Not provided	
Image detector	GATAN K3 BIOQUANTUM (6k x 4k)	Depositor
Maximum map value	2.297	Depositor
Minimum map value	-1.452	Depositor
Average map value	-0.000	Depositor
Map value standard deviation	0.023	Depositor
Recommended contour level	0.1	Depositor
Map size (Å)	302.4, 302.4, 302.4	wwPDB
Map dimensions	360, 360, 360	wwPDB
Map angles (°)	90.0, 90.0, 90.0	wwPDB
Pixel spacing (Å)	0.84, 0.84, 0.84	Depositor

For Manuscript Review

5 Model quality (i)

5.1 Standard geometry (i)

Bond lengths and bond angles in the following residue types are not validated in this section: ATP

The Z score for a bond length (or angle) is the number of standard deviations the observed value is removed from the expected value. A bond length (or angle) with $|Z| > 5$ is considered an outlier worth inspection. RMSZ is the root-mean-square of all Z scores of the bond lengths (or angles).

Mol	Chain	Bond lengths		Bond angles	
		RMSZ	#$\|Z\|$ >5	RMSZ	#$\|Z\|$ >5
1	A	0.26	0/1709	0.52	0/2302
1	B	0.26	0/1691	0.52	0/2280
2	C	0.25	0/1160	0.43	0/1582
2	D	0.25	0/1213	0.43	0/1652
All	All	0.26	0/5773	0.49	0/7816

There are no bond length outliers.

There are no bond angle outliers.

There are no chirality outliers.

There are no planarity outliers.

5.2 Too-close contacts (i)

In the following table, the Non-H and H(model) columns list the number of non-hydrogen atoms and hydrogen atoms in the chain respectively. The H(added) column lists the number of hydrogen atoms added and optimized by MolProbity. The Clashes column lists the number of clashes within the asymmetric unit, whereas Symm-Clashes lists symmetry-related clashes.

Mol	Chain	Non-H	H(model)	H(added)	Clashes	Symm-Clashes
1	A	1680	0	1724	21	0
1	B	1662	0	1689	17	0
2	C	1141	0	1142	15	0
2	D	1192	0	1217	22	0
3	A	31	0	12	0	0
3	B	31	0	12	0	0
All	All	5737	0	5796	66	0

The all-atom clashscore is defined as the number of clashes found per 1000 atoms (including hydrogen atoms). The all-atom clashscore for this structure is 6.

For Manuscript Review

All (66) close contacts within the same asymmetric unit are listed below, sorted by their clash magnitude.

Atom-1	Atom-2	Interatomic distance (Å)	Clash overlap (Å)
2:C:605:LEU:O	2:C:609:LEU:HD23	1.75	0.85
2:C:570:ILE:O	2:C:574:ILE:HG12	1.75	0.84
2:C:609:LEU:O	2:C:613:MET:HG2	1.84	0.77
2:C:512:LEU:HD23	2:D:368:LEU:HG	1.70	0.73
2:C:519:LEU:HB3	2:D:361:VAL:HG22	1.71	0.72
1:A:184:ASN:ND2	1:A:188:VAL:O	2.23	0.70
2:D:526:ARG:O	2:D:526:ARG:HD2	1.92	0.69
2:C:374:MET:HE3	2:C:599:LEU:H	1.56	0.69
1:A:86:GLN:NE2	1:B:167:ASN:OD1	2.23	0.68
1:A:100:VAL:HG21	1:A:122:LEU:HD11	1.77	0.66
1:A:15:ARG:HH12	1:B:130:LYS:HD3	1.60	0.66
1:A:198:ASN:O	1:A:202:ARG:NH1	2.30	0.65
2:C:516:ALA:O	2:C:520:VAL:HG23	1.96	0.65
2:D:340:ALA:HB1	2:D:552:PRO:HD3	1.80	0.64
2:D:340:ALA:CB	2:D:552:PRO:HD3	2.30	0.62
2:C:374:MET:CE	2:C:599:LEU:H	2.14	0.60
2:C:374:MET:HG2	2:C:598:GLY:HA2	1.82	0.60
2:D:536:ILE:HD11	2:D:553:PHE:HE2	1.68	0.58
1:B:100:VAL:HG21	1:B:122:LEU:HD21	1.86	0.58
2:C:526:ARG:NH2	2:C:625:GLN:OE1	2.36	0.58
2:D:530:PHE:HD1	2:D:626:HIS:HE2	1.51	0.57
1:A:21:VAL:HG13	1:A:215:LEU:HD22	1.87	0.57
1:B:174:GLU:OE1	1:B:203:ARG:NH1	2.35	0.56
2:D:526:ARG:HD2	2:D:526:ARG:C	2.25	0.56
2:D:347:ASP:OD2	2:D:532:ARG:NH1	2.40	0.55
2:C:374:MET:HE2	2:C:598:GLY:HA3	1.89	0.55
2:C:533:ARG:HG2	2:C:533:ARG:HH11	1.70	0.55
2:D:510:GLY:HA2	2:D:513:MET:SD	2.46	0.55
1:A:24:HIS:O	1:A:206:ARG:NH1	2.32	0.54
1:B:169:ASP:N	1:B:169:ASP:OD1	2.42	0.51
2:D:609:LEU:O	2:D:613:MET:HG3	2.11	0.51
1:B:92:MET:SD	1:B:135:PRO:HB2	2.52	0.50
1:B:75:PRO:O	1:B:79:ARG:HG3	2.11	0.50
2:D:342:HIS:O	2:D:342:HIS:ND1	2.45	0.49
1:A:181:GLU:OE2	1:A:205:TYR:OH	2.24	0.49
1:A:169:ASP:N	1:A:169:ASP:OD1	2.44	0.49
1:B:184:ASN:ND2	1:B:188:VAL:O	2.32	0.48
1:A:161:ALA:HB1	1:A:164:PRO:HG3	1.94	0.48
1:B:97:TYR:OH	1:B:115:ARG:HA	2.13	0.48
1:A:117:ARG:NH1	1:A:155:LYS:HE2	2.28	0.48

Continued on next page...

For Manuscript Review

Continued from previous page...

Atom-1	Atom-2	Interatomic distance (Å)	Clash overlap (Å)
1:B:4:PHE:HD2	1:B:7:VAL:HG11	1.79	0.47
1:A:15:ARG:NH2	1:B:137:GLN:O	2.48	0.47
1:B:86:GLN:HB2	1:B:162:ASP:OD2	2.15	0.47
1:B:78:ARG:HH11	2:C:541:LEU:HA	1.79	0.47
1:A:15:ARG:NH1	1:B:130:LYS:HD3	2.29	0.46
1:A:79:ARG:HH22	2:D:545:THR:HG23	1.80	0.46
2:C:507:ALA:O	2:C:511:VAL:HG13	2.14	0.46
1:B:45:LEU:HD12	1:B:162:ASP:HB2	1.97	0.46
2:D:529:ILE:HG12	2:D:553:PHE:CD2	2.51	0.46
1:A:169:ASP:O	1:A:173:SER:OG	2.22	0.45
1:A:117:ARG:HH12	1:A:155:LYS:HE2	1.82	0.45
1:A:79:ARG:NH2	2:D:545:THR:HG23	2.31	0.45
1:B:24:HIS:O	1:B:206:ARG:NH1	2.36	0.45
2:D:366:LEU:HD11	2:D:611:CYS:SG	2.57	0.45
1:A:169:ASP:OD1	1:A:172:LEU:HB2	2.17	0.45
1:A:177:LEU:O	1:A:181:GLU:HG3	2.17	0.44
2:D:546:ASP:OD1	2:D:627:LEU:HD21	2.17	0.44
2:D:570:ILE:HD13	2:D:570:ILE:N	2.33	0.43
2:D:341:PHE:CE2	2:D:345:LEU:HD11	2.53	0.43
2:D:514:VAL:O	2:D:517:VAL:HG12	2.19	0.43
1:B:83:MET:HG2	1:B:85:PHE:CE1	2.54	0.42
1:A:168:LEU:HD22	1:A:172:LEU:HD13	2.01	0.42
1:A:196:ASP:O	1:A:200:ILE:HG23	2.19	0.42
2:D:522:GLY:HA2	2:D:618:ALA:HB1	2.01	0.42
2:D:359:VAL:HG13	2:D:564:GLY:HA2	2.03	0.41
2:C:574:ILE:HG12	2:C:574:ILE:H	1.63	0.40

There are no symmetry-related clashes.

5.3 Torsion angles (i)

5.3.1 Protein backbone (i)

In the following table, the Percentiles column shows the percent Ramachandran outliers of the chain as a percentile score with respect to all PDB entries followed by that with respect to all EM entries.

The Analysed column shows the number of residues for which the backbone conformation was analysed, and the total number of residues.

For Manuscript Review

Mol	Chain	Analysed	Favoured	Allowed	Outliers	Percentiles	
1	A	214/222 (96%)	205 (96%)	9 (4%)	0	100	100
1	B	214/222 (96%)	204 (95%)	10 (5%)	0	100	100
2	C	155/352 (44%)	154 (99%)	1 (1%)	0	100	100
2	D	157/352 (45%)	153 (98%)	4 (2%)	0	100	100
All	All	740/1148 (64%)	716 (97%)	24 (3%)	0	100	100

There are no Ramachandran outliers to report.

5.3.2 Protein sidechains ⓘ

In the following table, the Percentiles column shows the percent sidechain outliers of the chain as a percentile score with respect to all PDB entries followed by that with respect to all EM entries.

The Analysed column shows the number of residues for which the sidechain conformation was analysed, and the total number of residues.

Mol	Chain	Analysed	Rotameric	Outliers	Percentiles	
1	A	178/181 (98%)	177 (99%)	1 (1%)	86	93
1	B	174/181 (96%)	172 (99%)	2 (1%)	73	88
2	C	110/289 (38%)	108 (98%)	2 (2%)	59	80
2	D	122/289 (42%)	117 (96%)	5 (4%)	30	61
All	All	584/940 (62%)	574 (98%)	10 (2%)	62	82

All (10) residues with a non-rotameric sidechain are listed below:

Mol	Chain	Res	Type
1	A	170	ASP
1	B	24	HIS
1	B	212	ASP
2	C	506	SER
2	C	539	GLN
2	D	342	HIS
2	D	365	SER
2	D	368	LEU
2	D	506	SER
2	D	513	MET

Sometimes sidechains can be flipped to improve hydrogen bonding and reduce clashes. All (1) such sidechains are listed below:

For Manuscript Review

Mol	Chain	Res	Type
1	B	19	GLN

5.3.3 RNA (i)

There are no RNA molecules in this entry.

5.4 Non-standard residues in protein, DNA, RNA chains (i)

There are no non-standard protein/DNA/RNA residues in this entry.

5.5 Carbohydrates (i)

There are no monosaccharides in this entry.

5.6 Ligand geometry (i)

2 ligands are modelled in this entry.

In the following table, the Counts columns list the number of bonds (or angles) for which Mogul statistics could be retrieved, the number of bonds (or angles) that are observed in the model and the number of bonds (or angles) that are defined in the Chemical Component Dictionary. The Link column lists molecule types, if any, to which the group is linked. The Z score for a bond length (or angle) is the number of standard deviations the observed value is removed from the expected value. A bond length (or angle) with $|Z| > 2$ is considered an outlier worth inspection. RMSZ is the root-mean-square of all Z scores of the bond lengths (or angles).

Mol	Type	Chain	Res	Link	Bond lengths			Bond angles		
					Counts	RMSZ	$\#\|Z\| > 2$	Counts	RMSZ	$\#\|Z\| > 2$
3	ATP	B	301	-	26,33,33	0.69	0	31,52,52	0.80	1 (3%)
3	ATP	A	301	-	26,33,33	0.70	0	31,52,52	0.80	1 (3%)

In the following table, the Chirals column lists the number of chiral outliers, the number of chiral centers analysed, the number of these observed in the model and the number defined in the Chemical Component Dictionary. Similar counts are reported in the Torsion and Rings columns. '-' means no outliers of that kind were identified.

Mol	Type	Chain	Res	Link	Chirals	Torsions	Rings
3	ATP	B	301	-	-	0/18/38/38	0/3/3/3
3	ATP	A	301	-	-	0/18/38/38	0/3/3/3

There are no bond length outliers.

For Manuscript Review

All (2) bond angle outliers are listed below:

Mol	Chain	Res	Type	Atoms	Z	Observed(°)	Ideal(°)
3	B	301	ATP	C5-C6-N6	2.26	123.78	120.35
3	A	301	ATP	C5-C6-N6	2.25	123.78	120.35

There are no chirality outliers.

There are no torsion outliers.

There are no ring outliers.

No monomer is involved in short contacts.

The following is a two-dimensional graphical depiction of Mogul quality analysis of bond lengths, bond angles, torsion angles, and ring geometry for all instances of the Ligand of Interest. In addition, ligands with molecular weight > 250 and outliers as shown on the validation Tables will also be included. For torsion angles, if less then 5% of the Mogul distribution of torsion angles is within 10 degrees of the torsion angle in question, then that torsion angle is considered an outlier. Any bond that is central to one or more torsion angles identified as an outlier by Mogul will be highlighted in the graph. For rings, the root-mean-square deviation (RMSD) between the ring in question and similar rings identified by Mogul is calculated over all ring torsion angles. If the average RMSD is greater than 60 degrees and the minimal RMSD between the ring in question and any Mogul-identified rings is also greater than 60 degrees, then that ring is considered an outlier. The outliers are highlighted in purple. The color gray indicates Mogul did not find sufficient equivalents in the CSD to analyse the geometry.

For Manuscript Review

Ligand ATP B 301

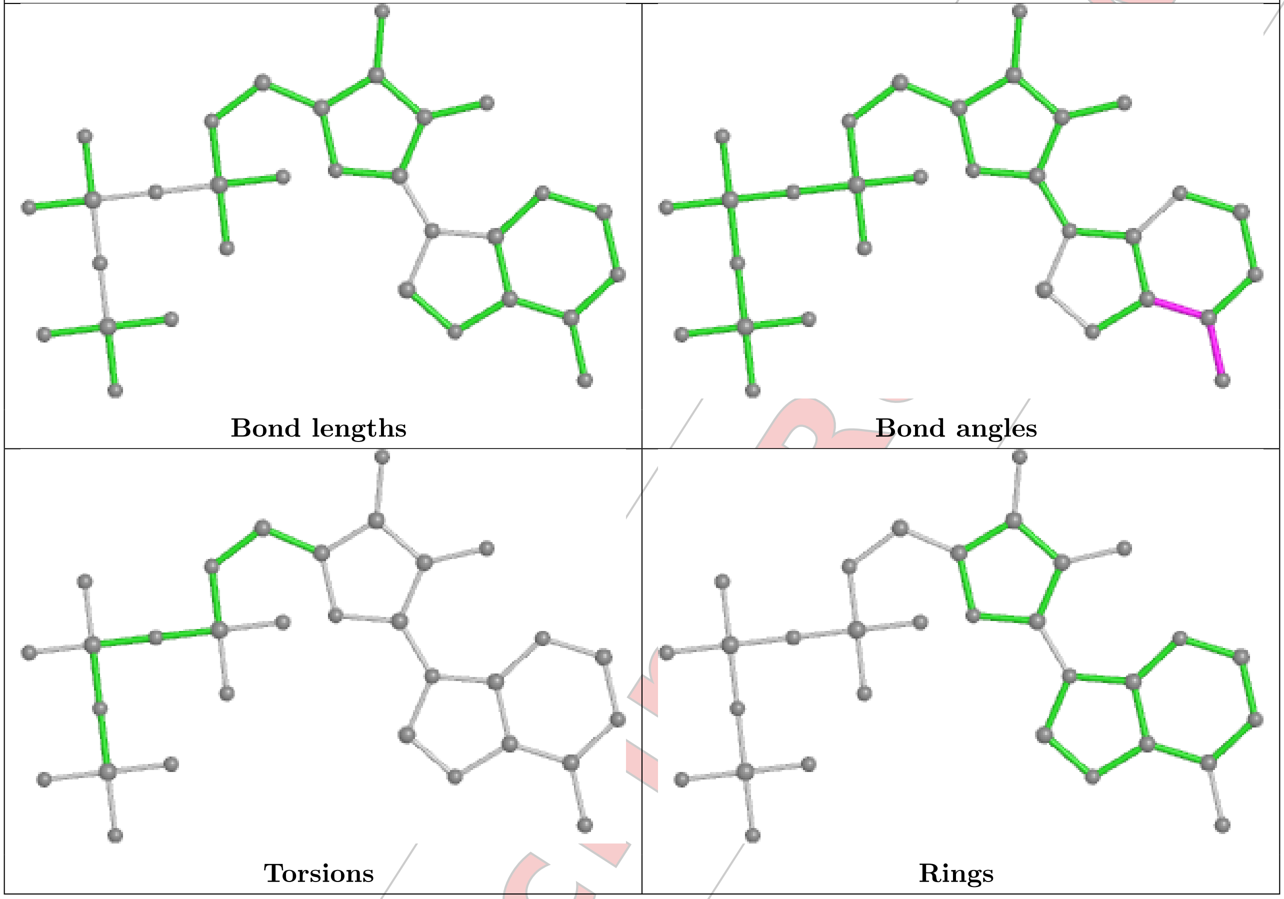

For Manuscript Review

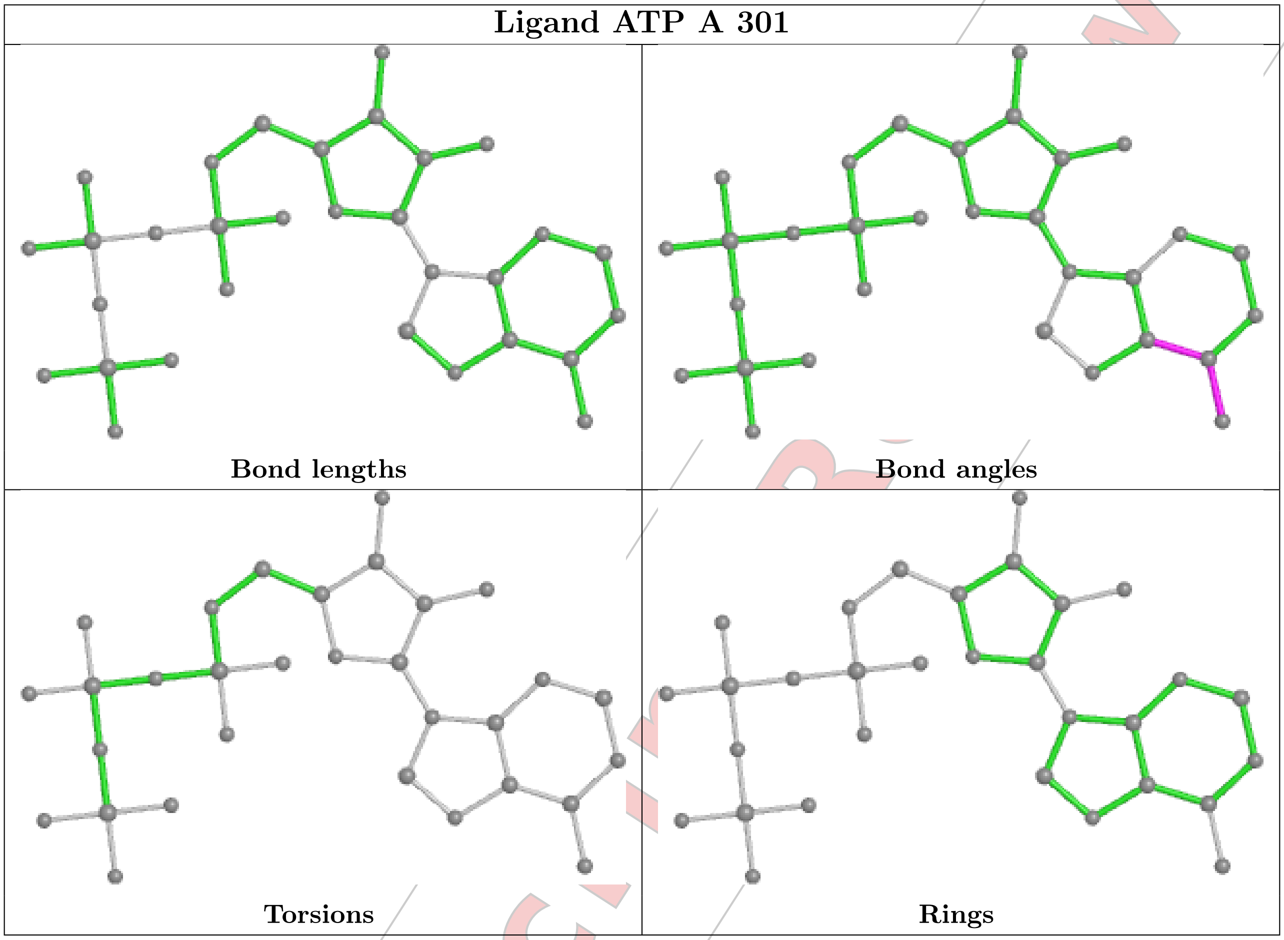

5.7 Other polymers (i)

There are no such residues in this entry.

5.8 Polymer linkage issues (i)

There are no chain breaks in this entry.

For Manuscript Review

6 Map visualisation (i)

This section contains visualisations of the EMDB entry EMD-38077. These allow visual inspection of the internal detail of the map and identification of artifacts.

Images derived from a raw map, generated by summing the deposited half-maps, are presented below the corresponding image components of the primary map to allow further visual inspection and comparison with those of the primary map.

6.1 Orthogonal projections (i)

6.1.1 Primary map

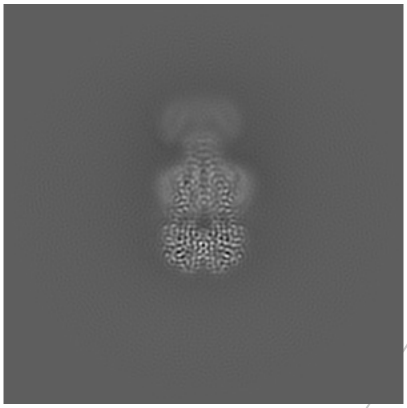

X

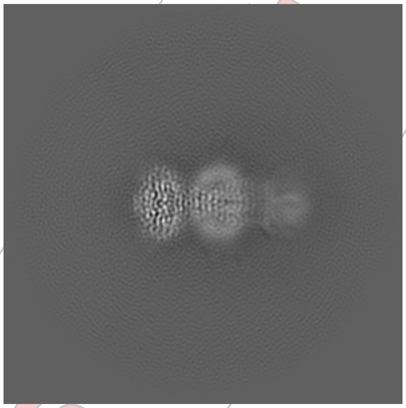

Y

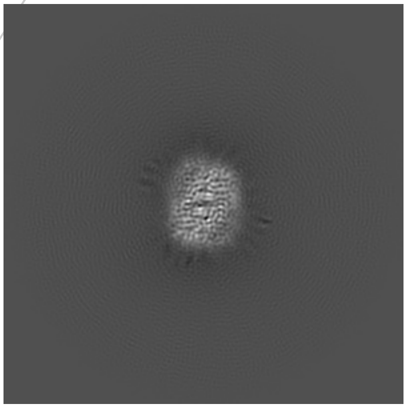

Z

6.1.2 Raw map

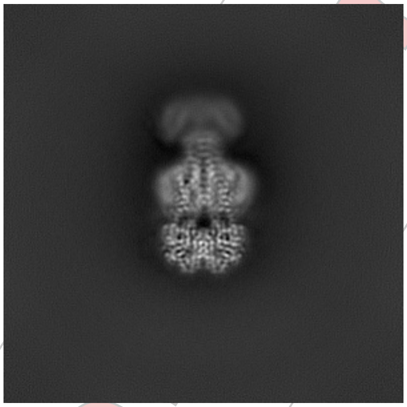

X

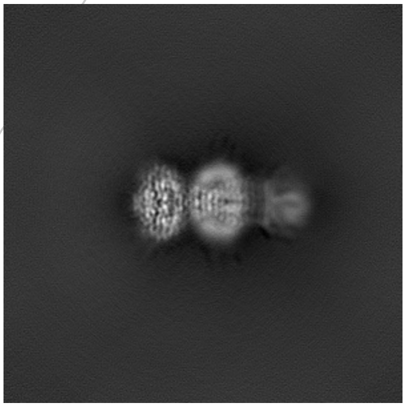

Y

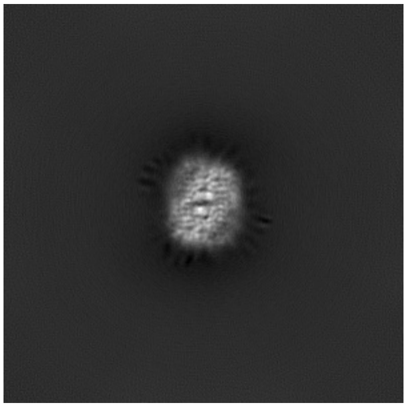

Z

The images above show the map projected in three orthogonal directions.

For Manuscript Review

6.2 Central slices (i)

6.2.1 Primary map

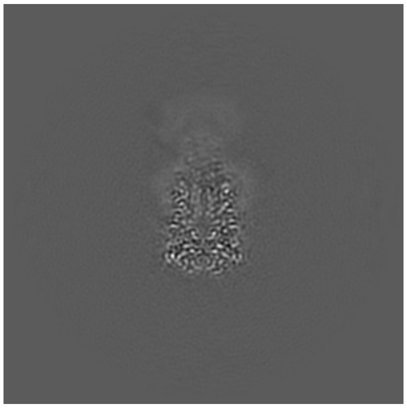
X Index: 180

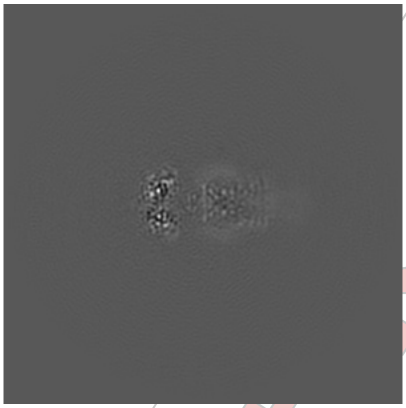
Y Index: 180

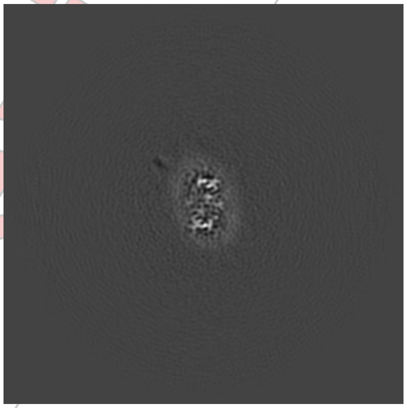
Z Index: 180

6.2.2 Raw map

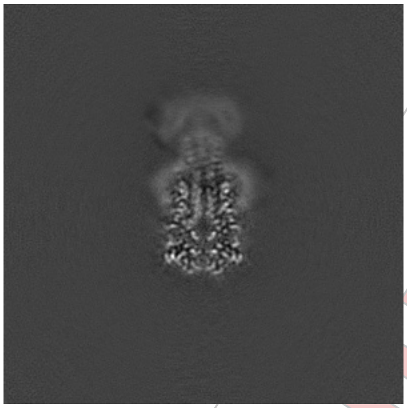
X Index: 180

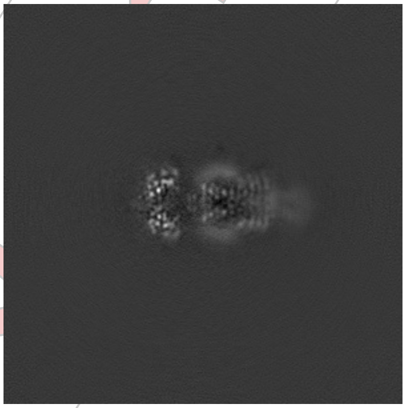
Y Index: 180

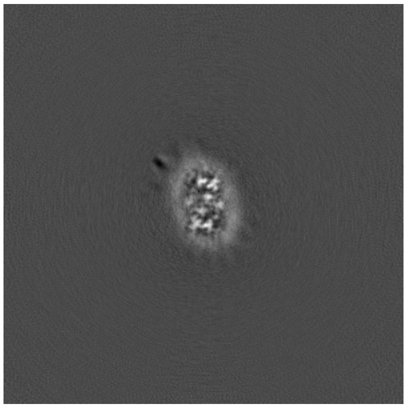
Z Index: 180

The images above show central slices of the map in three orthogonal directions.

For Manuscript Review

6.3 Largest variance slices (i)

6.3.1 Primary map

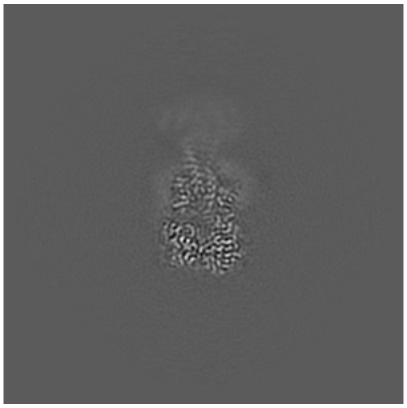
X Index: 185

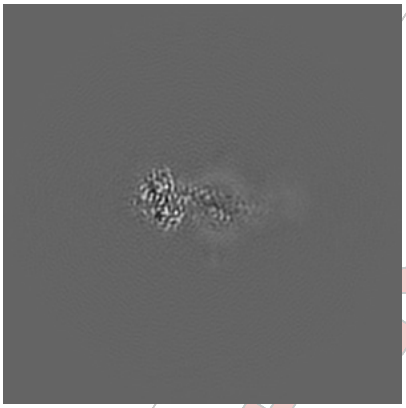
Y Index: 193

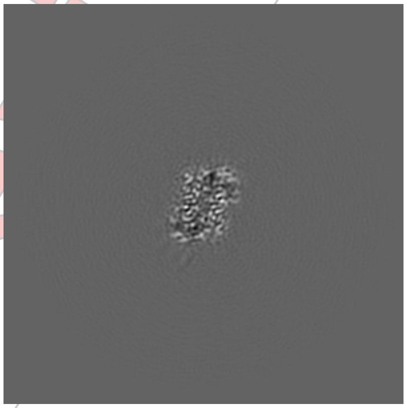
Z Index: 143

6.3.2 Raw map

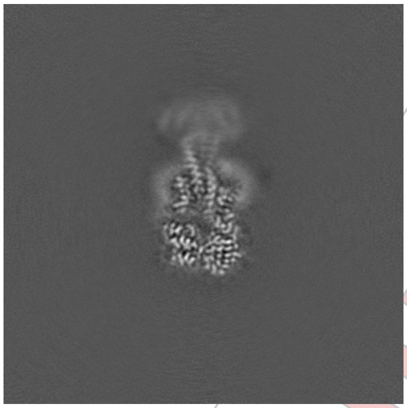
X Index: 185

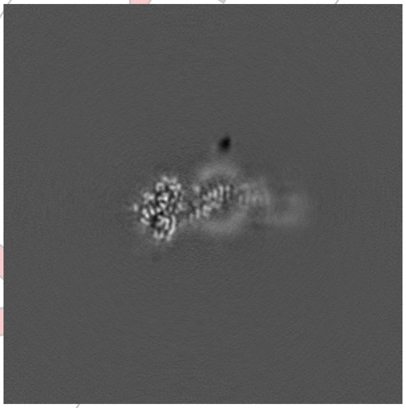
Y Index: 165

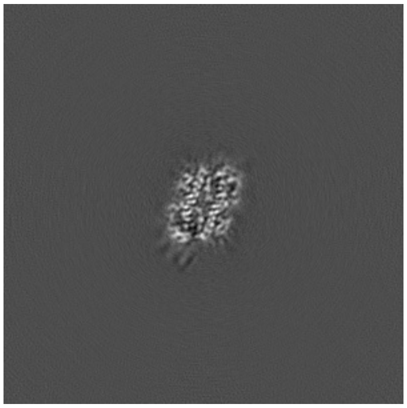
Z Index: 144

The images above show the largest variance slices of the map in three orthogonal directions.

For Manuscript Review

6.4 Orthogonal standard-deviation projections (False-color) (i)

6.4.1 Primary map

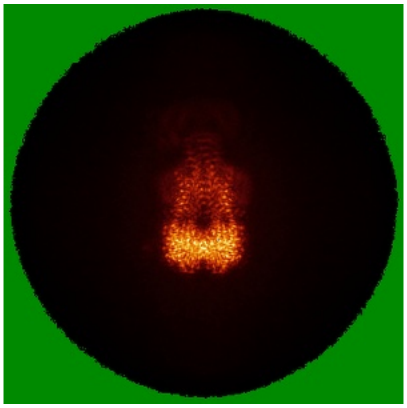

X

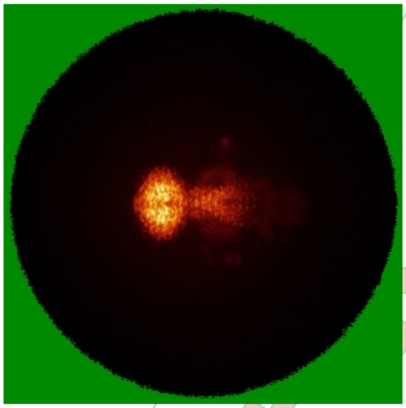

Y

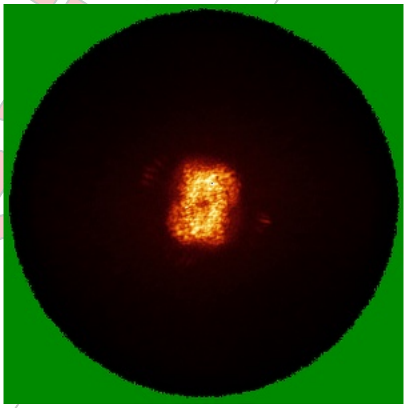

Z

6.4.2 Raw map

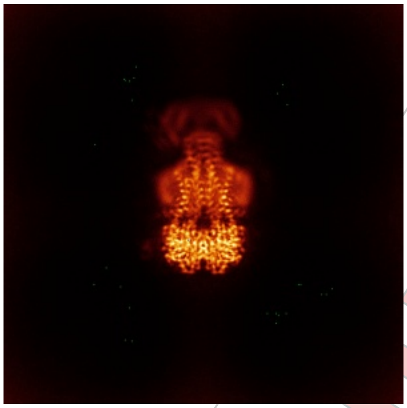

X

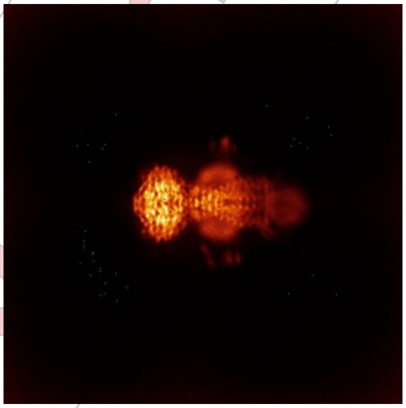

Y

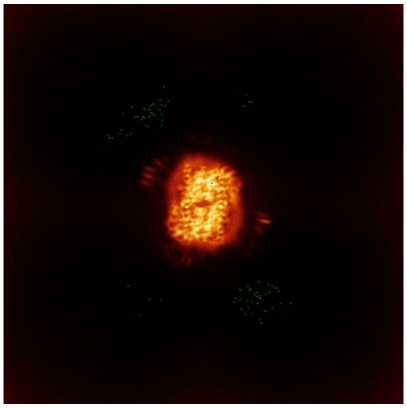

Z

The images above show the map standard deviation projections with false color in three orthogonal directions. Minimum values are shown in green, max in blue, and dark to light orange shades represent small to large values respectively.

For Manuscript Review

6.5 Orthogonal surface views (i)

6.5.1 Primary map

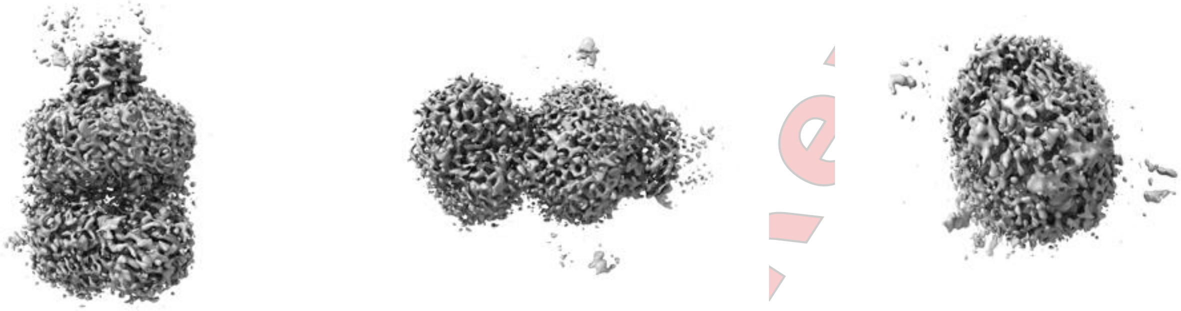

X Y Z

The images above show the 3D surface view of the map at the recommended contour level 0.1. These images, in conjunction with the slice images, may facilitate assessment of whether an appropriate contour level has been provided.

6.5.2 Raw map

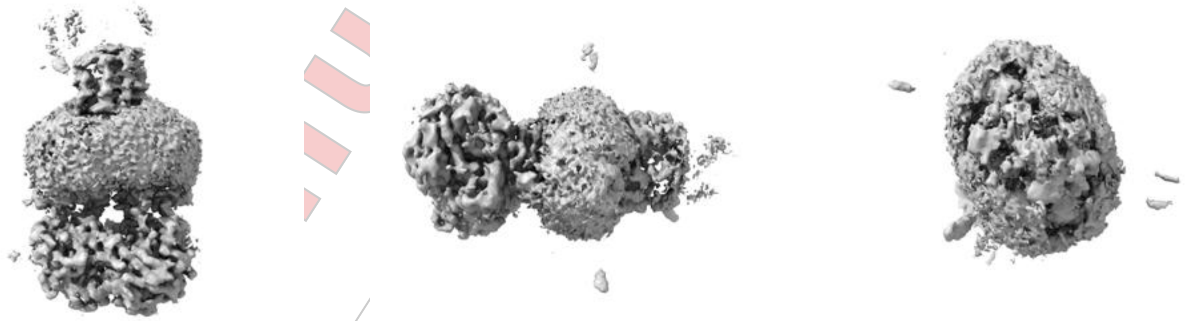

X Y Z

These images show the 3D surface of the raw map. The raw map's contour level was selected so that its surface encloses the same volume as the primary map does at its recommended contour level.

For Manuscript Review

6.6 Mask visualisation (i)

This section shows the 3D surface view of the primary map at 50% transparency overlaid with the specified mask at 0% transparency

A mask typically either:

- Encompasses the whole structure
- Separates out a domain, a functional unit, a monomer or an area of interest from a larger structure

6.6.1 D_1300042447_em-mask-volume_P1.map.V6 (i)

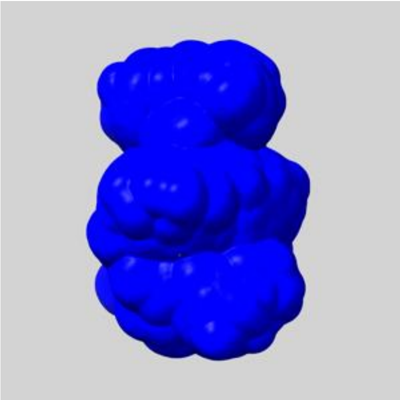

X

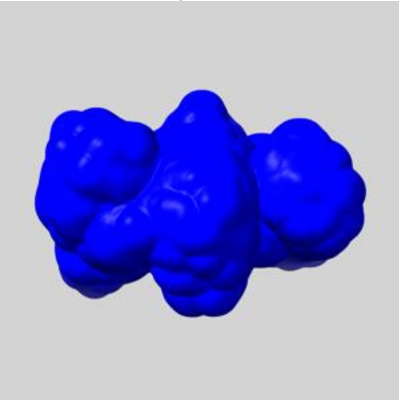

Y

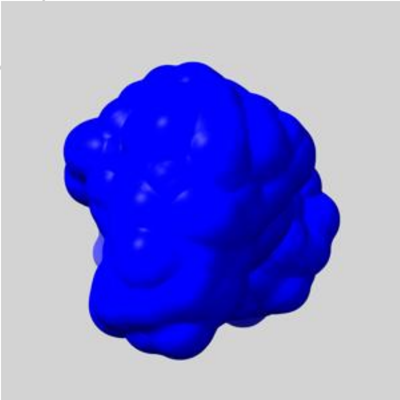

Z

For Manuscript Review

7 Map analysis (i)

This section contains the results of statistical analysis of the map.

7.1 Map-value distribution (i)

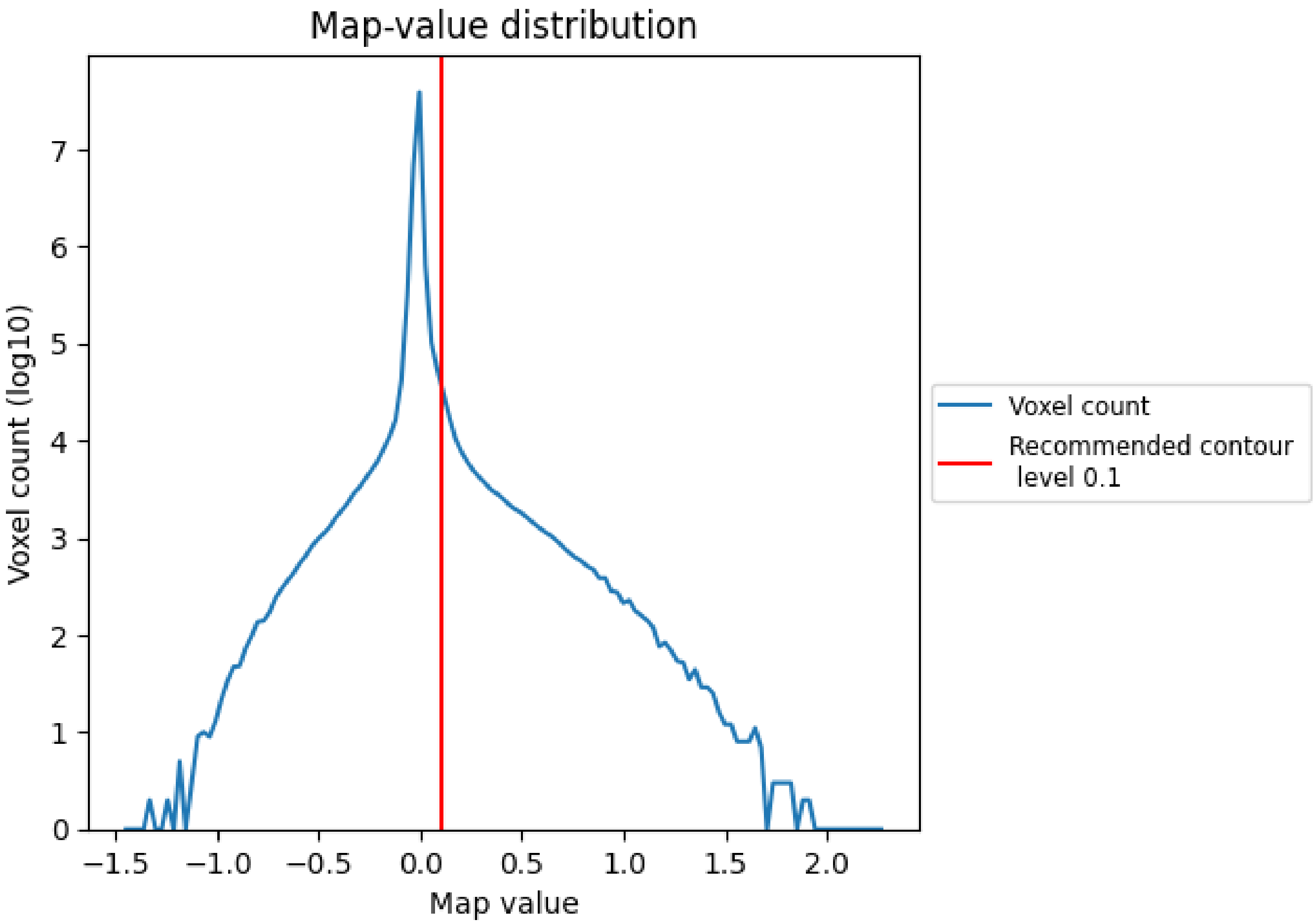

The map-value distribution is plotted in 128 intervals along the x-axis. The y-axis is logarithmic. A spike in this graph at zero usually indicates that the volume has been masked.

For Manuscript Review

7.2 Volume estimate (i)

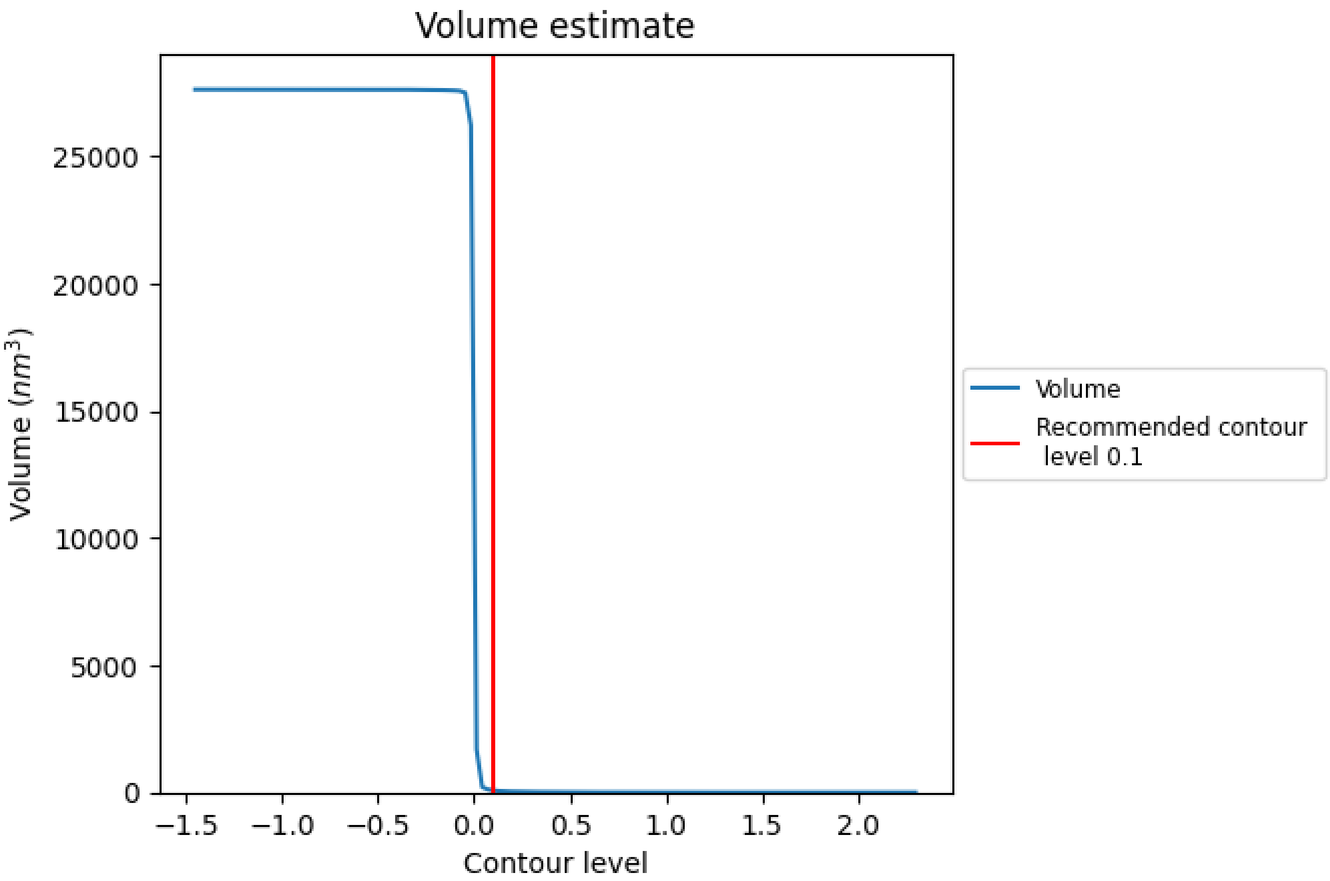

The volume at the recommended contour level is 79 nm^3; this corresponds to an approximate mass of 71 kDa.

The volume estimate graph shows how the enclosed volume varies with the contour level. The recommended contour level is shown as a vertical line and the intersection between the line and the curve gives the volume of the enclosed surface at the given level.

For Manuscript Review

7.3 Rotationally averaged power spectrum (i)

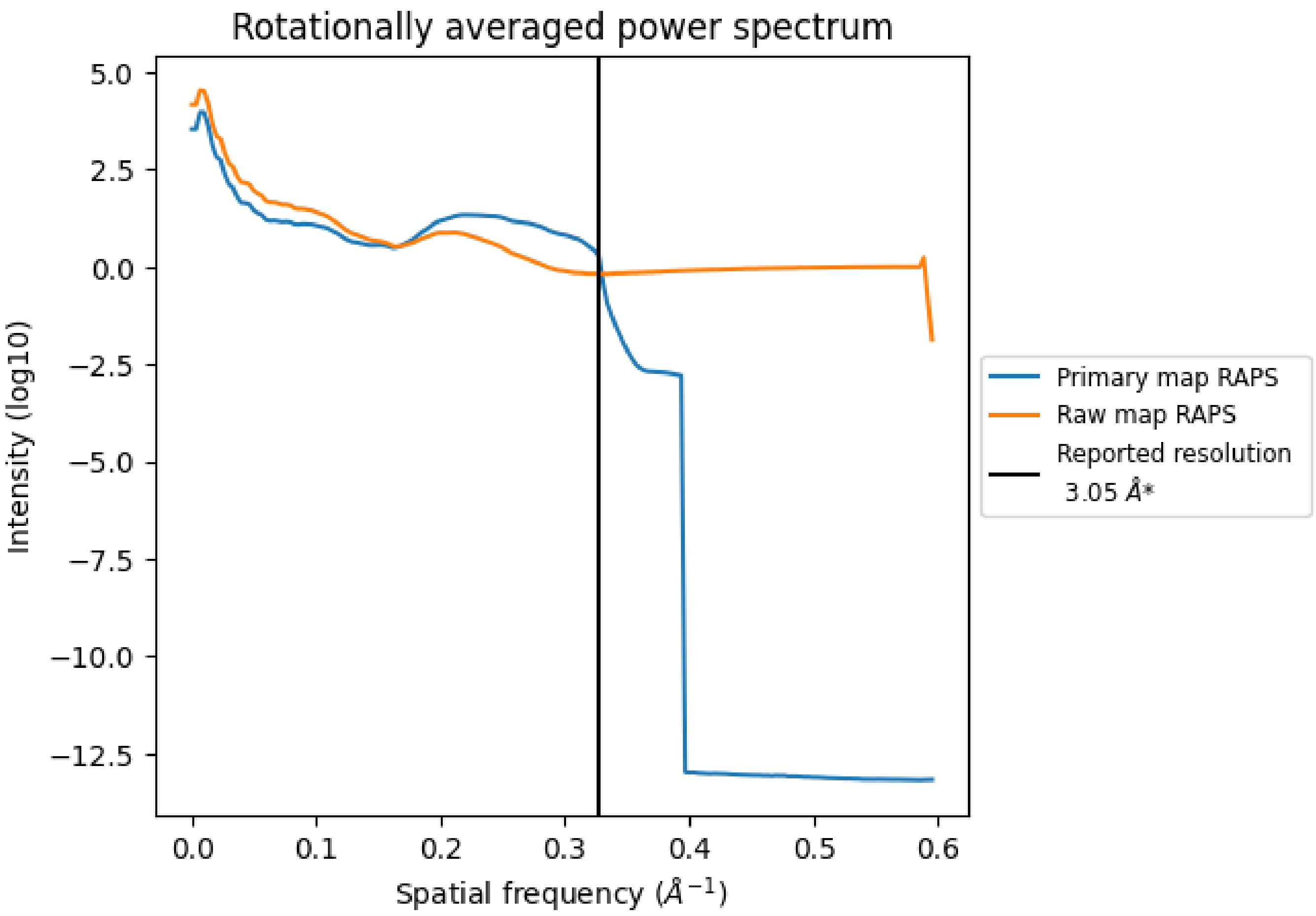

*Reported resolution corresponds to spatial frequency of 0.328 Å^{-1}

For Manuscript Review

8 Fourier-Shell correlation (i)

Fourier-Shell Correlation (FSC) is the most commonly used method to estimate the resolution of single-particle and subtomogram-averaged maps. The shape of the curve depends on the imposed symmetry, mask and whether or not the two 3D reconstructions used were processed from a common reference. The reported resolution is shown as a black line. A curve is displayed for the half-bit criterion in addition to lines showing the 0.143 gold standard cut-off and 0.5 cut-off.

8.1 FSC (i)

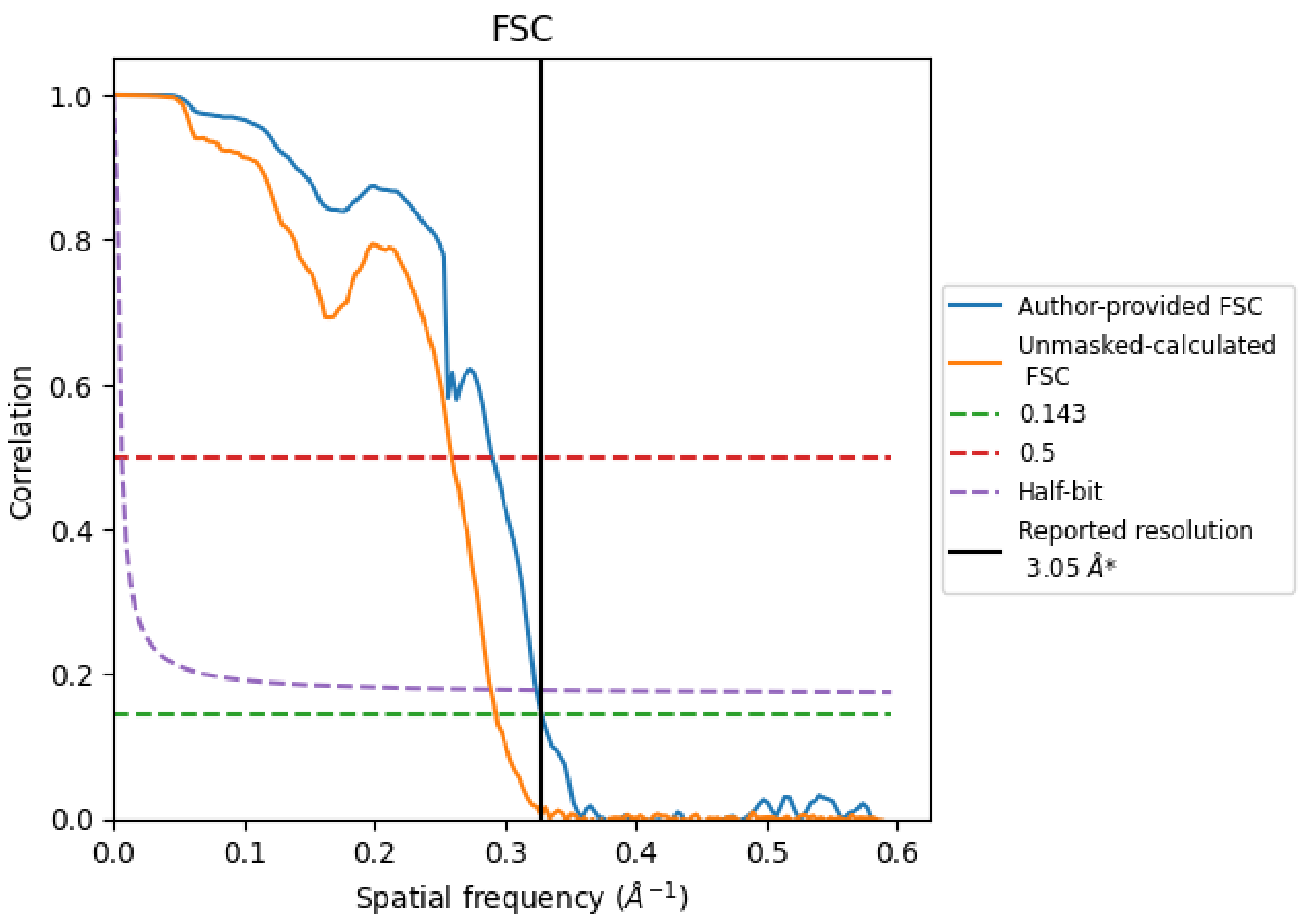

*Reported resolution corresponds to spatial frequency of 0.328 Å^{-1}

For Manuscript Review

8.2 Resolution estimates (i)

Resolution estimate (Å)	Estimation criterion (FSC cut-off)		
	0.143	0.5	Half-bit
Reported by author	3.05	-	-
Author-provided FSC curve	3.05	3.45	3.09
Unmasked-calculated*	3.41	3.86	3.46

*Resolution estimate based on FSC curve calculated by comparison of deposited half-maps. The value from deposited half-maps intersecting FSC 0.143 CUT-OFF 3.41 differs from the reported value 3.05 by more than 10 %

For Manuscript Review

9 Map-model fit (i)

This section contains information regarding the fit between EMDB map EMD-38077 and PDB model 8X61. Per-residue inclusion information can be found in section 3 on page 5.

9.1 Map-model overlay (i)

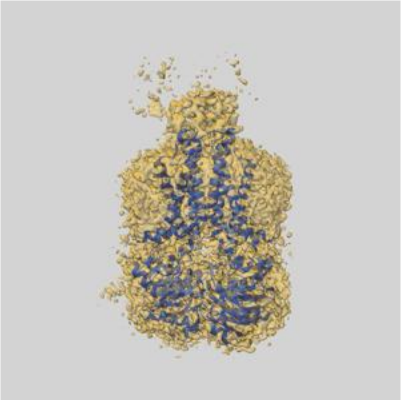

X

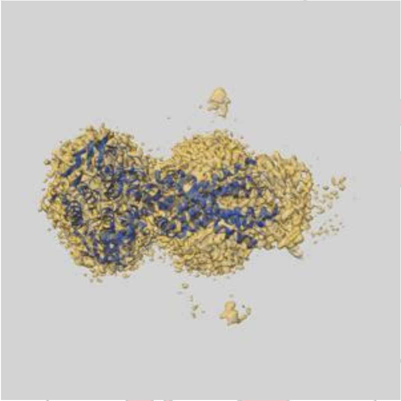

Y

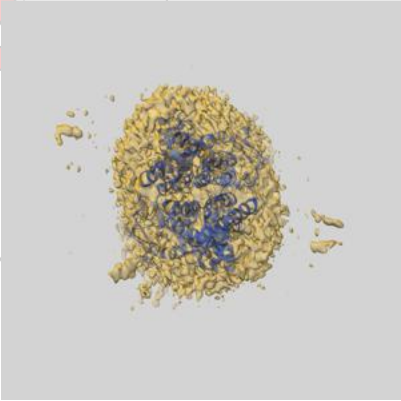

Z

The images above show the 3D surface view of the map at the recommended contour level 0.1 at 50% transparency in yellow overlaid with a ribbon representation of the model coloured in blue. These images allow for the visual assessment of the quality of fit between the atomic model and the map.

For Manuscript Review

9.2 Q-score mapped to coordinate model (i)

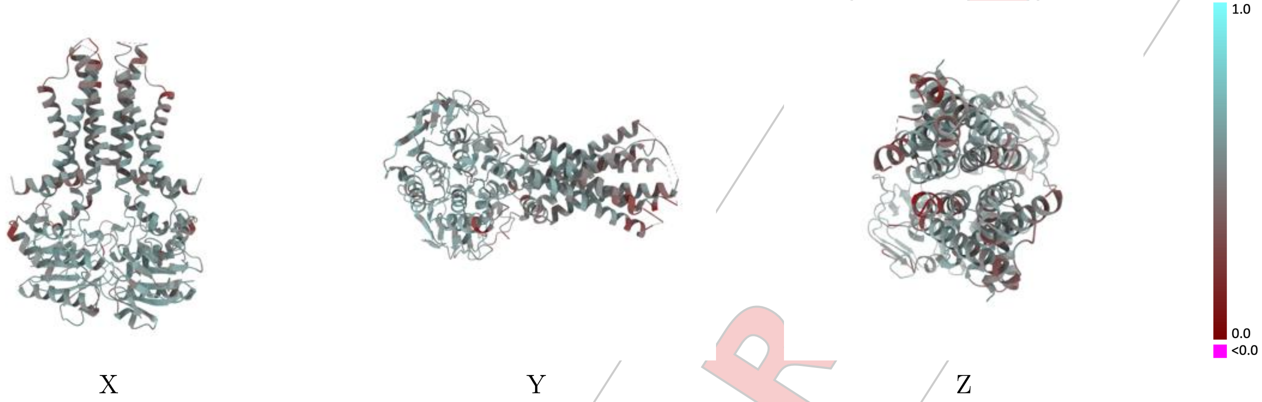

The images above show the model with each residue coloured according its Q-score. This shows their resolvability in the map with higher Q-score values reflecting better resolvability. Please note: Q-score is calculating the resolvability of atoms, and thus high values are only expected at resolutions at which atoms can be resolved. Low Q-score values may therefore be expected for many entries.

9.3 Atom inclusion mapped to coordinate model (i)

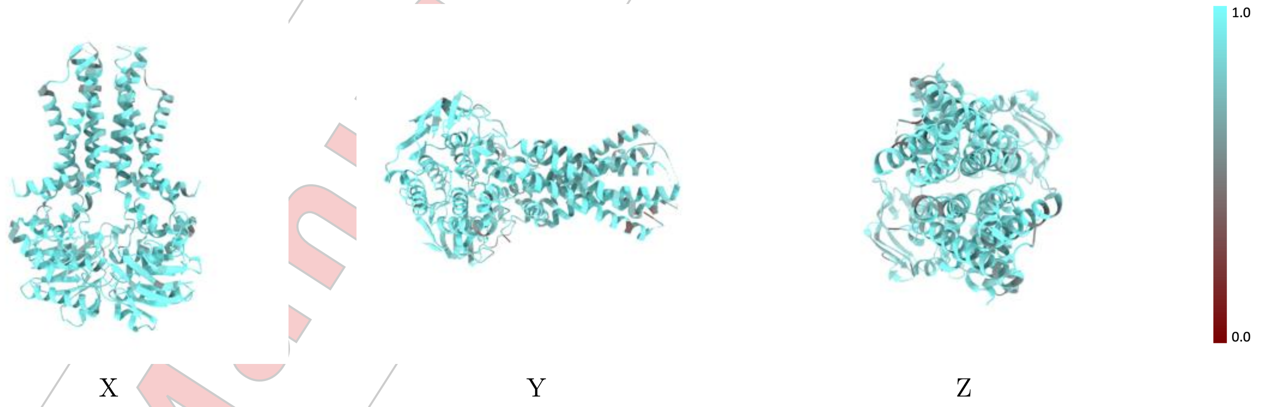

The images above show the model with each residue coloured according to its atom inclusion. This shows to what extent they are inside the map at the recommended contour level (0.1).

For Manuscript Review

9.4 Atom inclusion (i)

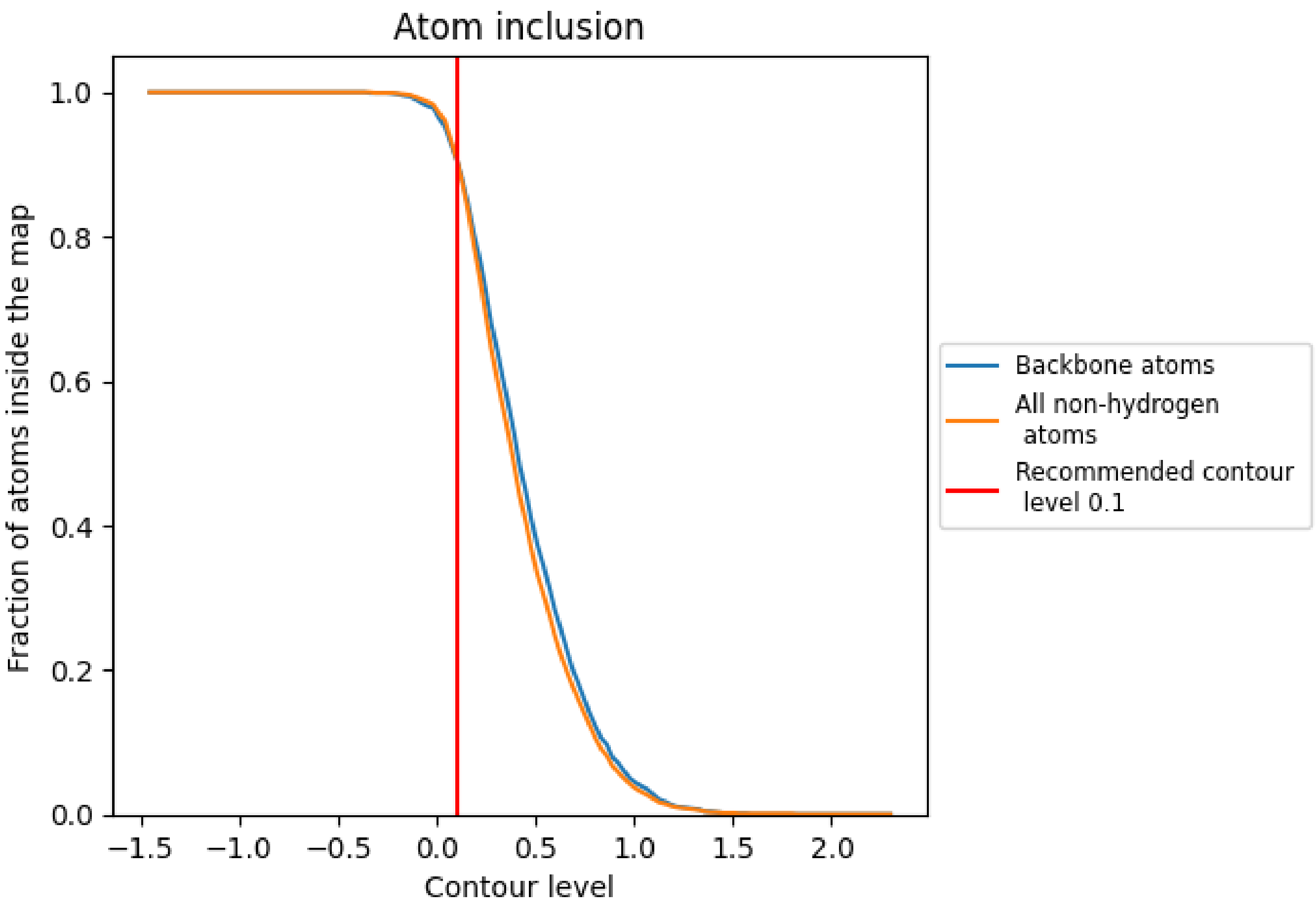

At the recommended contour level, 91% of all backbone atoms, 91% of all non-hydrogen atoms, are inside the map.

For Manuscript Review

9.5 Map-model fit summary (i)

The table lists the average atom inclusion at the recommended contour level (0.1) and Q-score for the entire model and for each chain.

Chain	Atom inclusion	Q-score
All	0.9090	0.5250
A	0.9320	0.5480
B	0.9290	0.5510
C	0.8840	0.4960
D	0.8700	0.4840

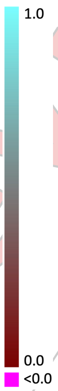

For Manuscript Review

Full wwPDB EM Validation Report (i)

Jan 30, 2024 – 01:00 PM JST

PDB ID	:	8Y3X
EMDB ID	:	EMD-38906
Title	:	Cell divisome sPG hydrolysis machinery FtsEX-EnvC
Deposited on	:	2024-01-29
Resolution	:	3.11 Å(reported)

This wwPDB validation report is for manuscript review

This is a Full wwPDB EM Validation Report.

This report is produced by the wwPDB biocuration pipeline after annotation of the structure.

We welcome your comments at *validation@mail.wwpdb.org*
A user guide is available at
https://www.wwpdb.org/validation/2017/EMValidationReportHelp
with specific help available everywhere you see the (i) symbol.

The types of validation reports are described at
http://www.wwpdb.org/validation/2017/FAQs#types.

The following versions of software and data (see references (i)) were used in the production of this report:

EMDB validation analysis	:	0.0.1.dev70
Mogul	:	1.8.5 (274361), CSD as541be (2020)
MolProbity	:	4.02b-467
buster-report	:	1.1.7 (2018)
Percentile statistics	:	20191225.v01 (using entries in the PDB archive December 25th 2019)
MapQ	:	1.9.9
Ideal geometry (proteins)	:	Engh & Huber (2001)
Ideal geometry (DNA, RNA)	:	Parkinson et al. (1996)
Validation Pipeline (wwPDB-VP)	:	2.36

For Manuscript Review

1 Overall quality at a glance (i)

The following experimental techniques were used to determine the structure:
ELECTRON MICROSCOPY

The reported resolution of this entry is 3.11 Å.

Percentile scores (ranging between 0-100) for global validation metrics of the entry are shown in the following graphic. The table shows the number of entries on which the scores are based.

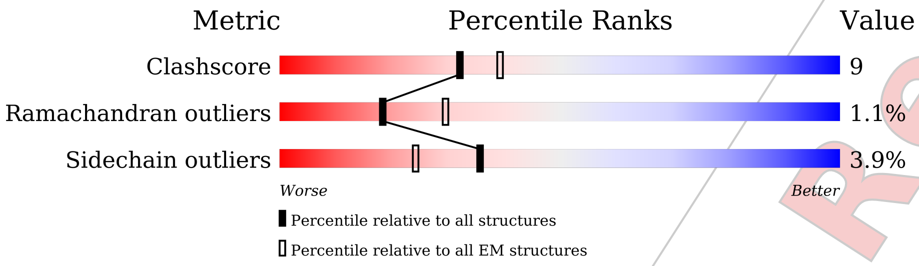

Metric	Whole archive (#Entries)	EM structures (#Entries)
Clashscore	158937	4297
Ramachandran outliers	154571	4023
Sidechain outliers	154315	3826

The table below summarises the geometric issues observed across the polymeric chains and their fit to the map. The red, orange, yellow and green segments of the bar indicate the fraction of residues that contain outliers for >=3, 2, 1 and 0 types of geometric quality criteria respectively. A grey segment represents the fraction of residues that are not modelled. The numeric value for each fraction is indicated below the corresponding segment, with a dot representing fractions <=5% The upper red bar (where present) indicates the fraction of residues that have poor fit to the EM map (all-atom inclusion < 40%). The numeric value is given above the bar.

Mol	Chain	Length	Quality of chain
1	A	222	82% 16% • •
1	B	222	78% 19% • •
2	C	352	8% / 63% 18% • 15%
2	D	352	8% / 73% 11% 15%
3	E	419	8% / 24% • 74%

For Manuscript Review

2 Entry composition (i)

There are 4 unique types of molecules in this entry. The entry contains 7757 atoms, of which 0 are hydrogens and 0 are deuteriums.

In the tables below, the AltConf column contains the number of residues with at least one atom in alternate conformation and the Trace column contains the number of residues modelled with at most 2 atoms.

- Molecule 1 is a protein called Cell division ATP-binding protein FtsE.

Mol	Chain	Residues	Atoms					AltConf	Trace
1	A	218	Total 1669	C 1054	N 309	O 298	S 8	0	0
1	B	218	Total 1670	C 1053	N 312	O 297	S 8	0	0

There are 2 discrepancies between the modelled and reference sequences:

Chain	Residue	Modelled	Actual	Comment	Reference
A	163	GLN	GLU	conflict	UNP P0A9R7
B	163	GLN	GLU	conflict	UNP P0A9R7

- Molecule 2 is a protein called Cell division protein FtsX.

Mol	Chain	Residues	Atoms					AltConf	Trace
2	C	299	Total 1918	C 1239	N 336	O 336	S 7	0	0
2	D	299	Total 1888	C 1213	N 331	O 337	S 7	0	0

- Molecule 3 is a protein called Murein hydrolase activator EnvC.

Mol	Chain	Residues	Atoms				AltConf	Trace
3	E	111	Total 550	C 328	N 111	O 111	0	0

- Molecule 4 is ADENOSINE-5'-TRIPHOSPHATE (three-letter code: ATP) (formula: $C_{10}H_{16}N_5O_{13}P_3$) (labeled as "Ligand of Interest" by depositor).

For Manuscript Review

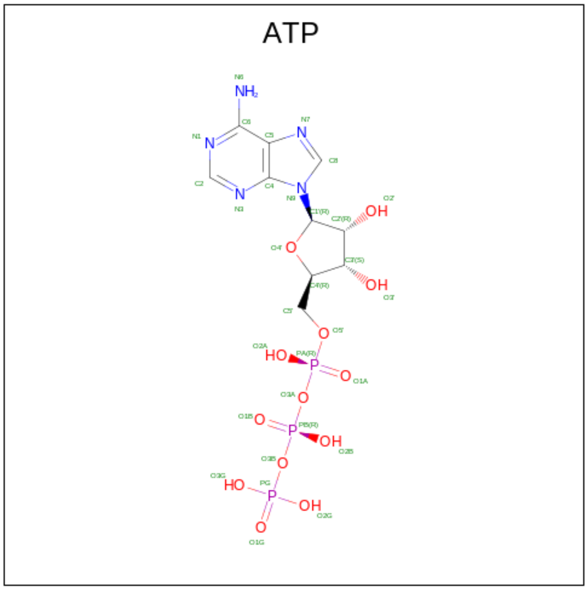

Mol	Chain	Residues	Atoms					AltConf
4	A	1	Total 31	C 10	N 5	O 13	P 3	0
4	B	1	Total 31	C 10	N 5	O 13	P 3	0

For Manuscript Review

3 Residue-property plots (i)

These plots are drawn for all protein, RNA, DNA and oligosaccharide chains in the entry. The first graphic for a chain summarises the proportions of the various outlier classes displayed in the second graphic. The second graphic shows the sequence view annotated by issues in geometry and atom inclusion in map density. Residues are color-coded according to the number of geometric quality criteria for which they contain at least one outlier: green = 0, yellow = 1, orange = 2 and red = 3 or more. A red diamond above a residue indicates a poor fit to the EM map for this residue (all-atom inclusion $< 40\%$). Stretches of 2 or more consecutive residues without any outlier are shown as a green connector. Residues present in the sample, but not in the model, are shown in grey.

- Molecule 1: Cell division ATP-binding protein FtsE

Chain A:

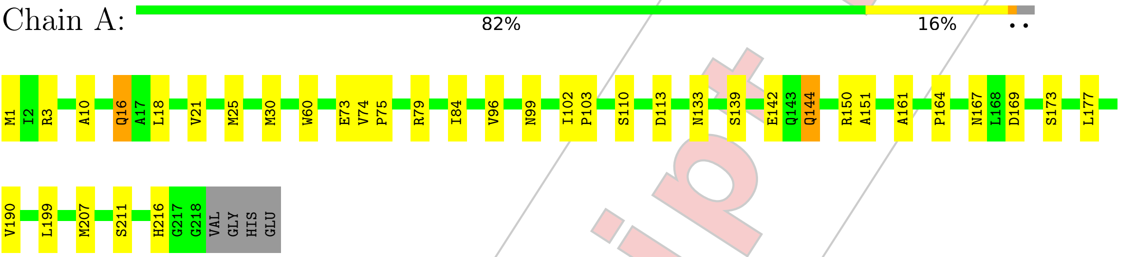

- Molecule 1: Cell division ATP-binding protein FtsE

Chain B:

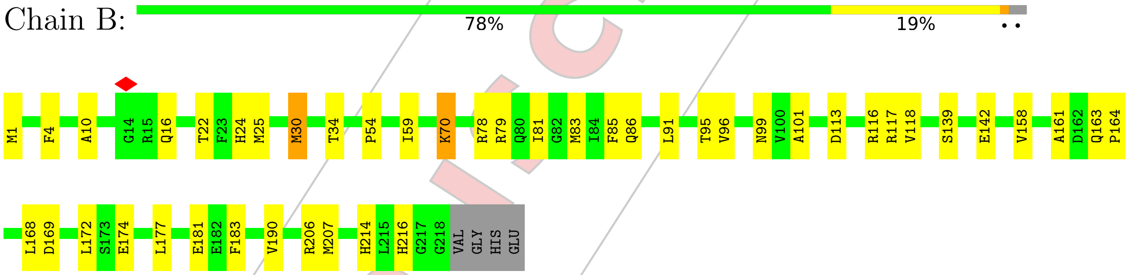

- Molecule 2: Cell division protein FtsX

Chain C:

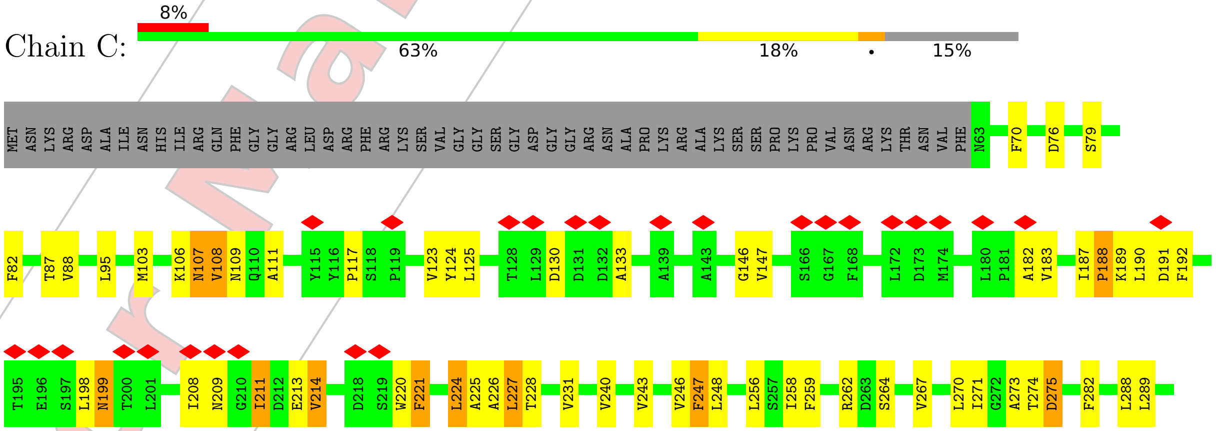

For Manuscript Review

S292 G293 L300 S301 E302 V305 S309 A316 K322 I325 F330 D331 E332 C340 T360 P361 GLU

- Molecule 2: Cell division protein FtsX

Chain D: 8% 73% 11% 15%

MET ASN LYS ARG ASP ALA ILE ASN HIS ILE ARG GLN PHE GLY GLY ARG LEU ASP ARG PHE ARG LYS SER VAL GLY GLY SER GLY ASP GLY GLY ARG ASN ALA PRO LYS ARG ALA LYS SER SER PRO LYS PRO VAL ASN ARG LYS THR ASN VAL PHE M63 H71 L74 Q75 D76 L77

K78 S79 T87 V90 L95 L125 T128 L129 D130 D131 D132 A133 A134 A135 A139 A143 E144 E148 K149 V150 D157 G160 N164 W165 S166 L172 D173 I187 D191 E196 S197 L198 N199 T200 L201 R202 I211 D218 A222 G239

V243 V246 F247 I250 L256 S257 I258 D263 L270 I271 G272 F282 L289 I299 E302 I303 L304 A311 V312 A313 Q317 K322 F323 D324 F330 C340 I343 H355 H358 P361 GLU

- Molecule 3: Murein hydrolase activator EnvC

Chain E: 8% 24% • 74%

MET THR ARG ALA VAL LYS PRO ARG ARG PHE ALA ILE ARG PRO ILE ILE TYR ALA SER VAL LEU SER ALA GLY VAL LEU LEU CYS ALA PHE SER ALA HIS ALA ASP GLU ARG ASP GLN LEU LYS SER ILE GLN ALA ASP ILE ALA ALA LYS GLU ARG ALA VAL ARG GLN LYS GLN GLN GLN

ARG ALA SER LEU LEU ALA GLN LEU LYS LYS GLN GLU GLU ALA ILE SER GLU ALA THR R80 K81 L82 R83 E84 T85 Q86 N87 T88 L89 N90 Q91 L92 N93 K94 K110 A111 E114 Q120 L121 D122 A123 R126 Q127 G128 E129 I133 Q134 Q143 Q146 Q149 A150

G153 Y154 A164 R177 E181 E182 K183 Q184 S185 E186 Q187 Q188 T189 L190 LEU TYR GLU GLN ARG ALA ALA GLN ALA LYS LEU THR GLN ALA LEU ASN GLU ARG LYS LYS THR LEU ALA GLY LEU GLU SER SER ILE GLN GLN GLY GLN GLN GLN LEU SER GLU LEU ARG ALA ASN GLU

SER ARG LEU ARG ASN SER ILE ALA ARG ALA GLU ALA ALA LYS ALA ARG ALA GLU ARG GLU ALA ARG GLU ALA GLN ALA VAL ARG ASP ARG GLN LYS GLU ALA THR ARG LYS GLY THR THR TYR LYS PRO THR GLU SER GLU LYS SER LEU MET SER ARG THR GLY GLY LEU GLY ALA

PRO ARG GLY GLN ALA PHE TRP PRO VAL ARG GLY PRO THR LEU HIS ARG TYR GLY GLU GLN LEU GLN GLY GLU LEU ARG TRP LYS GLY MET VAL ILE GLY ALA SER GLU GLY THR GLU VAL LYS ALA ILE ALA ASP GLY ARG VAL ILE LEU ALA ASP TRP LEU GLN GLY TYR GLY LEU VAL

VAL VAL VAL GLU HIS GLY LYS GLY ASP MET SER LEU TYR GLY TYR ASN GLN SER ALA LEU VAL SER VAL GLY SER GLN VAL ARG ALA GLY GLN PRO ILE ALA LEU VAL GLY SER SER GLY GLY GLN GLY ARG PRO SER LEU TYR PHE GLU ILE ARG ARG GLN GLY GLN ALA VAL ASN PRO

GLN PRO TRP LEU GLY ARG

For Manuscript Review

4 Experimental information (i)

Property	Value	Source
EM reconstruction method	SINGLE PARTICLE	Depositor
Imposed symmetry	POINT, Not provided	
Number of particles used	4039317	Depositor
Resolution determination method	FSC 0.143 CUT-OFF	Depositor
CTF correction method	NONE	Depositor
Microscope	FEI TITAN KRIOS	Depositor
Voltage (kV)	300	Depositor
Electron dose ($e^-/\text{Å}^2$)	60	Depositor
Minimum defocus (nm)	1000	Depositor
Maximum defocus (nm)	3000	Depositor
Magnification	Not provided	
Image detector	GATAN K3 BIOQUANTUM (6k x 4k)	Depositor
Maximum map value	1.868	Depositor
Minimum map value	-1.275	Depositor
Average map value	-0.000	Depositor
Map value standard deviation	0.012	Depositor
Recommended contour level	0.1	Depositor
Map size (Å)	483.84, 483.84, 483.84	wwPDB
Map dimensions	576, 576, 576	wwPDB
Map angles (°)	90.0, 90.0, 90.0	wwPDB
Pixel spacing (Å)	0.84, 0.84, 0.84	Depositor

For Manuscript Review

5 Model quality (i)

5.1 Standard geometry (i)

Bond lengths and bond angles in the following residue types are not validated in this section: ATP

The Z score for a bond length (or angle) is the number of standard deviations the observed value is removed from the expected value. A bond length (or angle) with $|Z| > 5$ is considered an outlier worth inspection. RMSZ is the root-mean-square of all Z scores of the bond lengths (or angles).

Mol	Chain	Bond lengths		Bond angles	
		RMSZ	#$\|Z\|$ >5	RMSZ	#$\|Z\|$ >5
1	A	0.38	0/1698	0.58	0/2290
1	B	0.38	0/1699	0.57	0/2289
2	C	0.41	0/1950	0.54	0/2681
2	D	0.27	0/1918	0.48	1/2638 (0.0%)
3	E	0.22	0/549	0.42	0/764
All	All	0.36	0/7814	0.54	1/10662 (0.0%)

There are no bond length outliers.

All (1) bond angle outliers are listed below:

Mol	Chain	Res	Type	Atoms	Z	Observed(^{o})	Ideal(^{o})
2	D	270	LEU	CA-CB-CG	5.82	128.68	115.30

There are no chirality outliers.

There are no planarity outliers.

5.2 Too-close contacts (i)

In the following table, the Non-H and H(model) columns list the number of non-hydrogen atoms and hydrogen atoms in the chain respectively. The H(added) column lists the number of hydrogen atoms added and optimized by MolProbity. The Clashes column lists the number of clashes within the asymmetric unit, whereas Symm-Clashes lists symmetry-related clashes.

Mol	Chain	Non-H	H(model)	H(added)	Clashes	Symm-Clashes
1	A	1669	0	1695	25	0
1	B	1670	0	1695	27	0
2	C	1918	0	1667	61	0
2	D	1888	0	1594	26	0
3	E	550	0	265	9	0

Continued on next page...

For Manuscript Review

Continued from previous page...

Mol	Chain	Non-H	H(model)	H(added)	Clashes	Symm-Clashes
4	A	31	0	12	0	0
4	B	31	0	12	0	0
All	All	7757	0	6940	138	0

The all-atom clashscore is defined as the number of clashes found per 1000 atoms (including hydrogen atoms). The all-atom clashscore for this structure is 9.

All (138) close contacts within the same asymmetric unit are listed below, sorted by their clash magnitude.

Atom-1	Atom-2	Interatomic distance (Å)	Clash overlap (Å)
2:C:123:VAL:CA	2:C:214:VAL:HA	1.86	1.06
2:C:123:VAL:HA	2:C:214:VAL:CA	1.86	1.05
2:C:125:LEU:CA	2:C:211:ILE:HA	1.91	0.99
2:C:125:LEU:HA	2:C:211:ILE:HA	0.99	0.98
2:C:125:LEU:HA	2:C:211:ILE:CA	1.93	0.97
3:E:146:GLN:HA	3:E:150:ALA:HB3	1.47	0.94
2:C:123:VAL:HA	2:C:214:VAL:HA	0.94	0.85
2:C:117:PRO:HB2	2:C:220:TRP:N	1.95	0.82
2:C:117:PRO:HG2	2:C:220:TRP:CB	2.11	0.80
1:A:79:ARG:HG3	2:D:272:GLY:H	1.46	0.79
2:C:225:ALA:HA	2:C:228:THR:HG22	1.66	0.77
2:C:117:PRO:HG3	2:C:220:TRP:HA	1.66	0.76
1:A:30:MET:HG3	1:A:190:VAL:HB	1.68	0.74
1:A:139:SER:OG	1:A:142:GLU:OE1	2.08	0.72
1:A:79:ARG:NH2	2:D:272:GLY:O	2.21	0.71
2:C:248:LEU:HB3	2:D:90:VAL:HG22	1.73	0.71
2:D:130:ASP:H	2:D:133:ALA:HB3	1.57	0.69
1:B:1:MET:N	1:B:25:MET:O	2.26	0.69
1:A:169:ASP:O	1:A:173:SER:OG	2.12	0.67
1:A:167:ASN:OD1	1:B:86:GLN:NE2	2.28	0.66
2:D:270:LEU:HD23	2:D:271:ILE:HG12	1.78	0.65
1:B:113:ASP:OD2	1:B:117:ARG:NH1	2.30	0.64
2:C:259:PHE:O	2:C:262:ARG:NH1	2.30	0.64
2:C:117:PRO:CG	2:C:220:TRP:HA	2.27	0.63
2:C:117:PRO:CG	2:C:220:TRP:CB	2.77	0.62
1:A:10:ALA:HB2	1:A:16:GLN:HE21	1.66	0.60
1:B:78:ARG:HH11	2:C:270:LEU:HA	1.65	0.60
2:C:130:ASP:H	2:C:133:ALA:HB3	1.67	0.60
2:C:117:PRO:CG	2:C:220:TRP:CA	2.81	0.58
1:B:30:MET:HG3	1:B:190:VAL:HB	1.85	0.58
2:C:246:VAL:HG13	2:C:247:PHE:HD1	1.69	0.58

Continued on next page...

For Manuscript Review

Continued from previous page...

Atom-1	Atom-2	Interatomic distance (Å)	Clash overlap (Å)
2:C:124:TYR:H	2:C:213:GLU:C	2.07	0.57
1:A:84:ILE:HG23	1:A:151:ALA:HB2	1.86	0.57
3:E:110:LYS:O	3:E:114:GLU:CB	2.52	0.57
1:A:1:MET:N	1:A:25:MET:O	2.34	0.57
2:C:76:ASP:O	2:C:79:SER:OG	2.23	0.57
1:B:34:THR:OG1	1:B:207:MET:SD	2.61	0.56
2:D:95:LEU:HG	2:D:246:VAL:HG21	1.88	0.56
3:E:122:ASP:HA	3:E:126:ARG:H	1.70	0.56
1:B:161:ALA:HB1	1:B:164:PRO:HG3	1.88	0.56
2:C:316:ALA:HB1	2:C:322:LYS:HA	1.87	0.56
1:B:79:ARG:NH2	2:C:273:ALA:O	2.38	0.56
2:D:87:THR:HG21	2:D:289:LEU:HD21	1.88	0.56
1:A:103:PRO:HA	2:D:271:ILE:HD11	1.88	0.56
1:B:113:ASP:OD1	1:B:116:ARG:NH2	2.38	0.56
3:E:150:ALA:H	3:E:154:TYR:H	1.54	0.55
1:A:150:ARG:HD2	2:D:270:LEU:HD11	1.89	0.55
2:D:239:GLY:O	2:D:243:VAL:HG13	2.07	0.55
1:A:169:ASP:OD1	1:A:169:ASP:N	2.33	0.54
2:C:227:LEU:O	2:C:231:VAL:HB	2.07	0.54
1:B:169:ASP:N	1:B:169:ASP:OD1	2.40	0.54
2:C:117:PRO:HB2	2:C:220:TRP:H	1.73	0.53
1:A:161:ALA:HB1	1:A:164:PRO:HG3	1.90	0.53
2:C:146:GLY:HA3	2:C:192:PHE:H	1.73	0.53
1:B:86:GLN:NE2	1:B:163:GLN:OE1	2.41	0.53
2:D:160:GLY:O	2:D:164:ASN:ND2	2.42	0.52
1:B:83:MET:HG2	1:B:85:PHE:CE1	2.44	0.52
1:A:211:SER:HB3	1:A:216:HIS:CE1	2.44	0.52
2:C:360:THR:HG23	2:C:360:THR:O	2.10	0.51
2:D:198:LEU:O	2:D:202:ARG:N	2.31	0.51
2:C:70:PHE:HE1	2:C:288:LEU:HD11	1.75	0.51
2:C:124:TYR:O	2:C:213:GLU:N	2.39	0.50
2:C:198:LEU:O	2:C:199:ASN:C	2.49	0.50
2:C:107:ASN:OD1	2:C:325:ILE:HA	2.12	0.50
1:B:4:PHE:O	1:B:22:THR:HA	2.12	0.50
2:D:299:ILE:O	2:D:303:ILE:HG12	2.12	0.50
1:B:169:ASP:OD1	1:B:172:LEU:HB2	2.13	0.49
2:C:87:THR:HG21	2:C:289:LEU:HD21	1.93	0.49
2:C:189:LYS:O	2:C:190:LEU:C	2.50	0.49
2:D:125:LEU:HA	2:D:211:ILE:HA	1.95	0.49
2:C:274:THR:HG22	2:C:275:ASP:H	1.78	0.49
2:C:146:GLY:HA3	2:C:192:PHE:CB	2.43	0.48

Continued on next page...

For Manuscript Review

Continued from previous page...

Atom-1	Atom-2	Interatomic distance (Å)	Clash overlap (Å)
1:B:24:HIS:O	1:B:206:ARG:NH1	2.45	0.48
2:C:146:GLY:O	2:C:188:PRO:HA	2.14	0.48
2:C:225:ALA:C	2:C:227:LEU:H	2.17	0.48
2:C:147:VAL:N	2:C:189:LYS:H	2.11	0.47
2:C:267:VAL:O	2:C:271:ILE:HG13	2.14	0.47
2:C:88:VAL:HG13	2:C:293:GLY:HA2	1.97	0.47
2:C:146:GLY:HA3	2:C:192:PHE:N	2.30	0.47
2:D:247:PHE:CZ	2:D:343:ILE:HD11	2.49	0.47
1:B:70:LYS:H	1:B:70:LYS:HD2	1.79	0.47
2:C:125:LEU:N	2:C:182:ALA:O	2.48	0.47
2:C:106:LYS:O	2:C:111:ALA:HB2	2.15	0.47
3:E:123:ALA:HA	3:E:128:GLY:HA3	1.97	0.47
2:C:309:SER:HB2	2:C:325:ILE:HG21	1.98	0.46
2:D:355:HIS:HA	2:D:358:HIS:HD2	1.81	0.46
2:C:117:PRO:CB	2:C:220:TRP:N	2.74	0.46
2:C:190:LEU:O	2:C:191:ASP:C	2.53	0.46
1:B:214:HIS:HB3	1:B:216:HIS:CD2	2.51	0.45
2:C:95:LEU:HD11	2:C:340:CYS:SG	2.56	0.45
2:C:240:VAL:HA	2:C:243:VAL:HG22	1.98	0.45
2:C:258:ILE:HG13	2:C:282:PHE:CD1	2.51	0.45
1:A:99:ASN:O	1:A:102:ILE:HG22	2.17	0.45
2:D:250:ILE:HG21	2:D:343:ILE:HG22	1.98	0.45
2:C:187:ILE:O	2:C:188:PRO:C	2.55	0.45
2:C:300:LEU:C	2:C:302:GLU:H	2.21	0.45
2:D:302:GLU:OE2	2:D:303:ILE:HD13	2.17	0.45
2:D:74:LEU:HD23	2:D:74:LEU:HA	1.85	0.44
2:D:148:GLU:N	2:D:187:ILE:O	2.51	0.44
2:D:270:LEU:HD23	2:D:271:ILE:CG1	2.46	0.44
1:B:10:ALA:HB2	1:B:16:GLN:OE1	2.18	0.44
1:A:102:ILE:HG23	1:A:103:PRO:HD3	2.00	0.44
2:C:107:ASN:HB3	2:C:108:VAL:HG23	2.00	0.44
2:C:224:LEU:HD13	2:C:227:LEU:HD21	2.00	0.43
3:E:149:GLN:HA	3:E:154:TYR:N	2.33	0.43
1:B:139:SER:OG	1:B:142:GLU:OE1	2.32	0.43
1:B:95:THR:O	1:B:99:ASN:ND2	2.51	0.43
1:A:110:SER:OG	1:A:113:ASP:OD2	2.35	0.43
1:A:3:ARG:HB3	1:A:60:TRP:HB2	2.00	0.43
1:A:96:VAL:HG13	1:A:133:ASN:O	2.18	0.43
2:C:103:MET:O	2:C:107:ASN:HB2	2.18	0.43
1:B:101:ALA:HB2	1:B:118:VAL:HG21	2.00	0.43
2:D:95:LEU:HD11	2:D:340:CYS:SG	2.58	0.43

Continued on next page...

For Manuscript Review

Continued from previous page...

Atom-1	Atom-2	Interatomic distance (Å)	Clash overlap (Å)
2:C:271:ILE:HG22	2:C:271:ILE:O	2.19	0.43
3:E:146:GLN:O	3:E:153:GLY:N	2.37	0.43
3:E:150:ALA:N	3:E:154:TYR:H	2.15	0.42
2:C:103:MET:HG3	2:C:305:VAL:HG21	2.01	0.42
1:B:91:LEU:HD22	2:C:264:SER:HB2	2.00	0.42
2:C:300:LEU:HD12	2:C:300:LEU:HA	1.83	0.42
1:B:177:LEU:O	1:B:181:GLU:HG3	2.20	0.42
1:A:18:LEU:HD22	1:A:21:VAL:HG21	2.01	0.42
1:A:102:ILE:CG2	1:A:103:PRO:HD3	2.50	0.42
2:C:246:VAL:HG13	2:C:247:PHE:CD1	2.53	0.42
2:D:78:LYS:HD2	2:D:78:LYS:HA	1.70	0.42
2:C:107:ASN:O	2:C:108:VAL:C	2.59	0.41
3:E:122:ASP:CA	3:E:126:ARG:H	2.33	0.41
2:C:108:VAL:HB	2:C:109:ASN:H	1.65	0.41
1:A:84:ILE:HD12	1:A:164:PRO:HB3	2.02	0.41
1:A:144:GLN:HE21	1:A:164:PRO:C	2.24	0.41
2:D:304:LEU:HD12	2:D:304:LEU:HA	1.91	0.41
2:C:256:LEU:HD13	2:D:256:LEU:HB3	2.03	0.41
1:B:54:PRO:HG3	1:B:59:ILE:HD12	2.02	0.41
1:A:74:VAL:N	1:A:75:PRO:HD2	2.36	0.41
1:B:168:LEU:HD22	1:B:172:LEU:HD13	2.03	0.40
1:B:95:THR:HG22	1:B:96:VAL:H	1.85	0.40
2:D:258:ILE:HG12	2:D:282:PHE:CD2	2.57	0.40
1:A:177:LEU:HD22	1:A:199:LEU:HG	2.03	0.40
1:B:81:ILE:HG22	1:B:158:VAL:HB	2.02	0.40

There are no symmetry-related clashes.

5.3 Torsion angles (i)

5.3.1 Protein backbone (i)

In the following table, the Percentiles column shows the percent Ramachandran outliers of the chain as a percentile score with respect to all PDB entries followed by that with respect to all EM entries.

The Analysed column shows the number of residues for which the backbone conformation was analysed, and the total number of residues.

Mol	Chain	Analysed	Favoured	Allowed	Outliers	Percentiles	
1	A	216/222 (97%)	203 (94%)	13 (6%)	0	100	100

Continued on next page...

For Manuscript Review

Continued from previous page...

Mol	Chain	Analysed	Favoured	Allowed	Outliers	Percentiles	
1	B	216/222 (97%)	204 (94%)	12 (6%)	0	100	100
2	C	297/352 (84%)	244 (82%)	43 (14%)	10 (3%)	3	19
2	D	297/352 (84%)	265 (89%)	31 (10%)	1 (0%)	41	73
3	E	109/419 (26%)	90 (83%)	18 (16%)	1 (1%)	17	51
All	All	1135/1567 (72%)	1006 (89%)	117 (10%)	12 (1%)	18	45

All (12) Ramachandran outliers are listed below:

Mol	Chain	Res	Type
2	C	183	VAL
2	C	199	ASN
2	D	199	ASN
2	C	214	VAL
2	C	108	VAL
2	C	188	PRO
2	C	208	ILE
2	C	209	ASN
3	E	129	GLU
2	C	221	PHE
2	C	226	ALA
2	C	211	ILE

5.3.2 Protein sidechains ⓘ

In the following table, the Percentiles column shows the percent sidechain outliers of the chain as a percentile score with respect to all PDB entries followed by that with respect to all EM entries.

The Analysed column shows the number of residues for which the sidechain conformation was analysed, and the total number of residues.

Mol	Chain	Analysed	Rotameric	Outliers	Percentiles	
1	A	174/181 (96%)	170 (98%)	4 (2%)	50	76
1	B	173/181 (96%)	169 (98%)	4 (2%)	50	76
2	C	138/289 (48%)	127 (92%)	11 (8%)	12	39
2	D	129/289 (45%)	124 (96%)	5 (4%)	32	64
All	All	614/940 (65%)	590 (96%)	24 (4%)	36	64

All (24) residues with a non-rotameric sidechain are listed below:

For Manuscript Review

Mol	Chain	Res	Type
1	A	16	GLN
1	A	73	GLU
1	A	144	GLN
1	A	207	MET
1	B	30	MET
1	B	70	LYS
1	B	174	GLU
1	B	183	PHE
2	C	82	PHE
2	C	107	ASN
2	C	221	PHE
2	C	224	LEU
2	C	227	LEU
2	C	247	PHE
2	C	275	ASP
2	C	292	SER
2	C	309	SER
2	C	330	PHE
2	C	332	GLU
2	D	71	HIS
2	D	76	ASP
2	D	79	SER
2	D	263	ASP
2	D	330	PHE

Sometimes sidechains can be flipped to improve hydrogen bonding and reduce clashes. There are no such sidechains identified.

5.3.3 RNA (i)

There are no RNA molecules in this entry.

5.4 Non-standard residues in protein, DNA, RNA chains (i)

There are no non-standard protein/DNA/RNA residues in this entry.

5.5 Carbohydrates (i)

There are no monosaccharides in this entry.

For Manuscript Review

5.6 Ligand geometry (i)

2 ligands are modelled in this entry.

In the following table, the Counts columns list the number of bonds (or angles) for which Mogul statistics could be retrieved, the number of bonds (or angles) that are observed in the model and the number of bonds (or angles) that are defined in the Chemical Component Dictionary. The Link column lists molecule types, if any, to which the group is linked. The Z score for a bond length (or angle) is the number of standard deviations the observed value is removed from the expected value. A bond length (or angle) with $|Z| > 2$ is considered an outlier worth inspection. RMSZ is the root-mean-square of all Z scores of the bond lengths (or angles).

Mol	Type	Chain	Res	Link	Bond lengths			Bond angles		
					Counts	RMSZ	$\#\|Z\| > 2$	Counts	RMSZ	$\#\|Z\| > 2$
4	ATP	B	301	-	26,33,33	0.75	0	31,52,52	0.80	1 (3%)
4	ATP	A	301	-	26,33,33	0.73	0	31,52,52	0.78	1 (3%)

In the following table, the Chirals column lists the number of chiral outliers, the number of chiral centers analysed, the number of these observed in the model and the number defined in the Chemical Component Dictionary. Similar counts are reported in the Torsion and Rings columns. '-' means no outliers of that kind were identified.

Mol	Type	Chain	Res	Link	Chirals	Torsions	Rings
4	ATP	B	301	-	-	0/18/38/38	0/3/3/3
4	ATP	A	301	-	-	2/18/38/38	0/3/3/3

There are no bond length outliers.

All (2) bond angle outliers are listed below:

Mol	Chain	Res	Type	Atoms	Z	Observed(^{o})	Ideal(^{o})
4	B	301	ATP	C5-C6-N6	2.30	123.85	120.35
4	A	301	ATP	C5-C6-N6	2.27	123.80	120.35

There are no chirality outliers.

All (2) torsion outliers are listed below:

Mol	Chain	Res	Type	Atoms
4	A	301	ATP	PA-O3A-PB-O1B
4	A	301	ATP	PA-O3A-PB-O2B

There are no ring outliers.

No monomer is involved in short contacts.

For Manuscript Review

The following is a two-dimensional graphical depiction of Mogul quality analysis of bond lengths, bond angles, torsion angles, and ring geometry for all instances of the Ligand of Interest. In addition, ligands with molecular weight > 250 and outliers as shown on the validation Tables will also be included. For torsion angles, if less then 5% of the Mogul distribution of torsion angles is within 10 degrees of the torsion angle in question, then that torsion angle is considered an outlier. Any bond that is central to one or more torsion angles identified as an outlier by Mogul will be highlighted in the graph. For rings, the root-mean-square deviation (RMSD) between the ring in question and similar rings identified by Mogul is calculated over all ring torsion angles. If the average RMSD is greater than 60 degrees and the minimal RMSD between the ring in question and any Mogul-identified rings is also greater than 60 degrees, then that ring is considered an outlier. The outliers are highlighted in purple. The color gray indicates Mogul did not find sufficient equivalents in the CSD to analyse the geometry.

Ligand ATP B 301

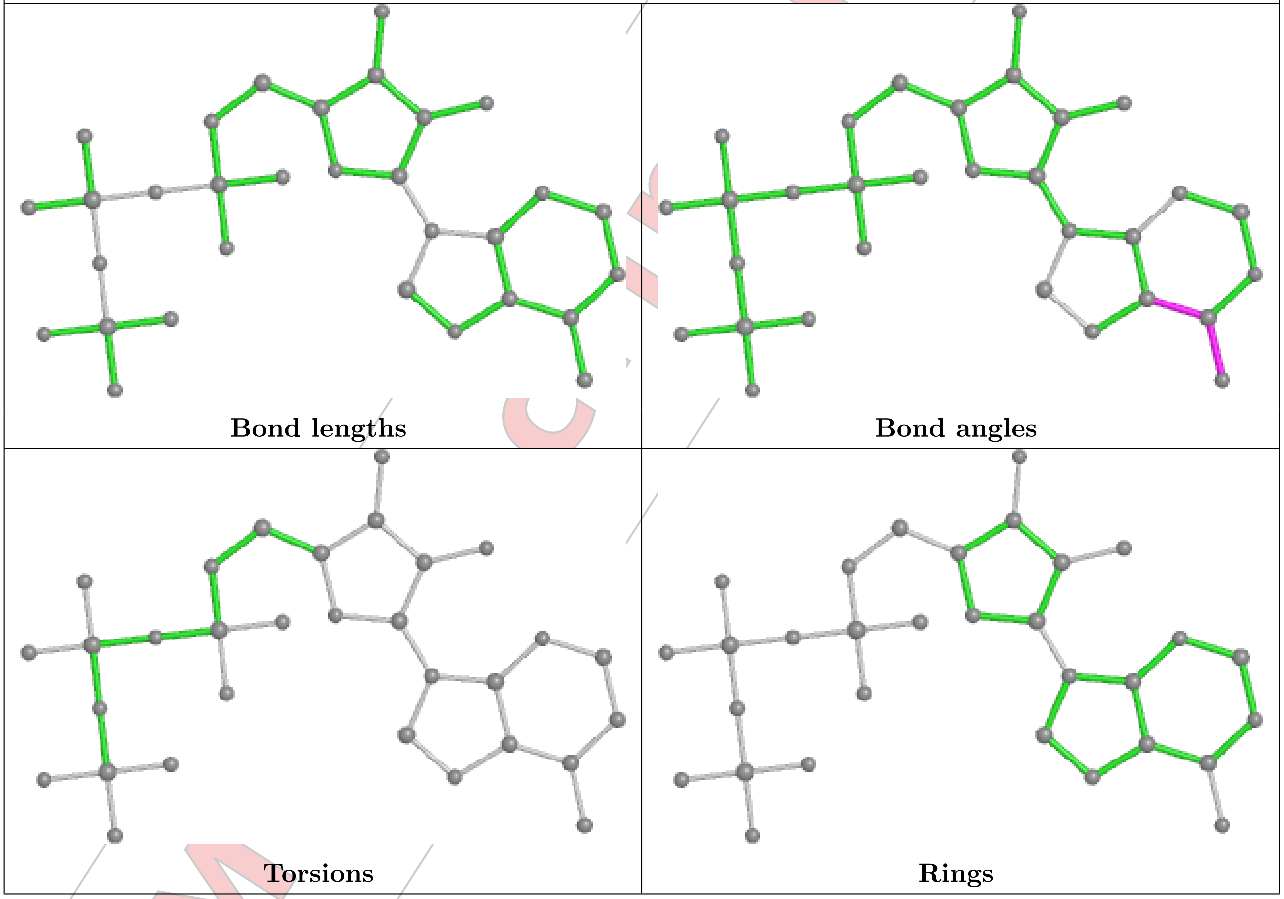

For Manuscript Review

Ligand ATP A 301

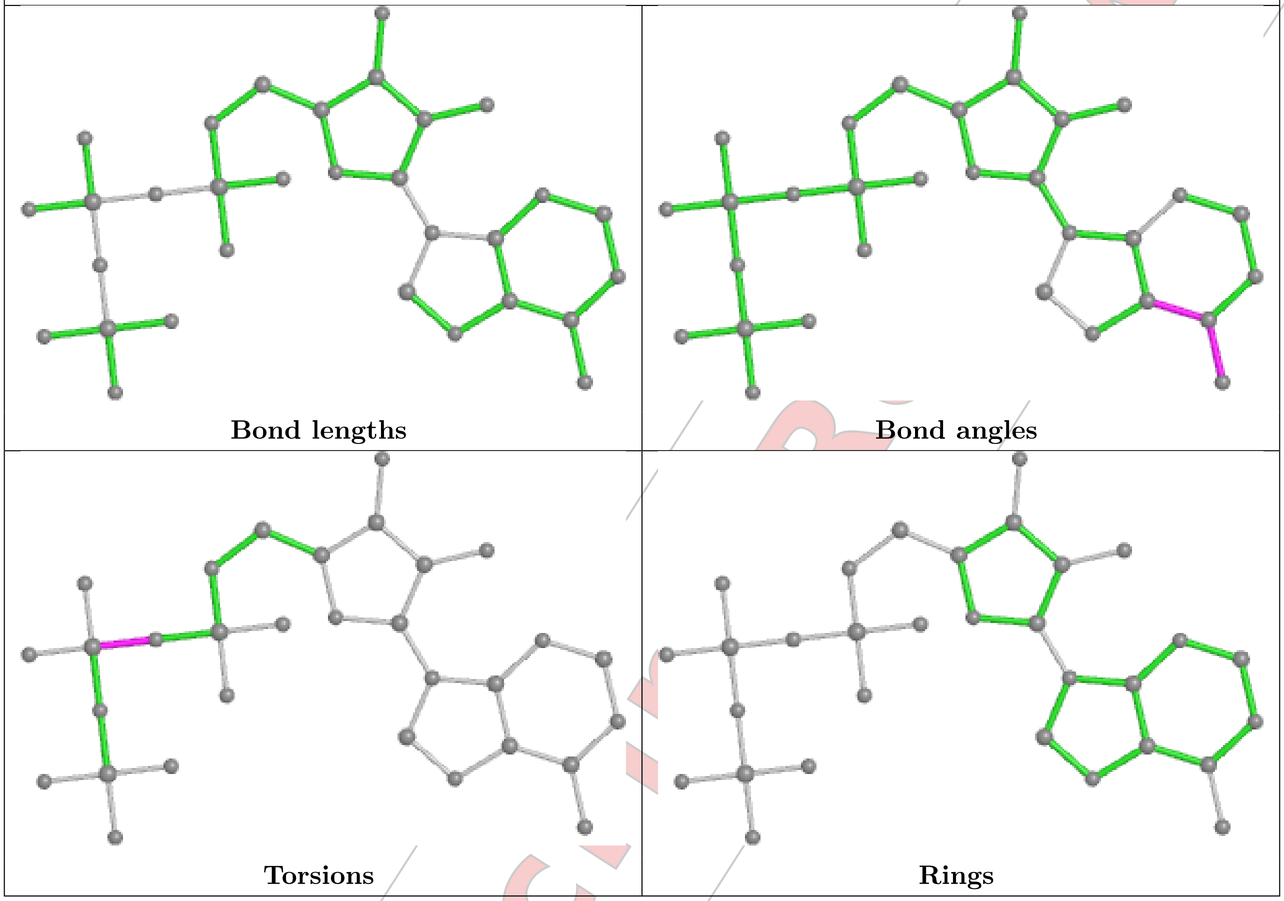

5.7 Other polymers (i)

There are no such residues in this entry.

5.8 Polymer linkage issues (i)

There are no chain breaks in this entry.

For Manuscript Review

6 Map visualisation (i)

This section contains visualisations of the EMDB entry EMD-38906. These allow visual inspection of the internal detail of the map and identification of artifacts.

Images derived from a raw map, generated by summing the deposited half-maps, are presented below the corresponding image components of the primary map to allow further visual inspection and comparison with those of the primary map.

6.1 Orthogonal projections (i)

6.1.1 Primary map

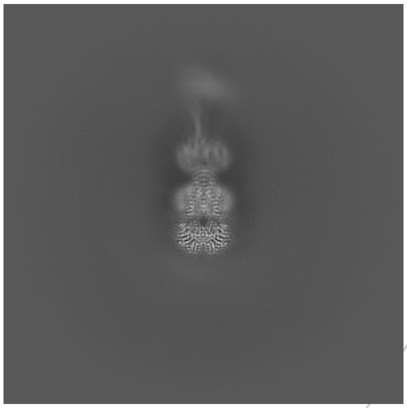
X

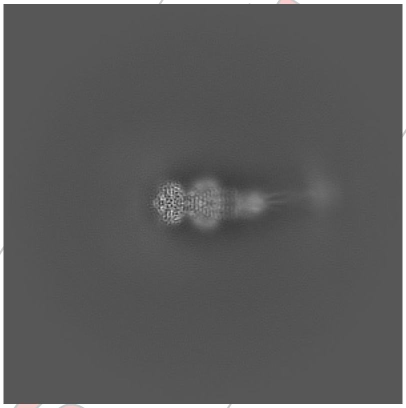
Y

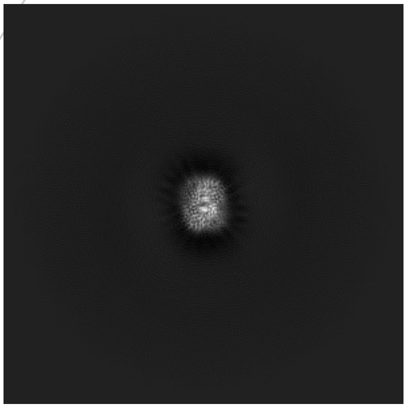
Z

6.1.2 Raw map

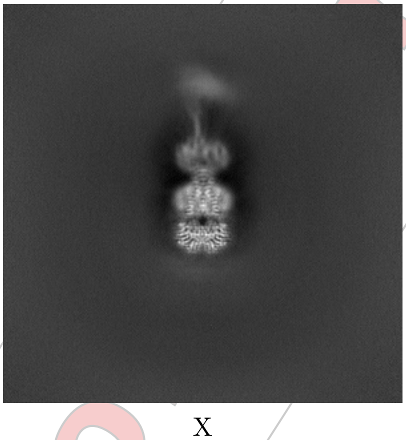
X

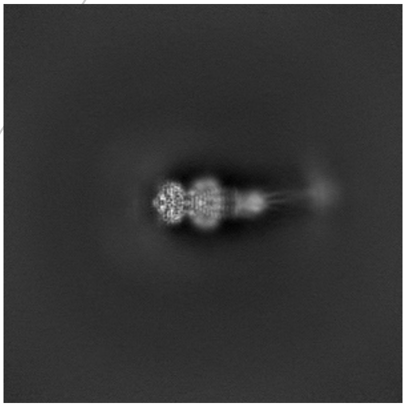
Y

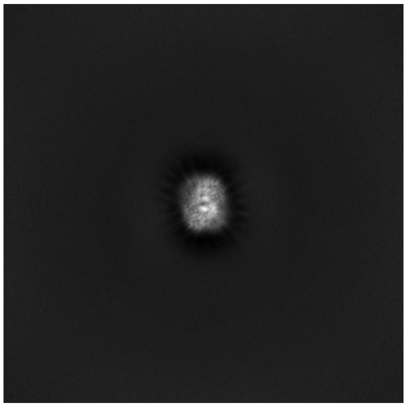
Z

The images above show the map projected in three orthogonal directions.

For Manuscript Review

6.2 Central slices (i)

6.2.1 Primary map

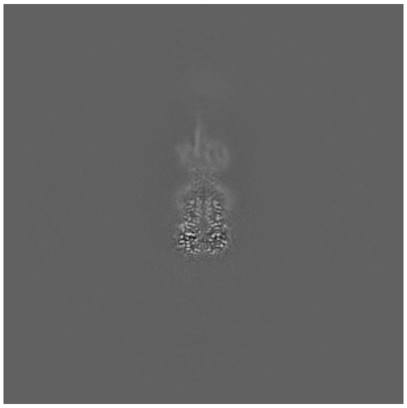
X Index: 288

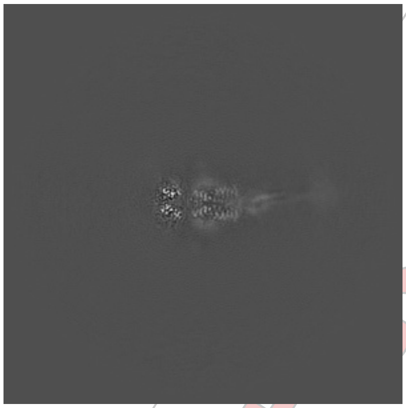
Y Index: 288

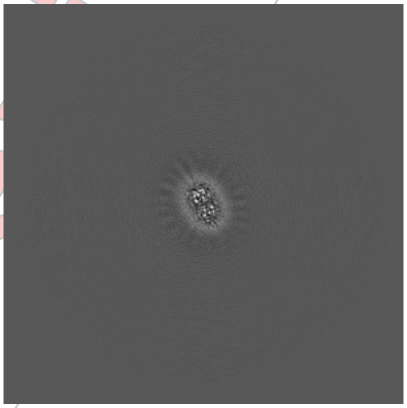
Z Index: 288

6.2.2 Raw map

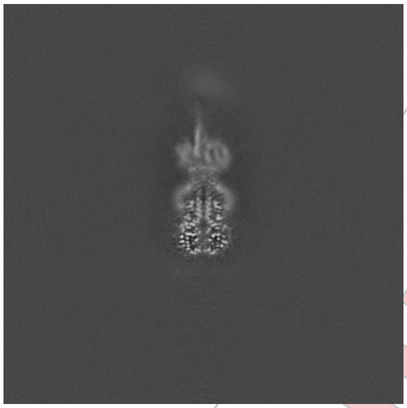
X Index: 288

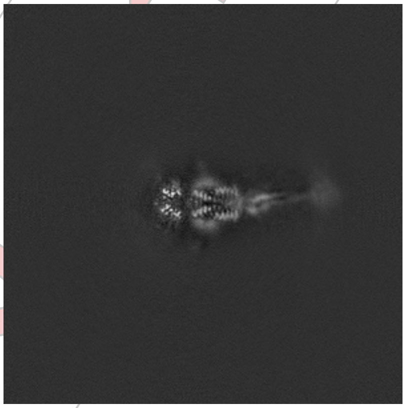
Y Index: 288

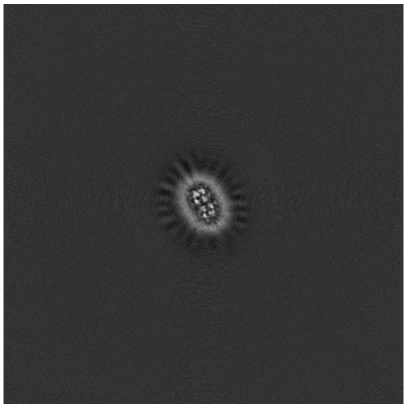
Z Index: 288

The images above show central slices of the map in three orthogonal directions.

For Manuscript Review

6.3 Largest variance slices (i)

6.3.1 Primary map

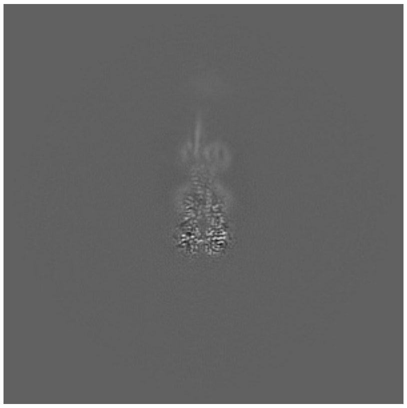

X Index: 291

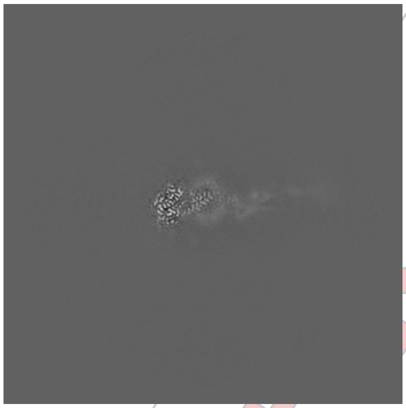

Y Index: 271

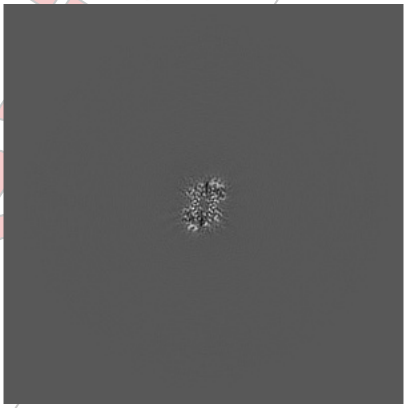

Z Index: 242

6.3.2 Raw map

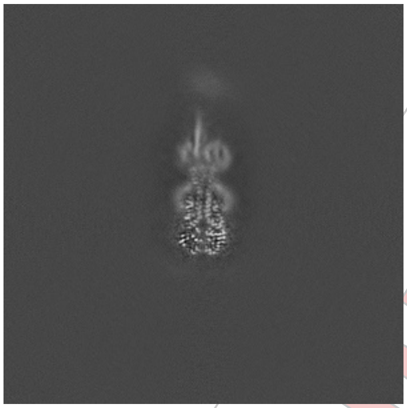

X Index: 290

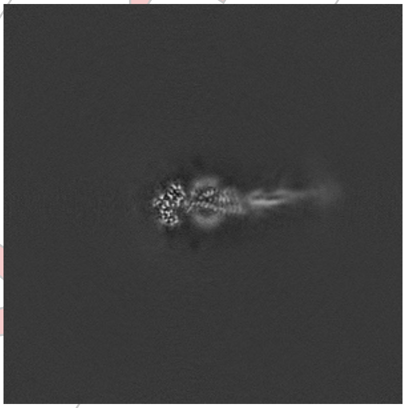

Y Index: 278

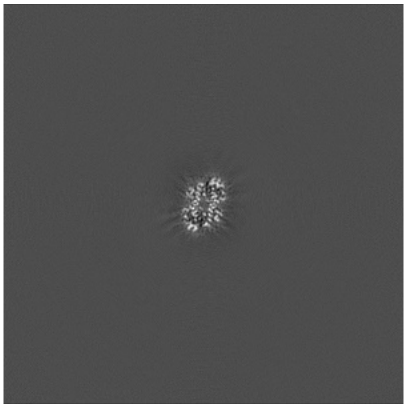

Z Index: 242

The images above show the largest variance slices of the map in three orthogonal directions.

For Manuscript Review

6.4 Orthogonal standard-deviation projections (False-color) (i)

6.4.1 Primary map

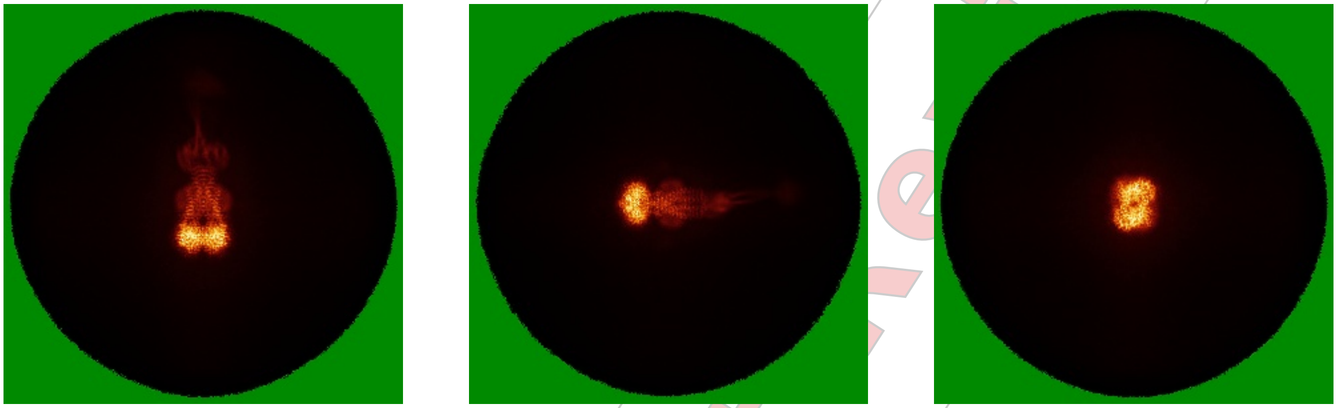

X Y Z

6.4.2 Raw map

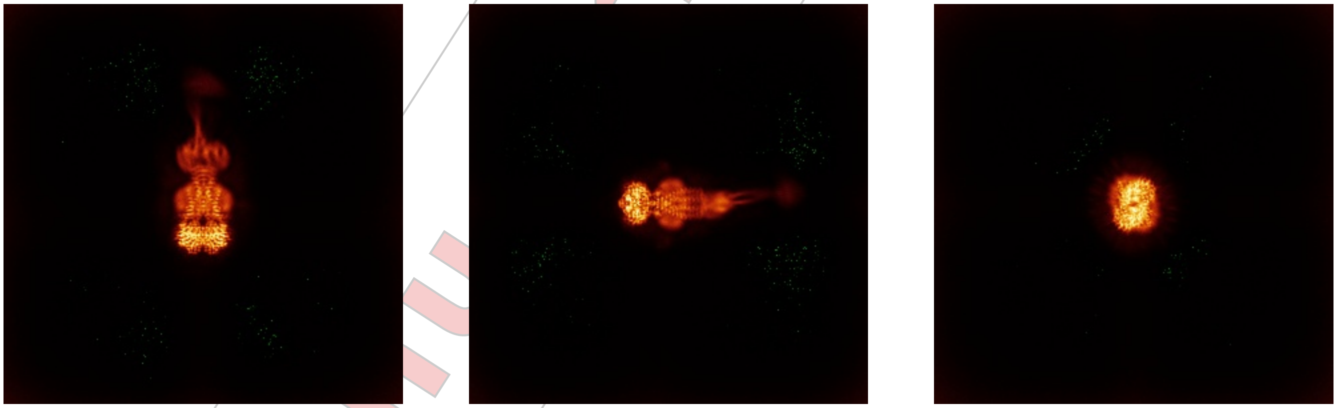

X Y Z

The images above show the map standard deviation projections with false color in three orthogonal directions. Minimum values are shown in green, max in blue, and dark to light orange shades represent small to large values respectively.

For Manuscript Review

6.5 Orthogonal surface views (i)

6.5.1 Primary map

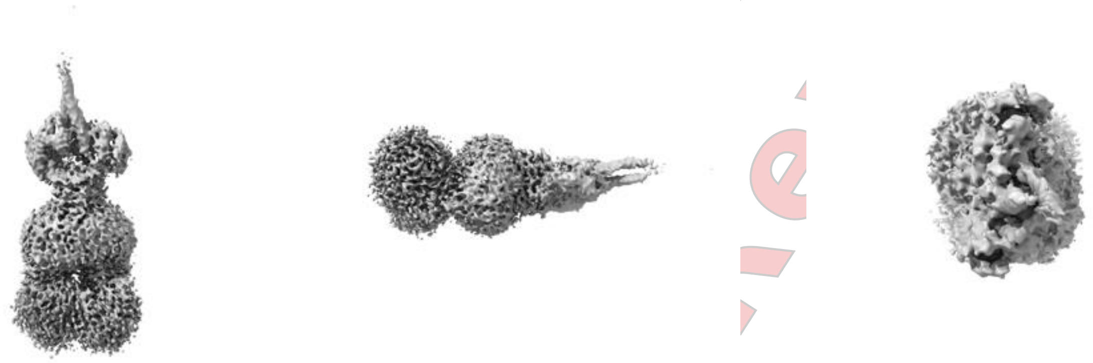

X Y Z

The images above show the 3D surface view of the map at the recommended contour level 0.1. These images, in conjunction with the slice images, may facilitate assessment of whether an appropriate contour level has been provided.

6.5.2 Raw map

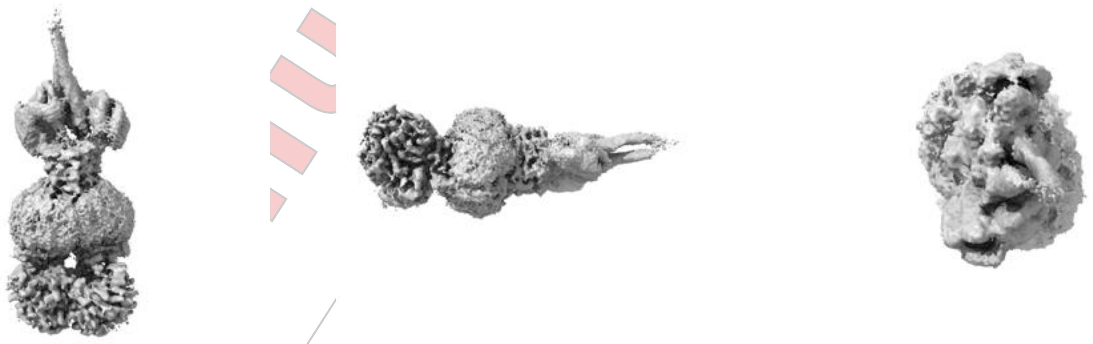

X Y Z

These images show the 3D surface of the raw map. The raw map's contour level was selected so that its surface encloses the same volume as the primary map does at its recommended contour level.

For Manuscript Review

6.6 Mask visualisation (i)

This section shows the 3D surface view of the primary map at 50% transparency overlaid with the specified mask at 0% transparency

A mask typically either:

- Encompasses the whole structure
- Separates out a domain, a functional unit, a monomer or an area of interest from a larger structure

6.6.1 D_1300044789_em-mask-volume_P1.map.V2 (i)

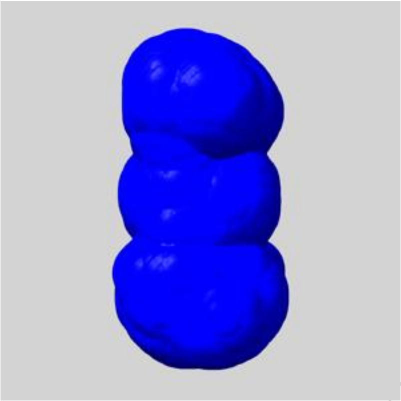

X

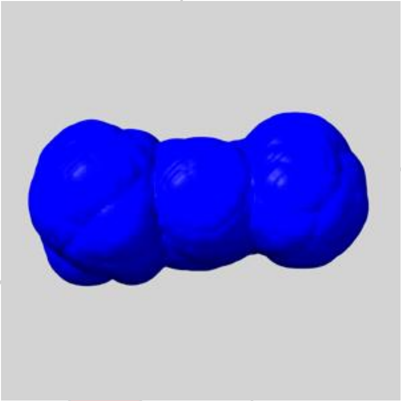

Y

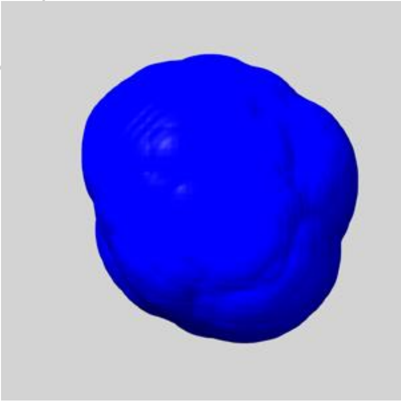

Z

For Manuscript Review

7 Map analysis (i)

This section contains the results of statistical analysis of the map.

7.1 Map-value distribution (i)

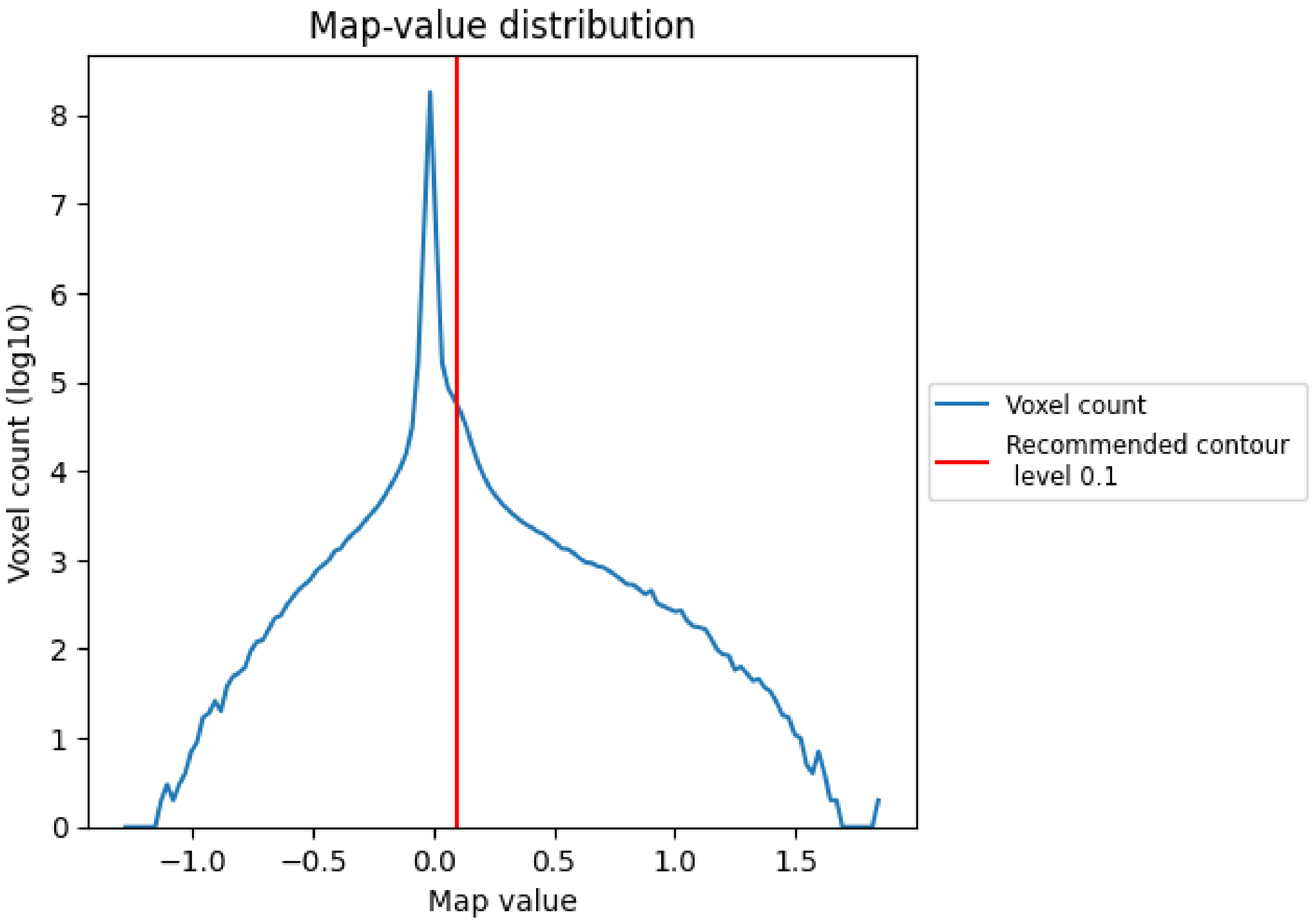

The map-value distribution is plotted in 128 intervals along the x-axis. The y-axis is logarithmic. A spike in this graph at zero usually indicates that the volume has been masked.

7.2 Volume estimate (i)

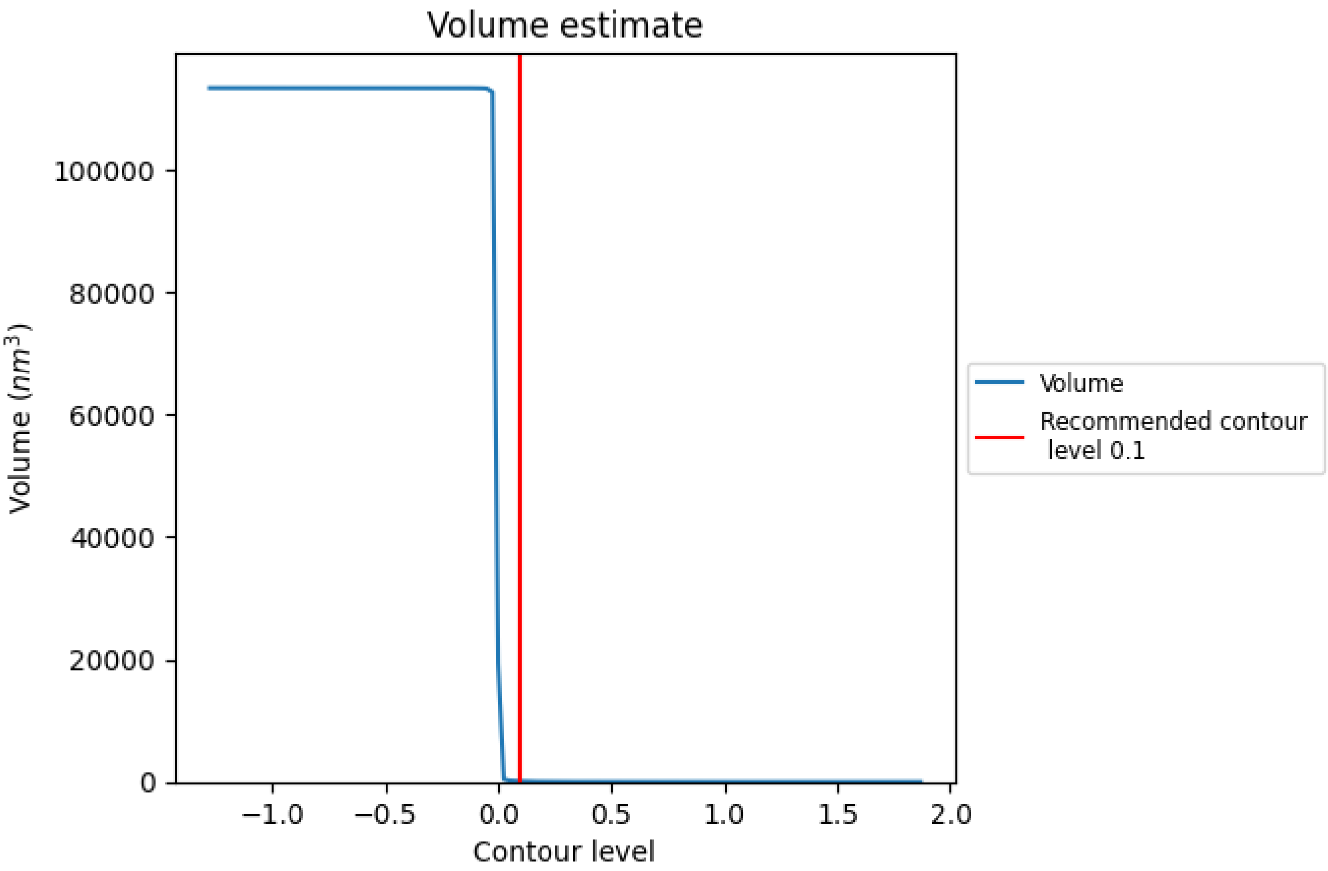

The volume at the recommended contour level is 118 nm^3; this corresponds to an approximate mass of 107 kDa.

The volume estimate graph shows how the enclosed volume varies with the contour level. The recommended contour level is shown as a vertical line and the intersection between the line and the curve gives the volume of the enclosed surface at the given level.

For Manuscript Review

7.3 Rotationally averaged power spectrum (i)

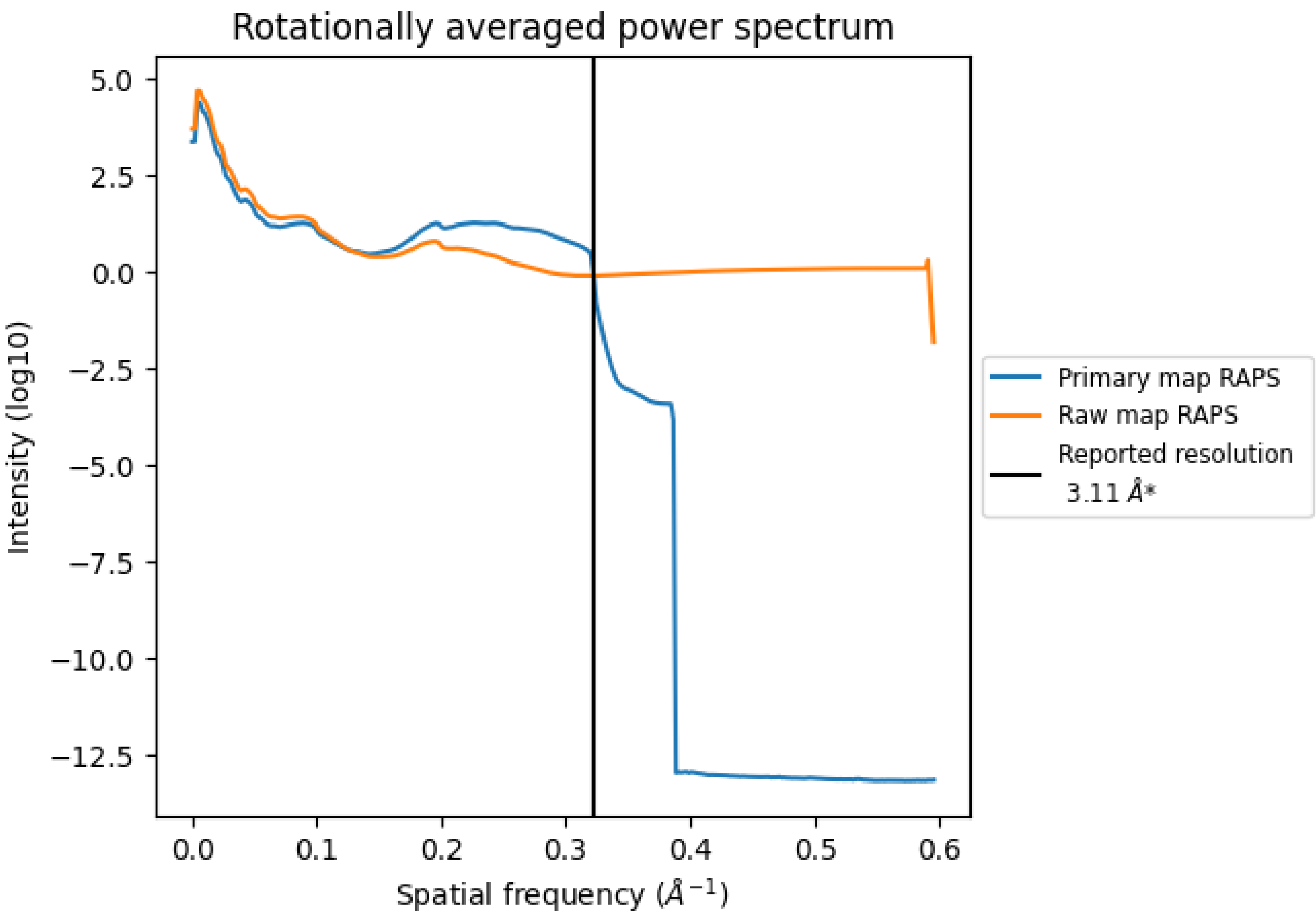

*Reported resolution corresponds to spatial frequency of 0.322 Å^{-1}

For Manuscript Review

8 Fourier-Shell correlation ⓘ

Fourier-Shell Correlation (FSC) is the most commonly used method to estimate the resolution of single-particle and subtomogram-averaged maps. The shape of the curve depends on the imposed symmetry, mask and whether or not the two 3D reconstructions used were processed from a common reference. The reported resolution is shown as a black line. A curve is displayed for the half-bit criterion in addition to lines showing the 0.143 gold standard cut-off and 0.5 cut-off.

8.1 FSC ⓘ

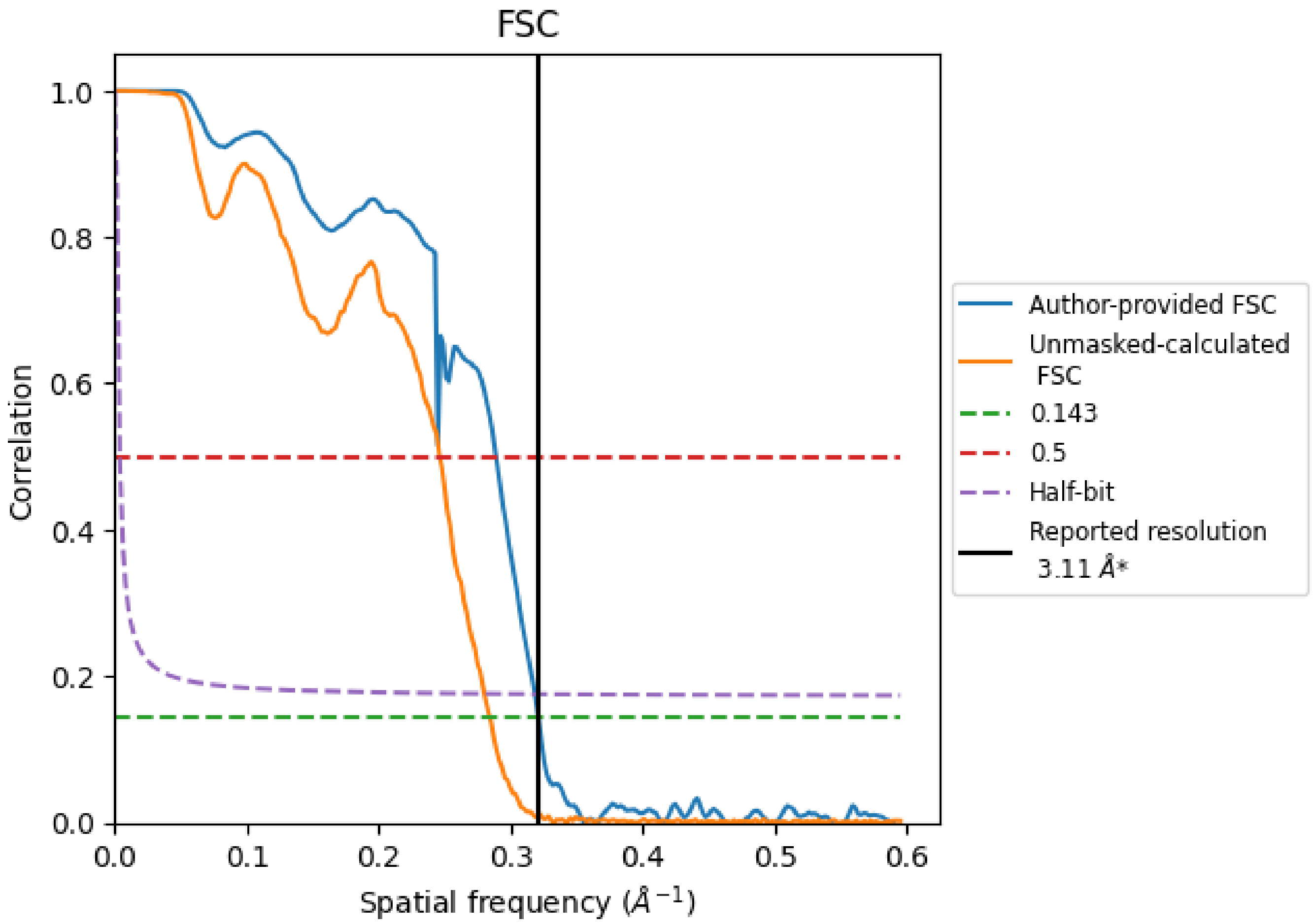

*Reported resolution corresponds to spatial frequency of 0.322 Å^{-1}

For Manuscript Review

8.2 Resolution estimates (i)

Resolution estimate (Å)	Estimation criterion (FSC cut-off)		
	0.143	0.5	Half-bit
Reported by author	3.11	-	-
Author-provided FSC curve	3.12	3.46	3.14
Unmasked-calculated*	3.52	4.06	3.57

*Resolution estimate based on FSC curve calculated by comparison of deposited half-maps. The value from deposited half-maps intersecting FSC 0.143 CUT-OFF 3.52 differs from the reported value 3.11 by more than 10 %

For Manuscript Review

9 Map-model fit (i)

This section contains information regarding the fit between EMDB map EMD-38906 and PDB model 8Y3X. Per-residue inclusion information can be found in section 3 on page 5.

9.1 Map-model overlay (i)

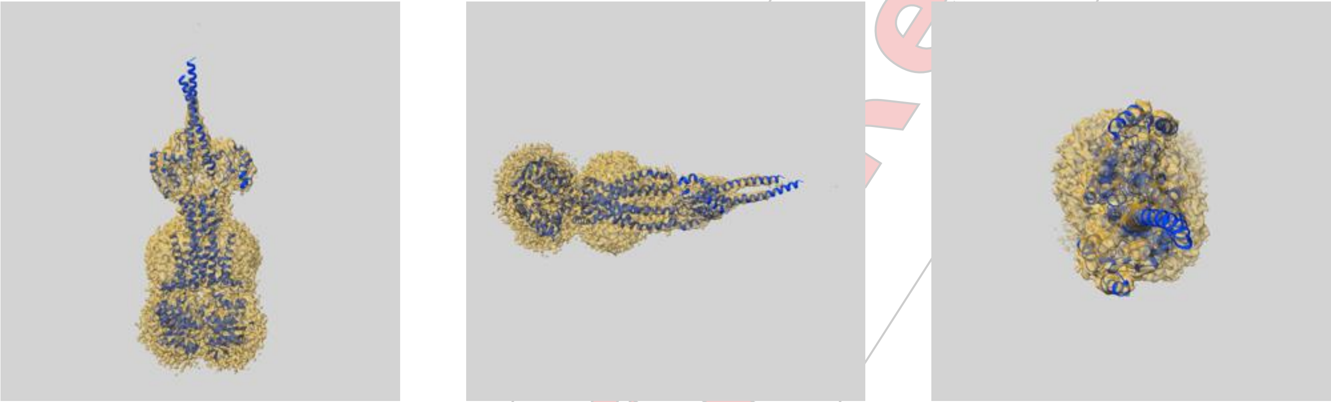

X Y Z

The images above show the 3D surface view of the map at the recommended contour level 0.1 at 50% transparency in yellow overlaid with a ribbon representation of the model coloured in blue. These images allow for the visual assessment of the quality of fit between the atomic model and the map.

For Manuscript Review

9.2 Q-score mapped to coordinate model (i)

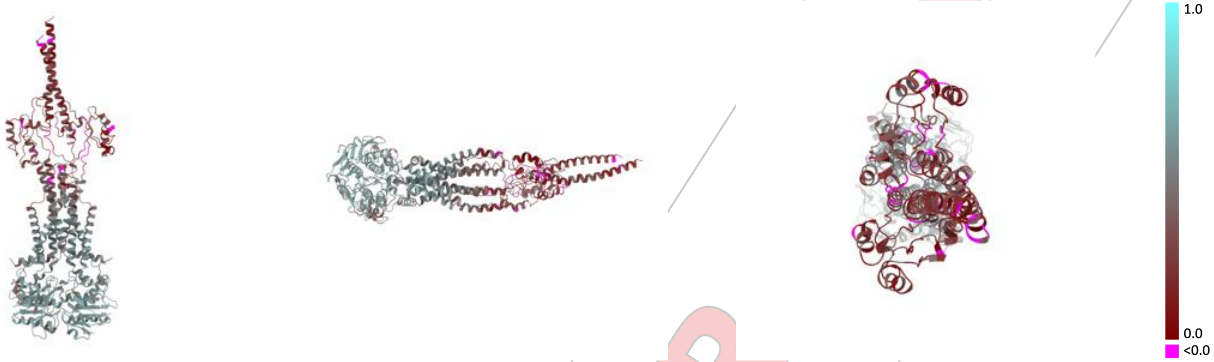

X Y Z

The images above show the model with each residue coloured according its Q-score. This shows their resolvability in the map with higher Q-score values reflecting better resolvability. Please note: Q-score is calculating the resolvability of atoms, and thus high values are only expected at resolutions at which atoms can be resolved. Low Q-score values may therefore be expected for many entries.

9.3 Atom inclusion mapped to coordinate model (i)

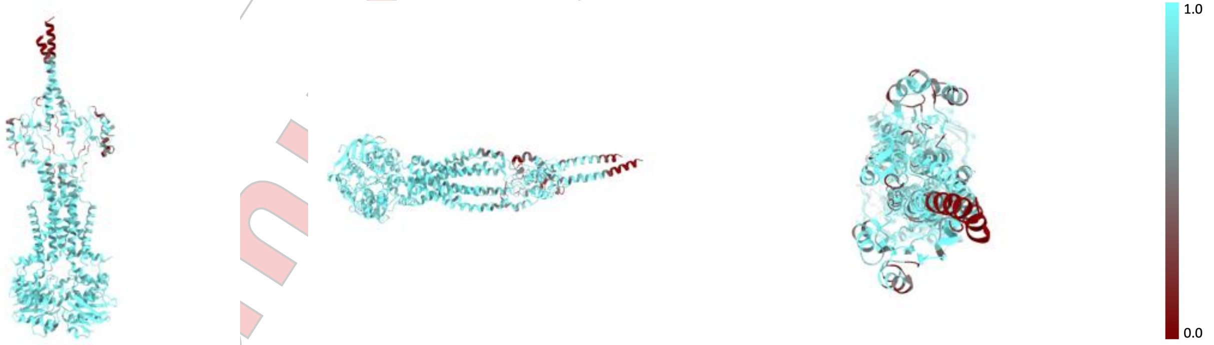

X Y Z

The images above show the model with each residue coloured according to its atom inclusion. This shows to what extent they are inside the map at the recommended contour level (0.1).

For Manuscript Review

9.4 Atom inclusion (i)

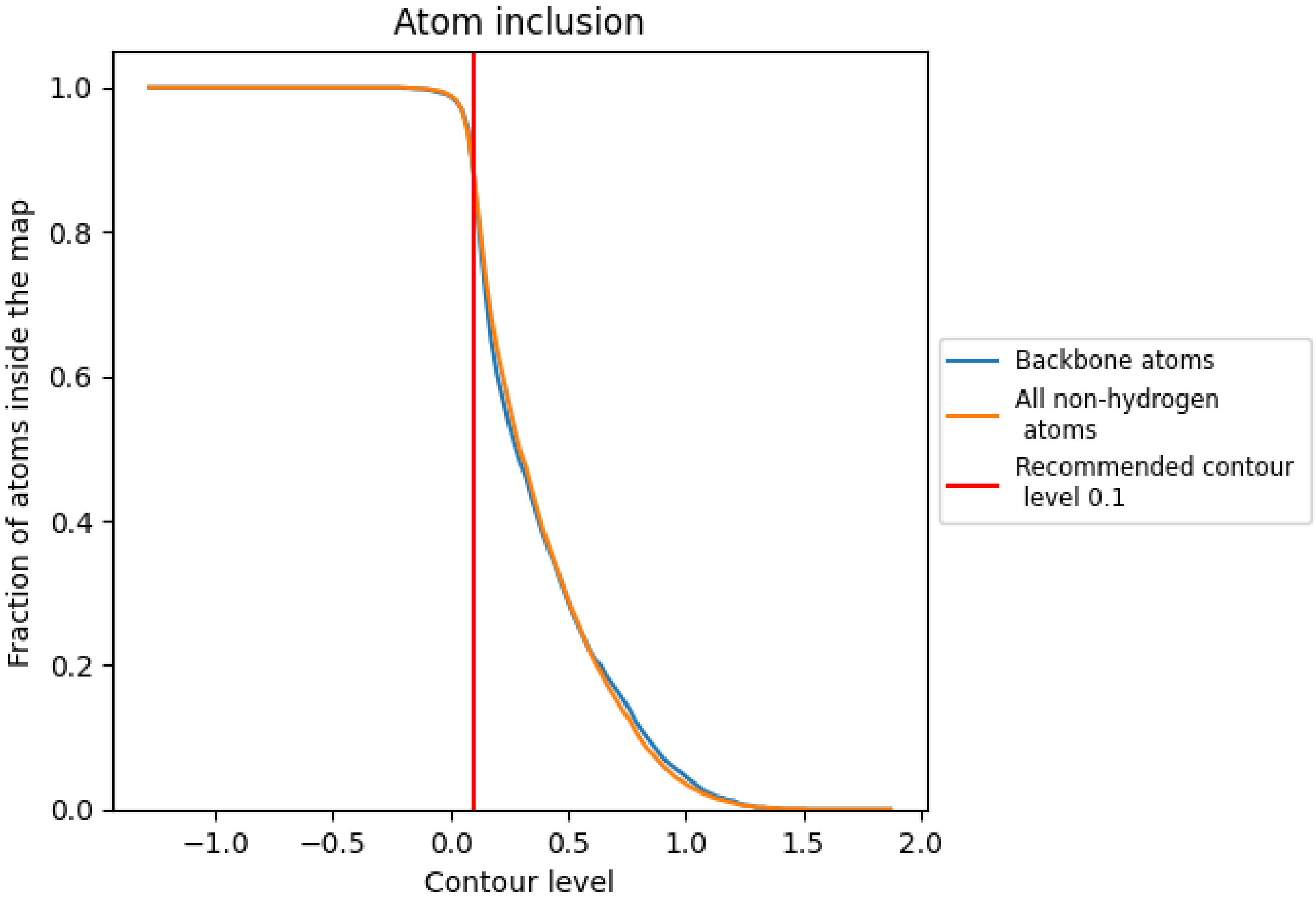

At the recommended contour level, 88% of all backbone atoms, 88% of all non-hydrogen atoms, are inside the map.

For Manuscript Review

9.5 Map-model fit summary (i)

The table lists the average atom inclusion at the recommended contour level (0.1) and Q-score for the entire model and for each chain.

Chain	Atom inclusion	Q-score
All	0.8780	0.4440
A	0.9470	0.5570
B	0.9450	0.5490
C	0.8480	0.3800
D	0.8560	0.3850
E	0.6440	0.1960

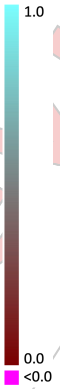

For Manuscript Review
